# Supplementary figures and images for: A microglia clonal inflammatory disorder in Alzheimer’s disease
Source: eLife. 2025 Mar 14;13:RP96519. doi: 10.7554/eLife.96519 (PMC11908784; doi:10.7554/eLife.96519)

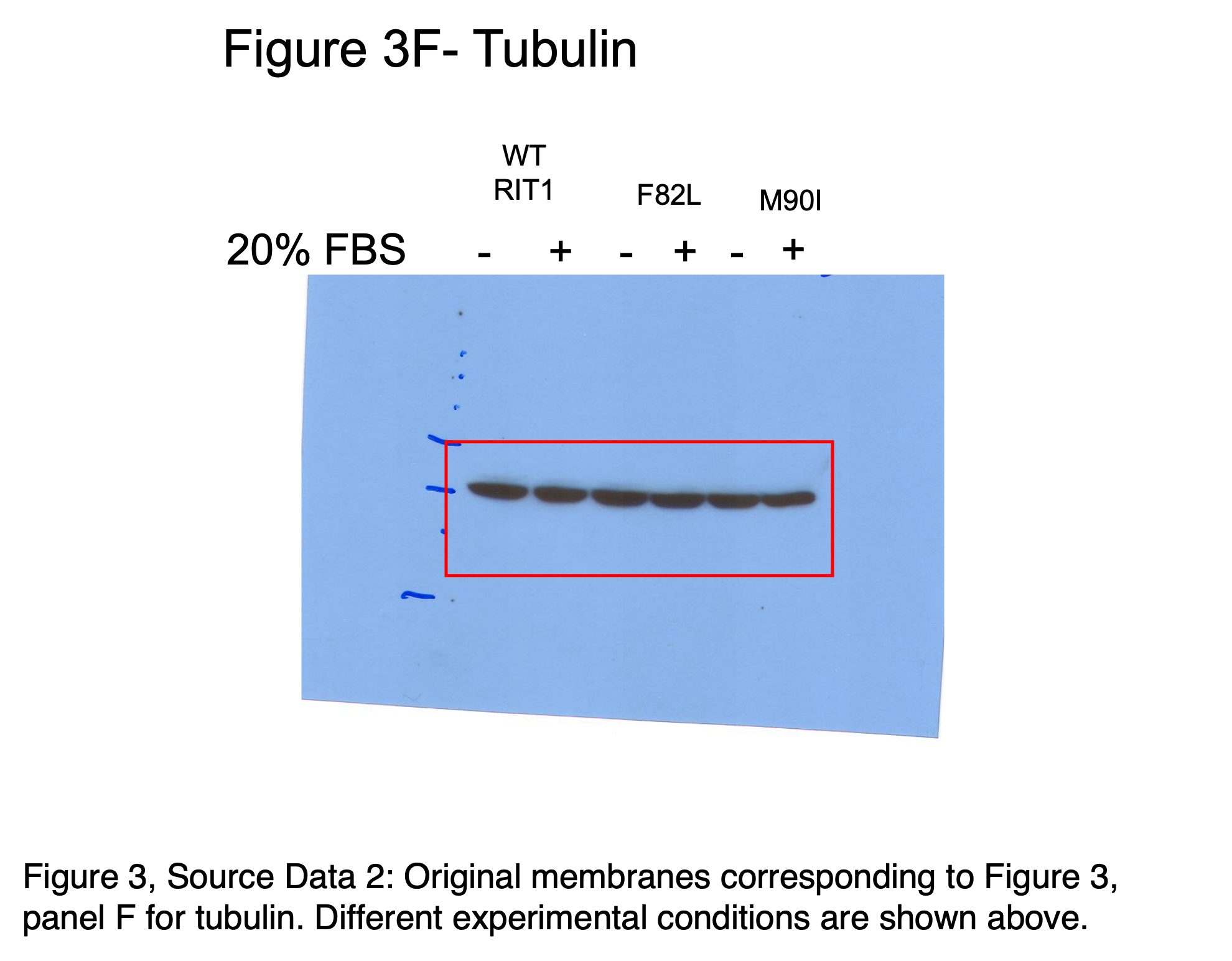

Supplement: Figure 3—source data 2. [file elife-96519-fig3-data2.zip › Figure 3F- Tubulin .png]

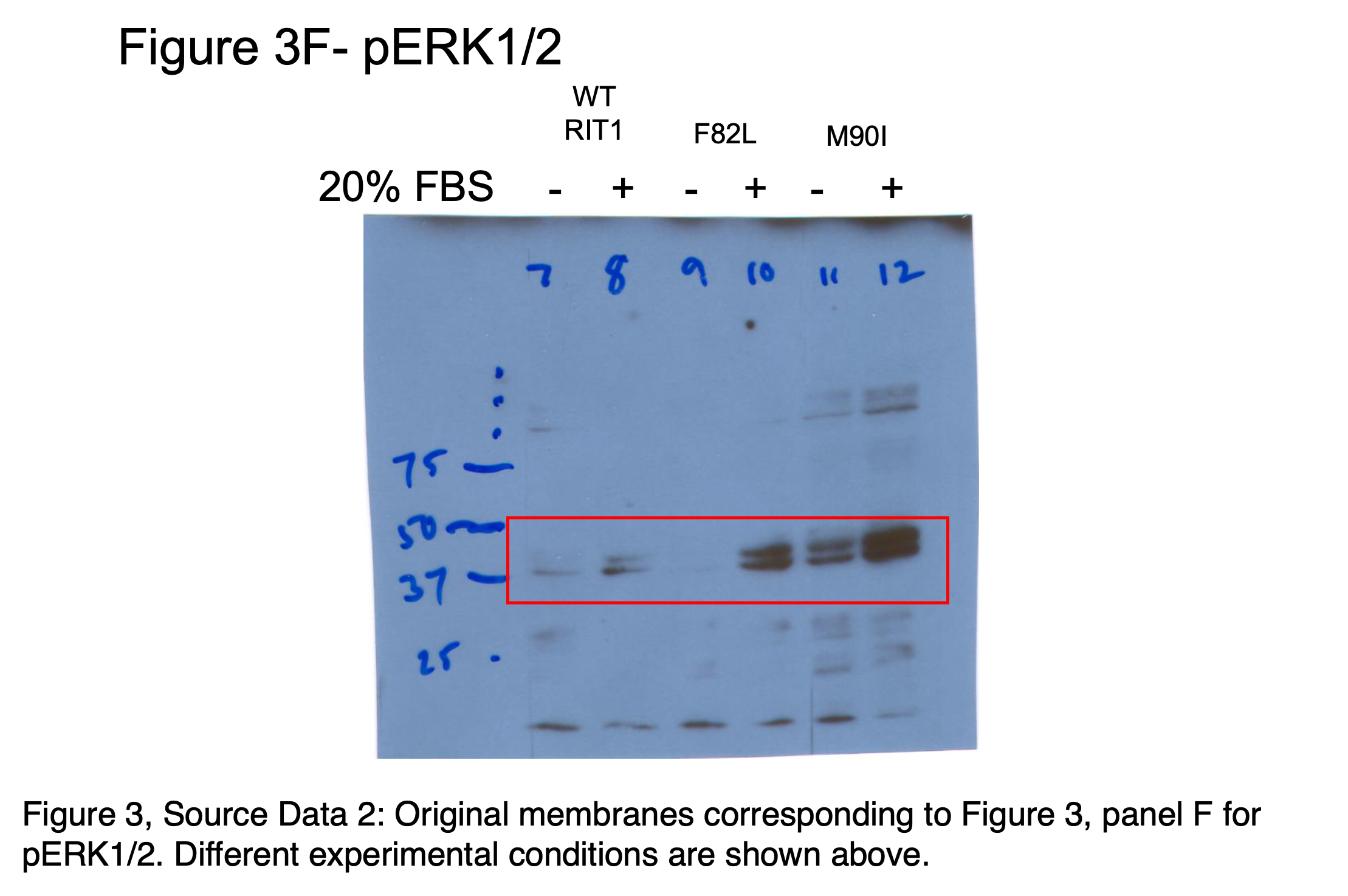

Supplement: Figure 3—source data 2. [file elife-96519-fig3-data2.zip › Figure 3F- pERK1:2 .png]

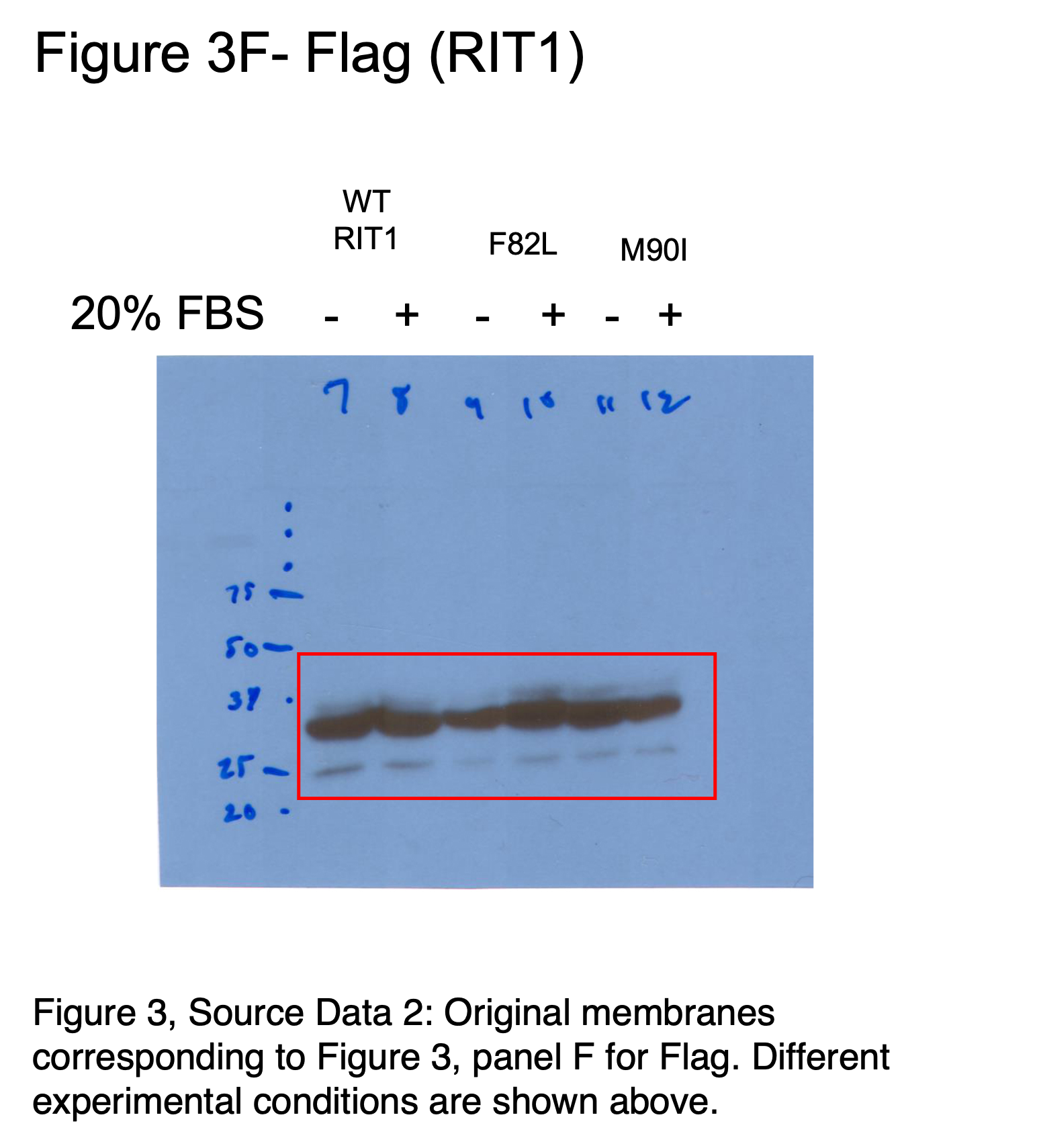

Supplement: Figure 3—source data 2. [file elife-96519-fig3-data2.zip › Figure 3F- Flag (RIT1) .png]

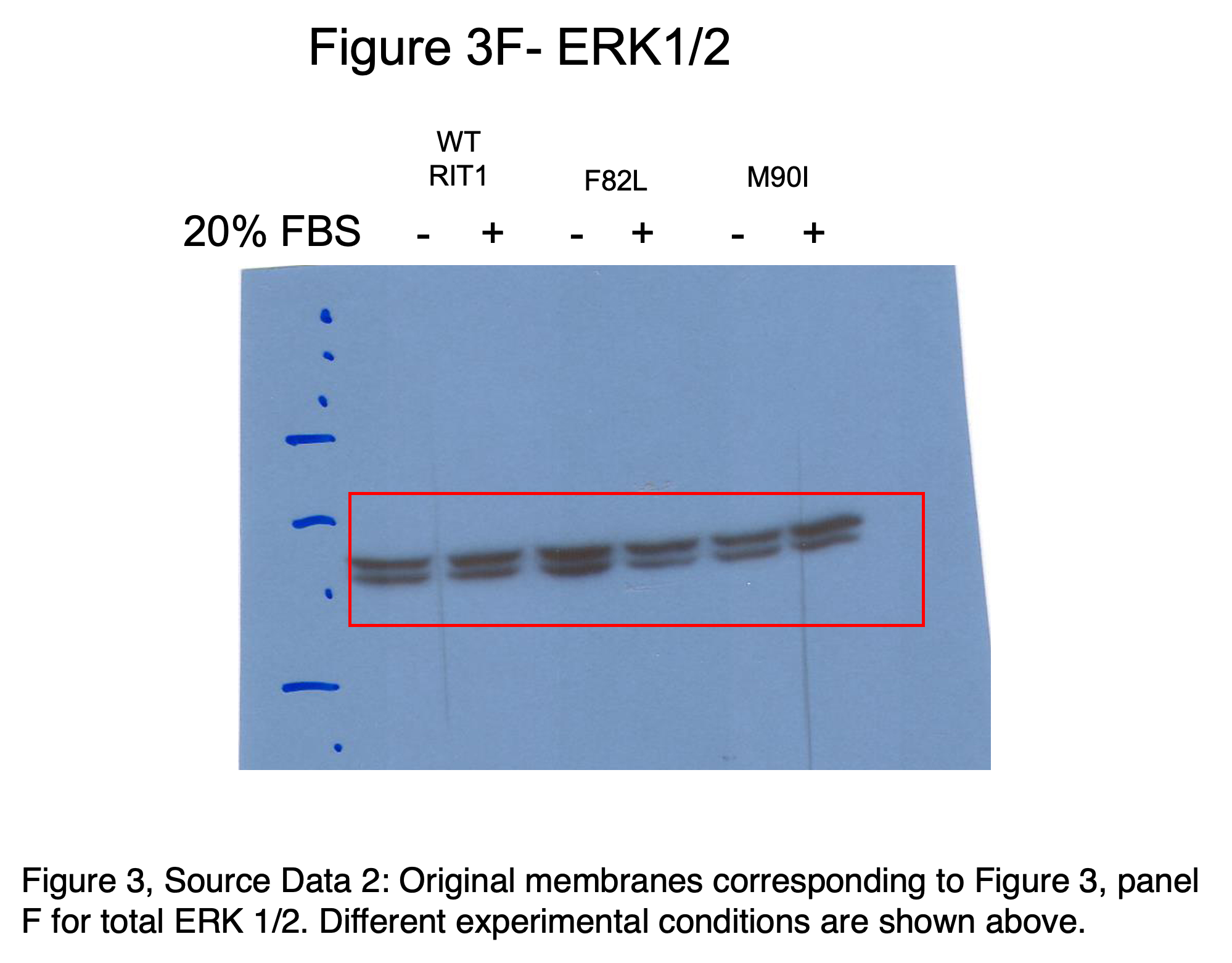

Supplement: Figure 3—source data 2. [file elife-96519-fig3-data2.zip › Figure 3F- ERK1:2 .png]

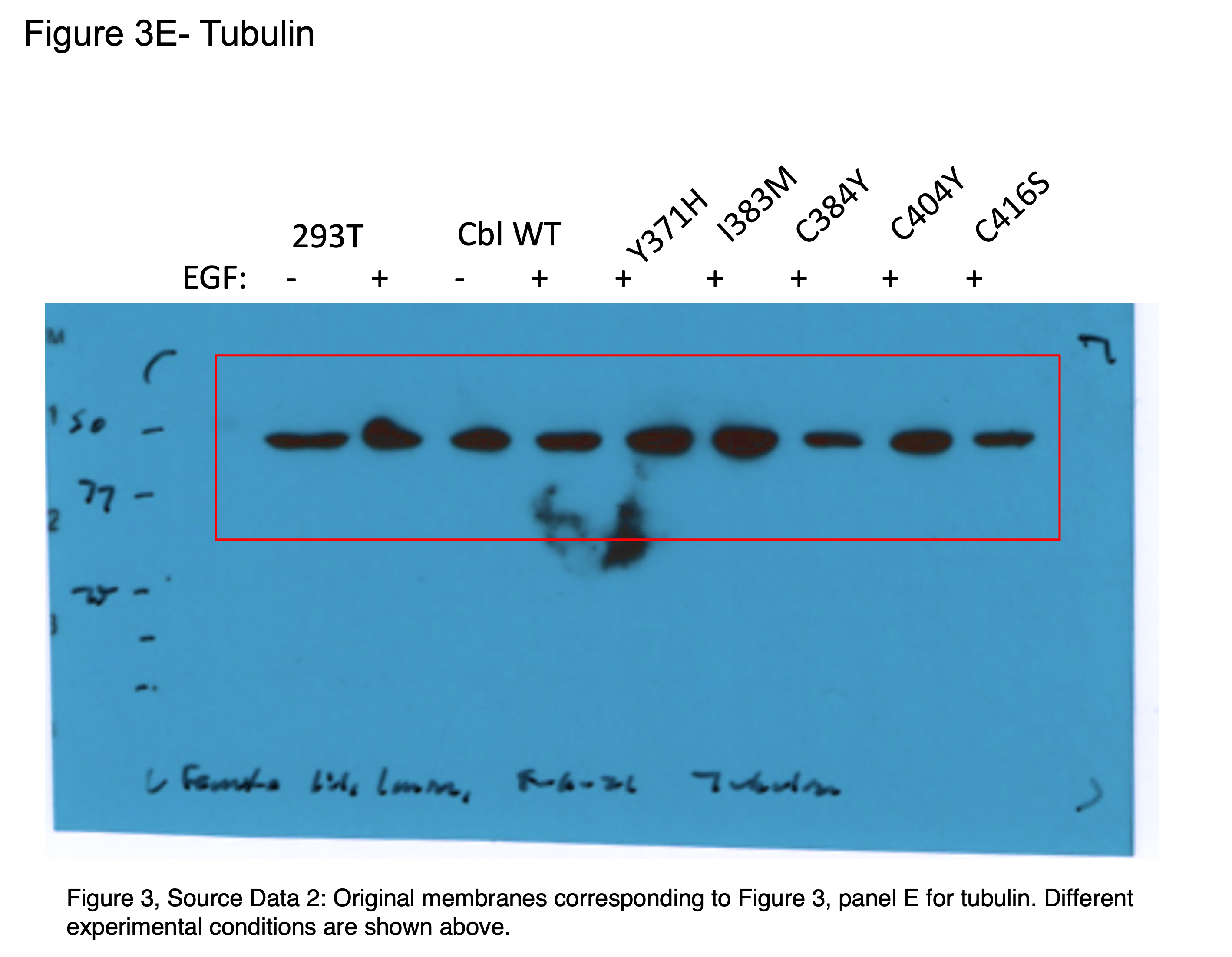

Supplement: Figure 3—source data 2. [file elife-96519-fig3-data2.zip › Figure 3E- Tubulin .png]

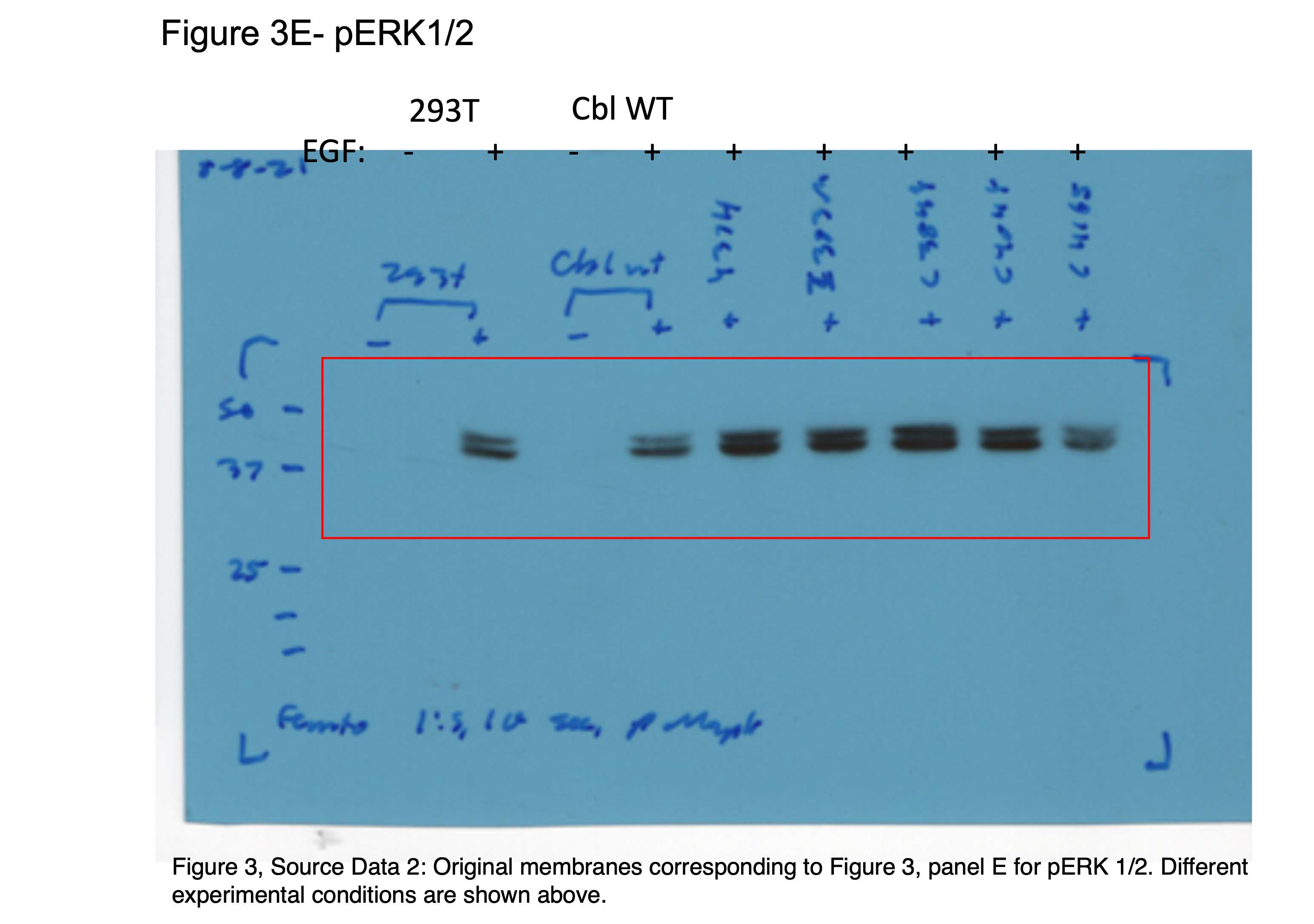

Supplement: Figure 3—source data 2. [file elife-96519-fig3-data2.zip › Figure 3E- pERK1:2 .png]

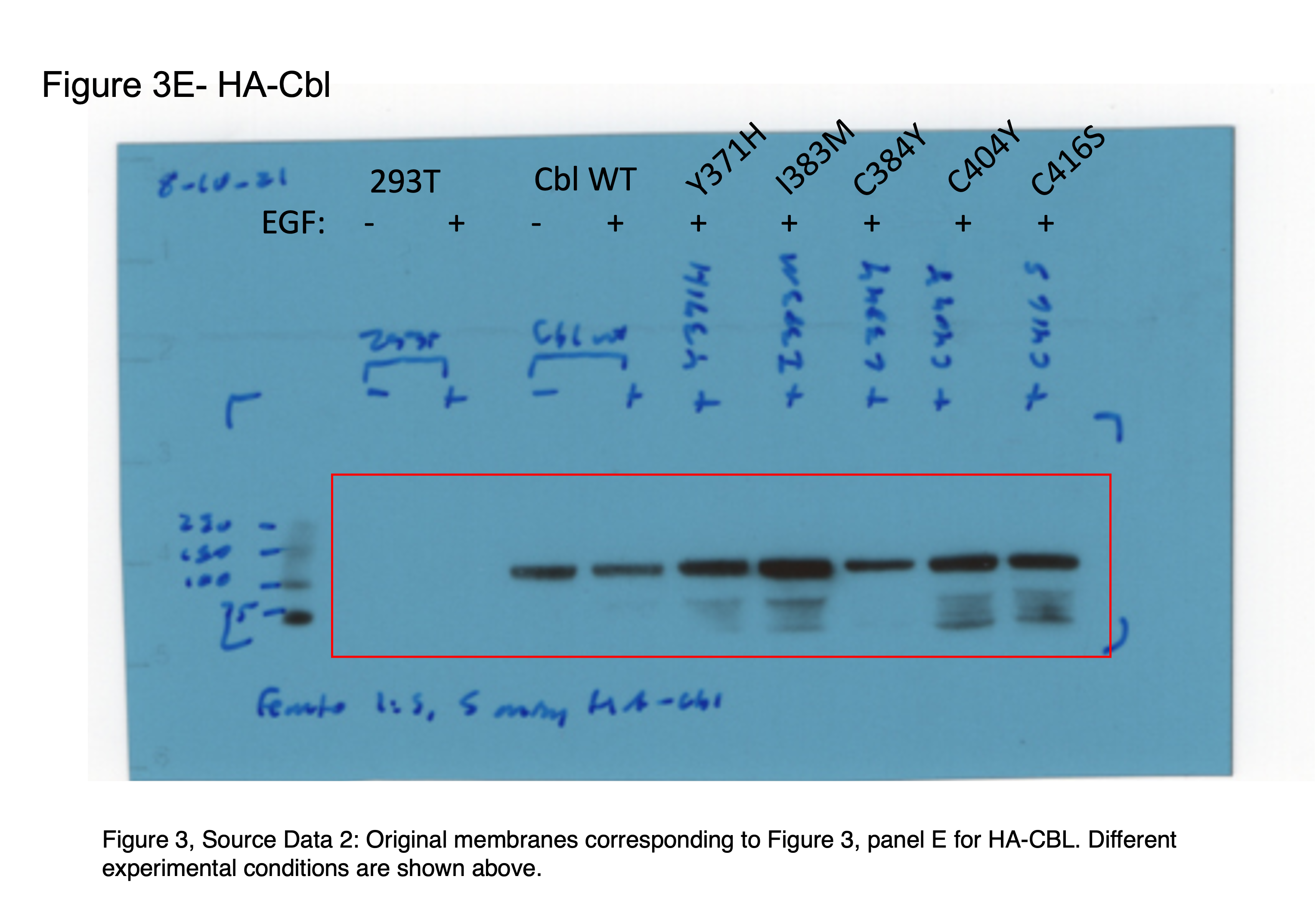

Supplement: Figure 3—source data 2. [file elife-96519-fig3-data2.zip › Figure 3E- HA-Cbl .png]

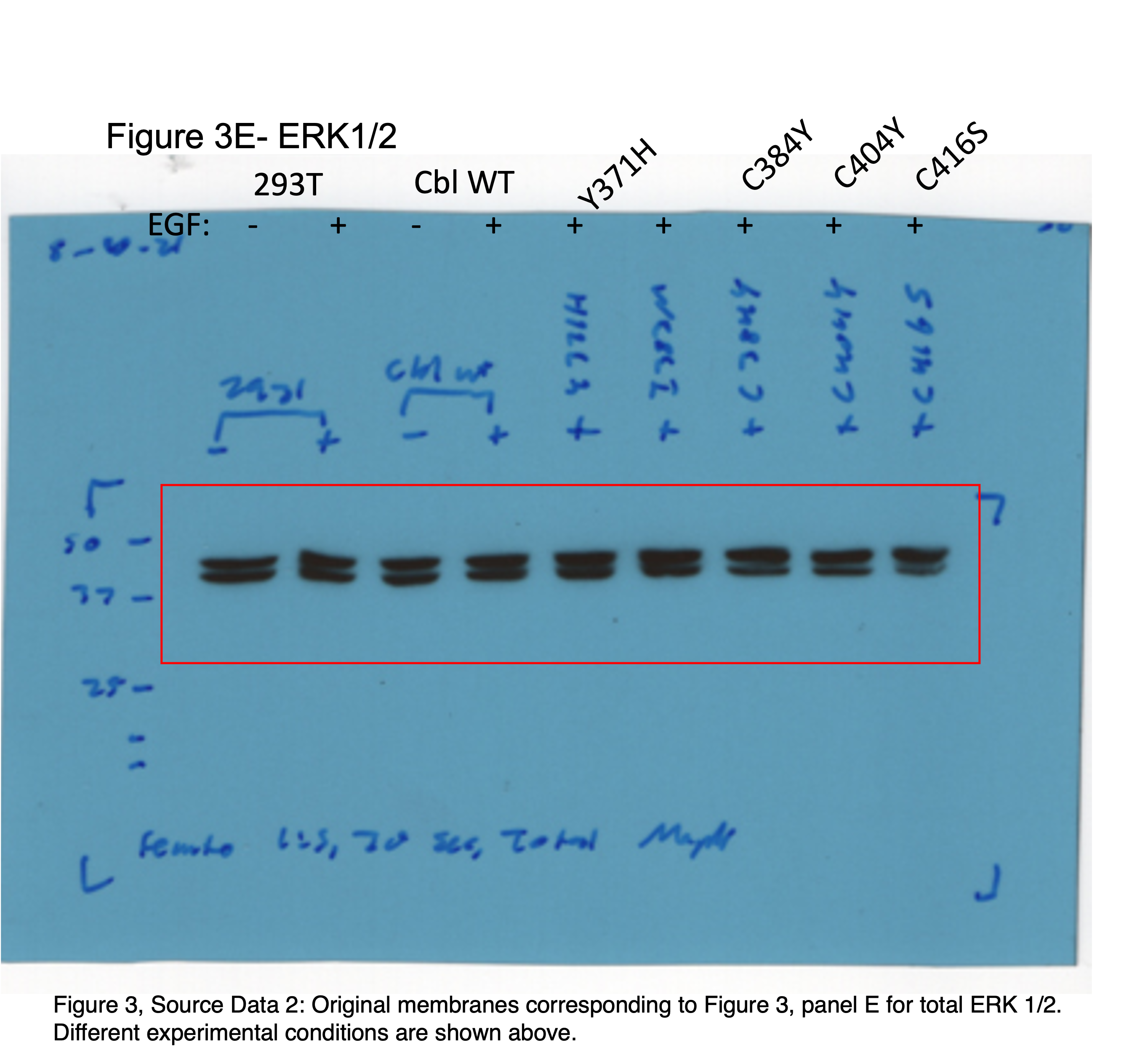

Supplement: Figure 3—source data 2. [file elife-96519-fig3-data2.zip › Figure 3E- ERK1:2 .png]

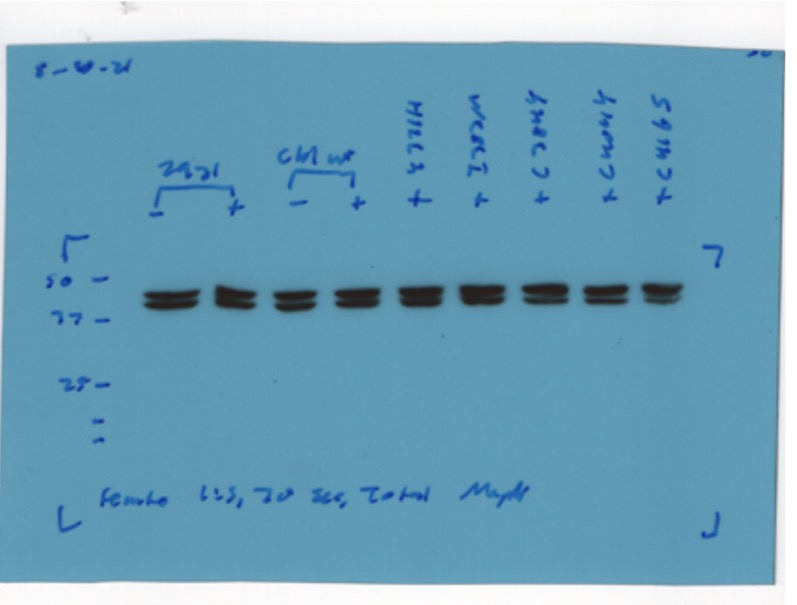

Supplement: Figure 3—source data 3. [file elife-96519-fig3-data3.zip › ERK1:2 3E.jpg]

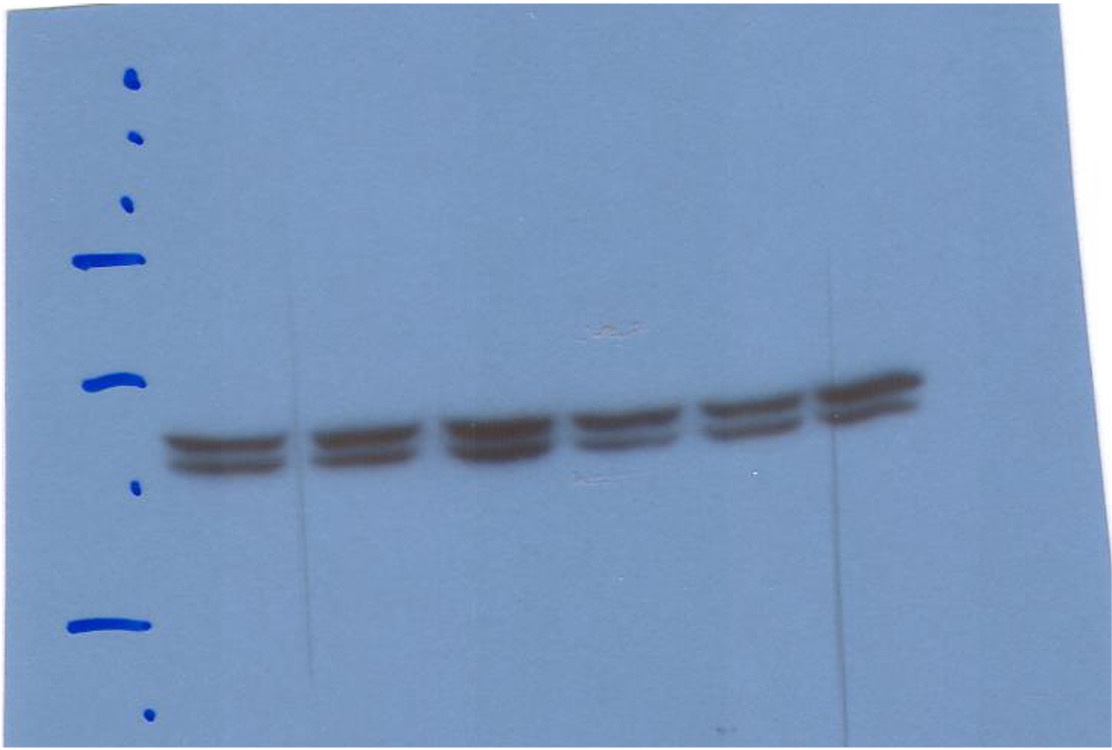

Supplement: Figure 3—source data 3. [file elife-96519-fig3-data3.zip › ERK1:2 3F.jpg]

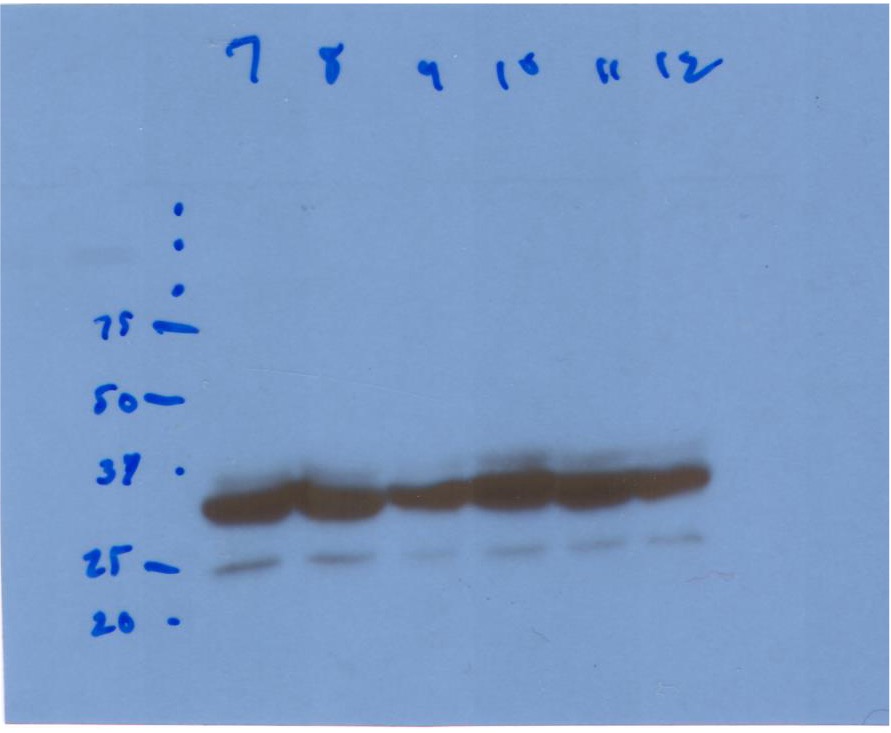

Supplement: Figure 3—source data 3. [file elife-96519-fig3-data3.zip › FLAG RIT1 3F.jpg]

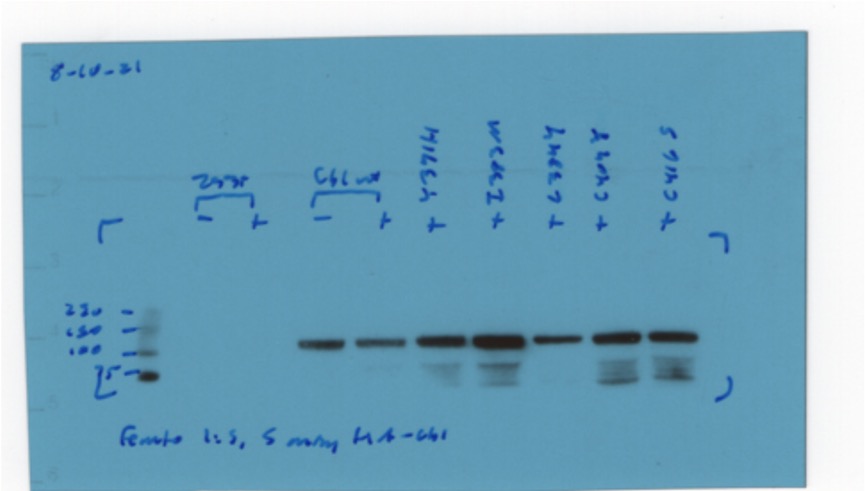

Supplement: Figure 3—source data 3. [file elife-96519-fig3-data3.zip › HA-Cbl 3E.jpg]

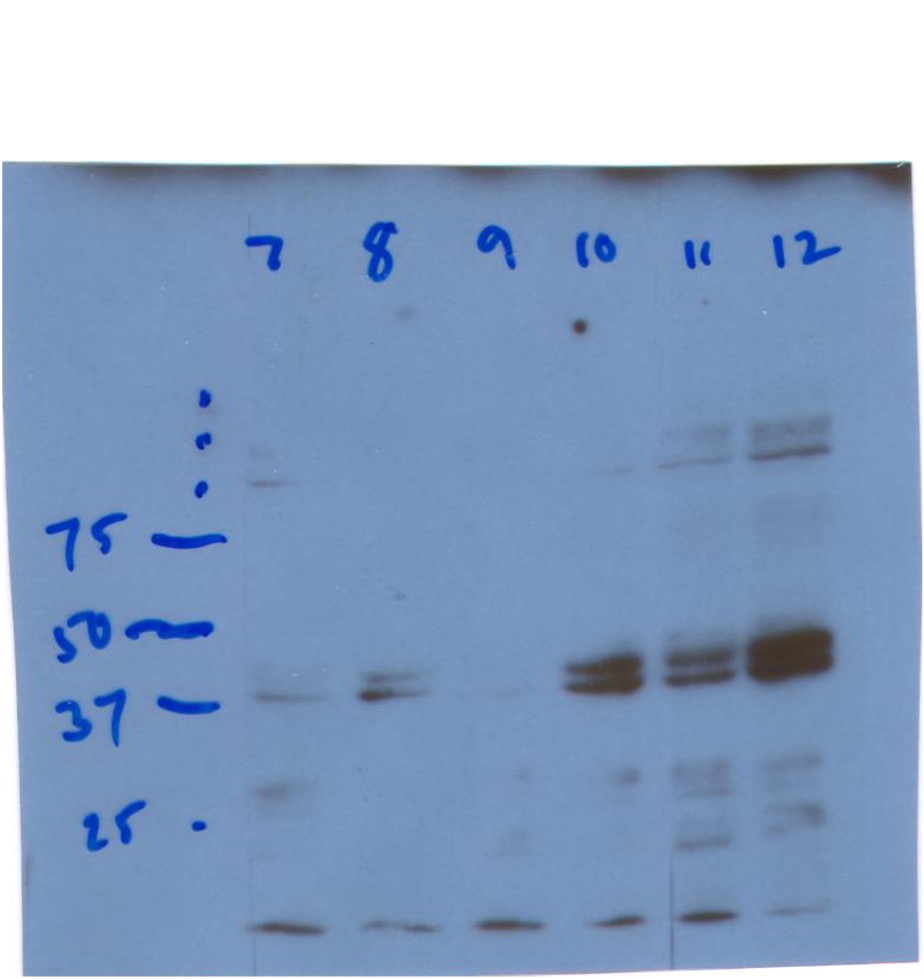

Supplement: Figure 3—source data 3. [file elife-96519-fig3-data3.zip › pERK 3F.jpg]

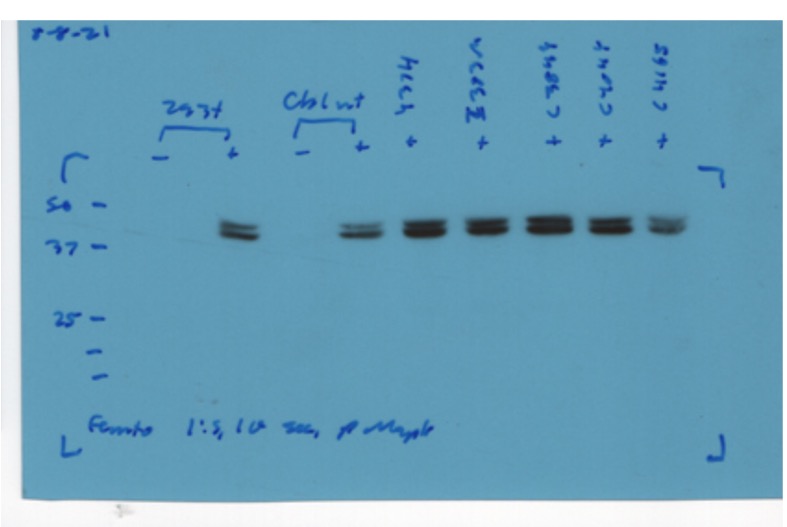

Supplement: Figure 3—source data 3. [file elife-96519-fig3-data3.zip › pERK1:2 3E.jpg]

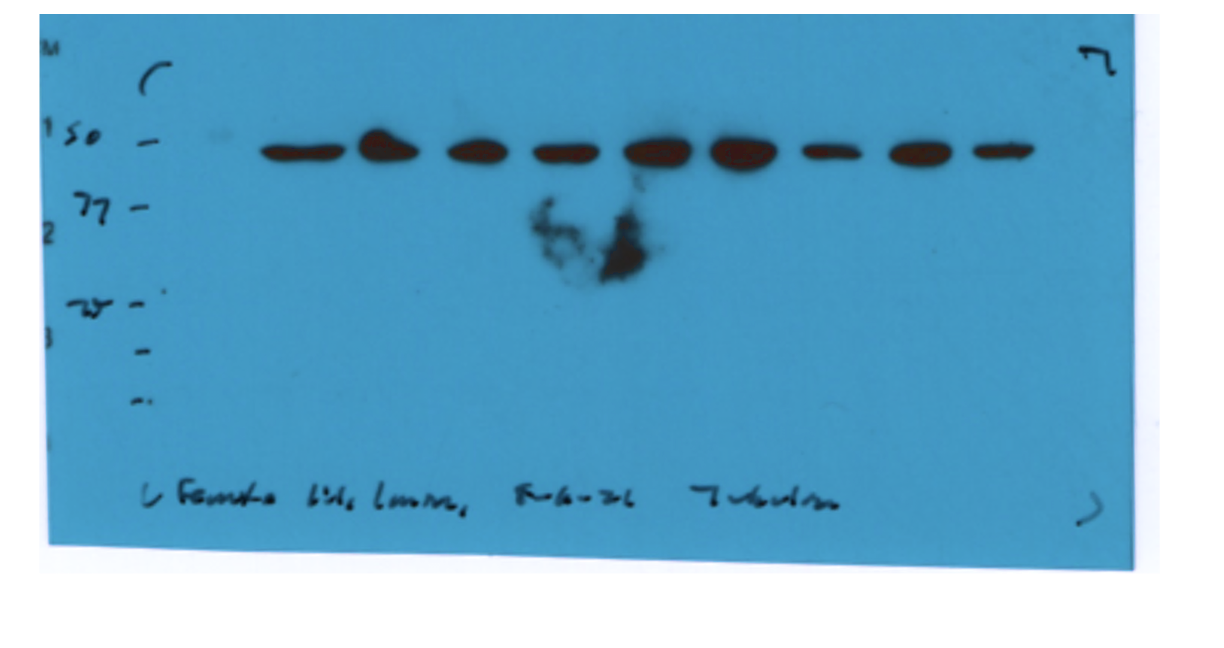

Supplement: Figure 3—source data 3. [file elife-96519-fig3-data3.zip › Tubulin 3E.jpg]

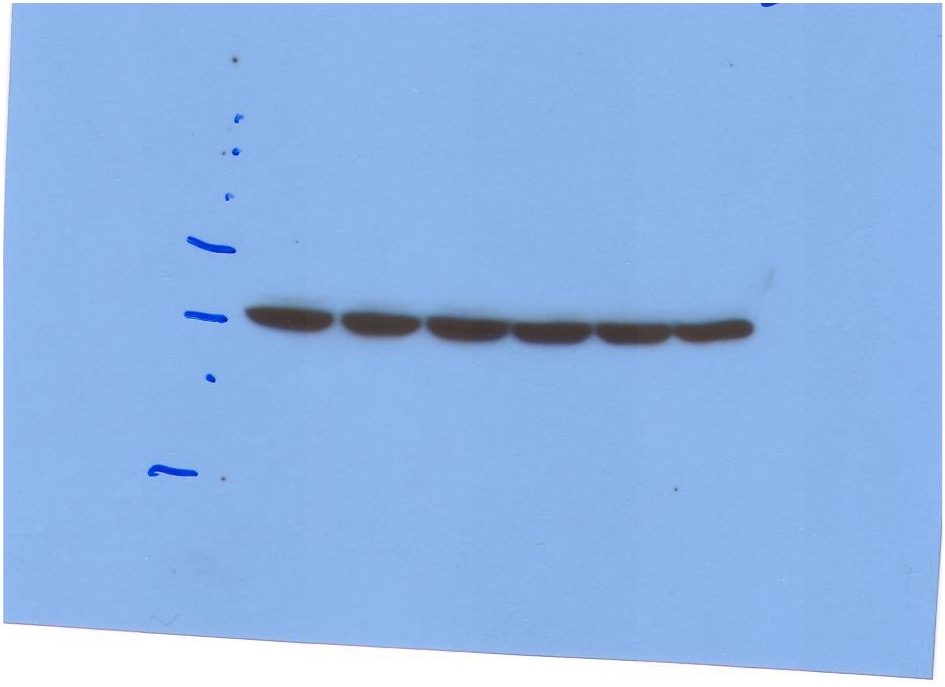

Supplement: Figure 3—source data 3. [file elife-96519-fig3-data3.zip › Tubulin 3F.jpg]

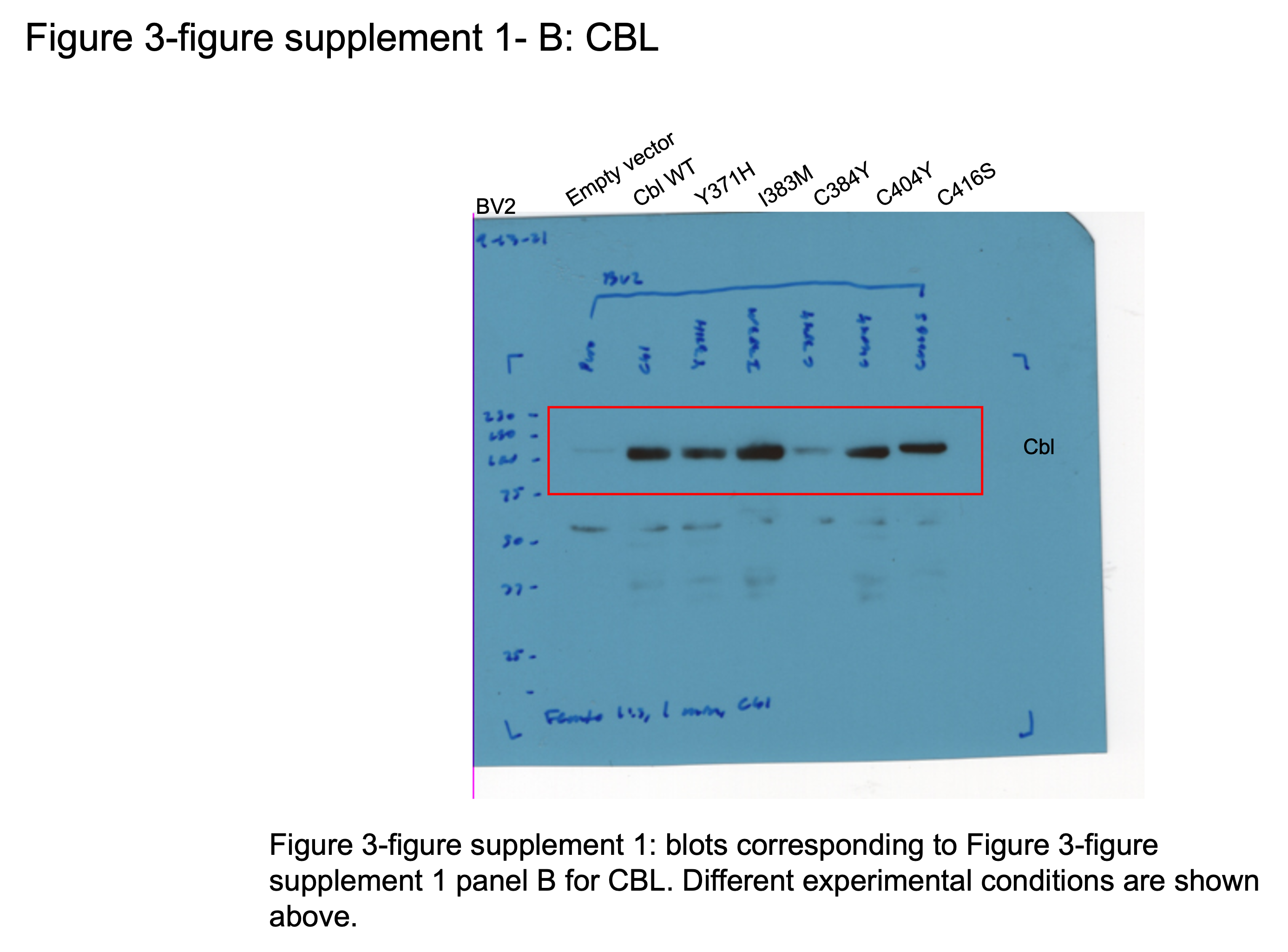

Supplement: Figure 3—figure supplement 1—source data 2. [file elife-96519-fig3-figsupp1-data2.zip › CBLFigure 3-figure supplement 1- B.png]

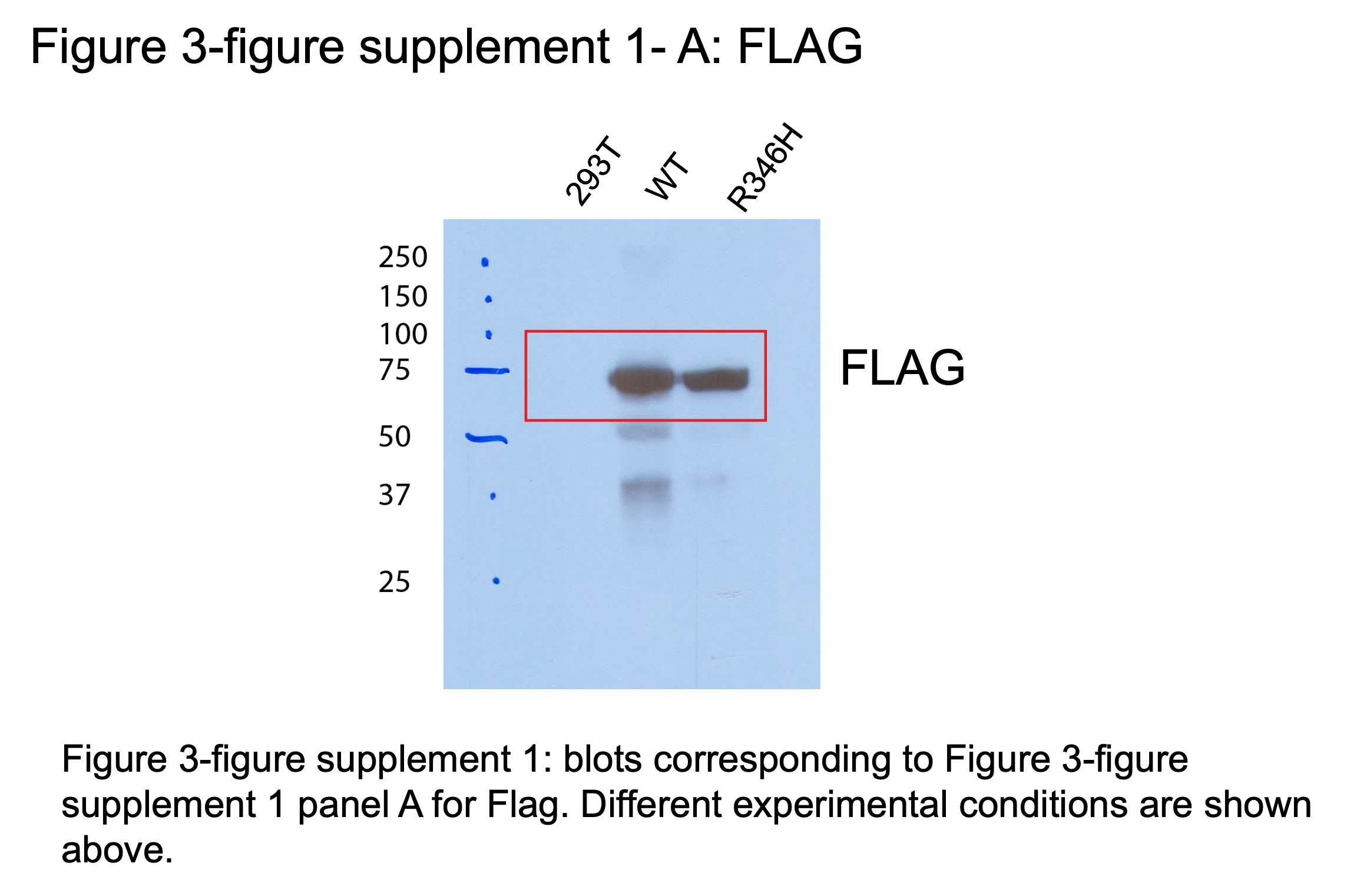

Supplement: Figure 3—figure supplement 1—source data 2. [file elife-96519-fig3-figsupp1-data2.zip › FLAG Figure 3-figure supplement 1- A.png]

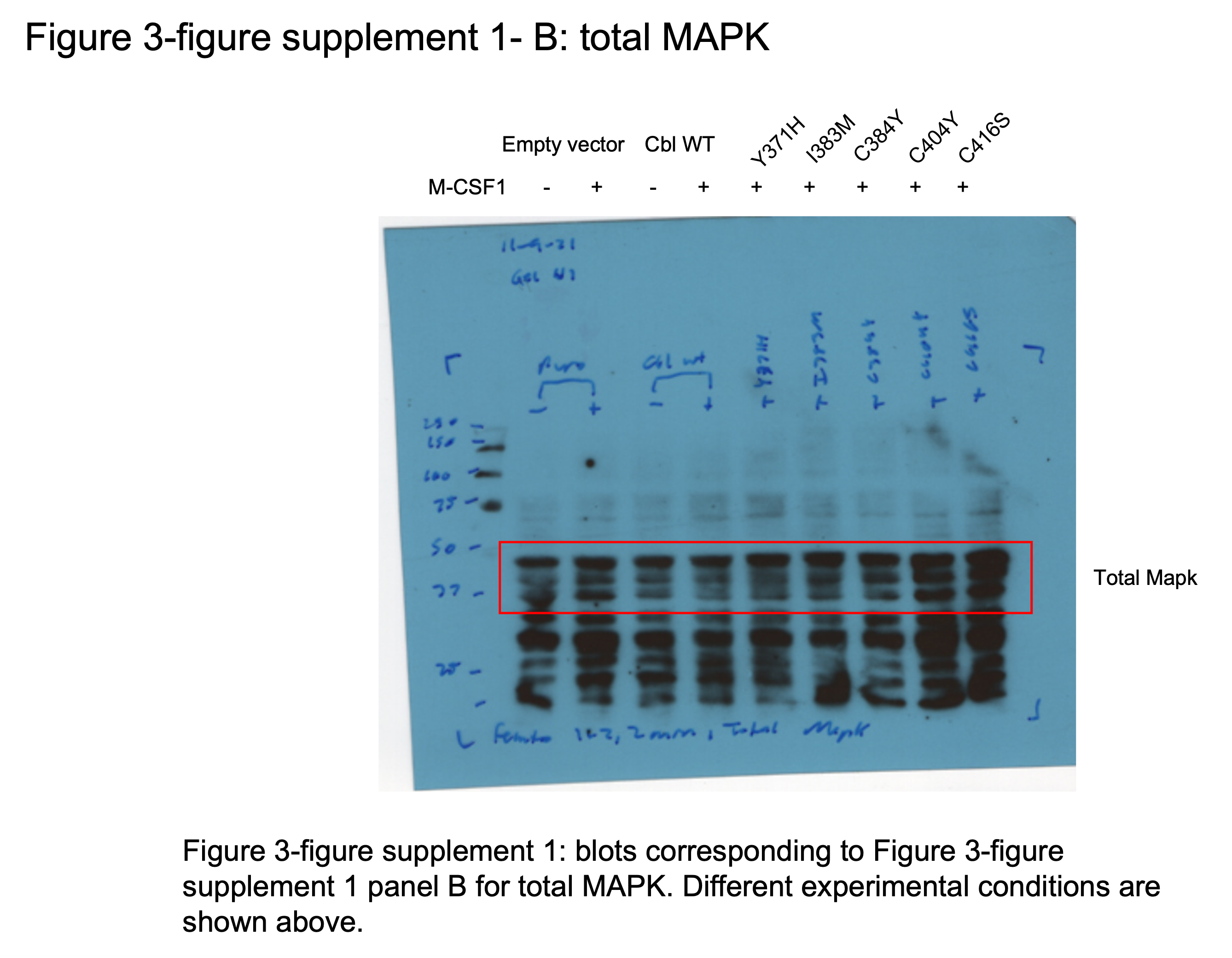

Supplement: Figure 3—figure supplement 1—source data 2. [file elife-96519-fig3-figsupp1-data2.zip › MAPK Figure 3-figure supplement 1- B.png]

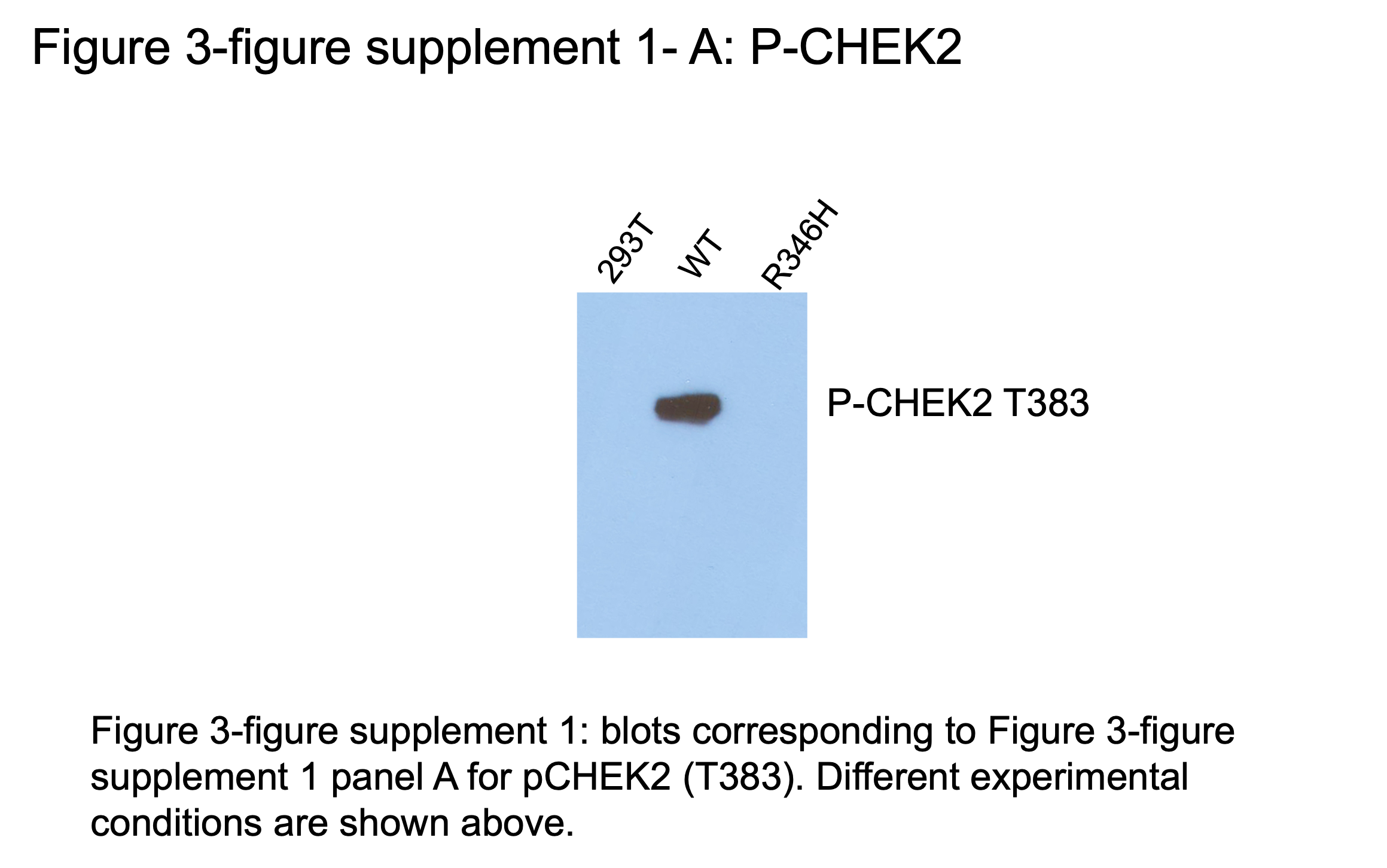

Supplement: Figure 3—figure supplement 1—source data 2. [file elife-96519-fig3-figsupp1-data2.zip › pCHEK2 Figure 3-figure supplement 1- A.png]

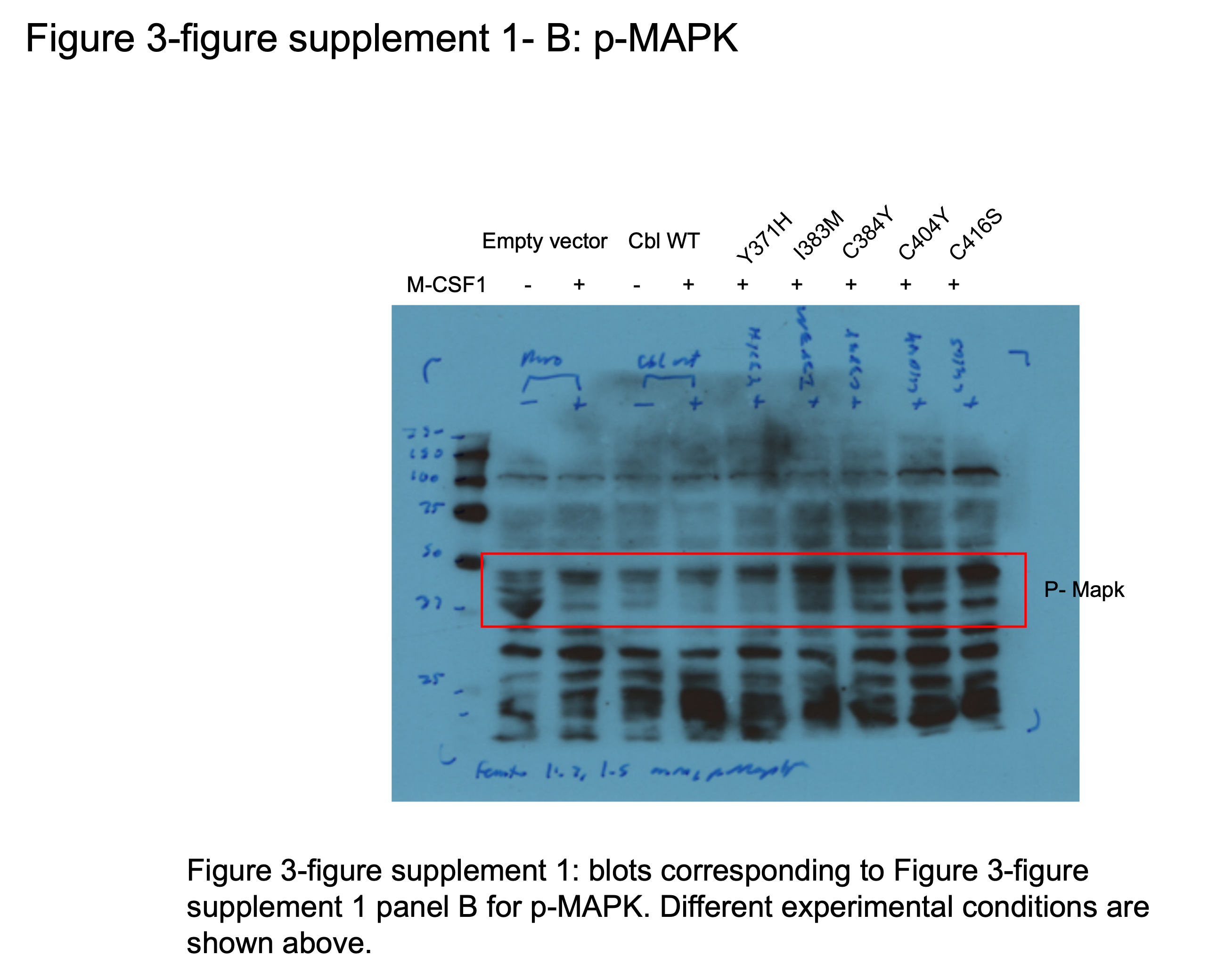

Supplement: Figure 3—figure supplement 1—source data 2. [file elife-96519-fig3-figsupp1-data2.zip › pMAPK Figure 3-figure supplement 1- B.png]

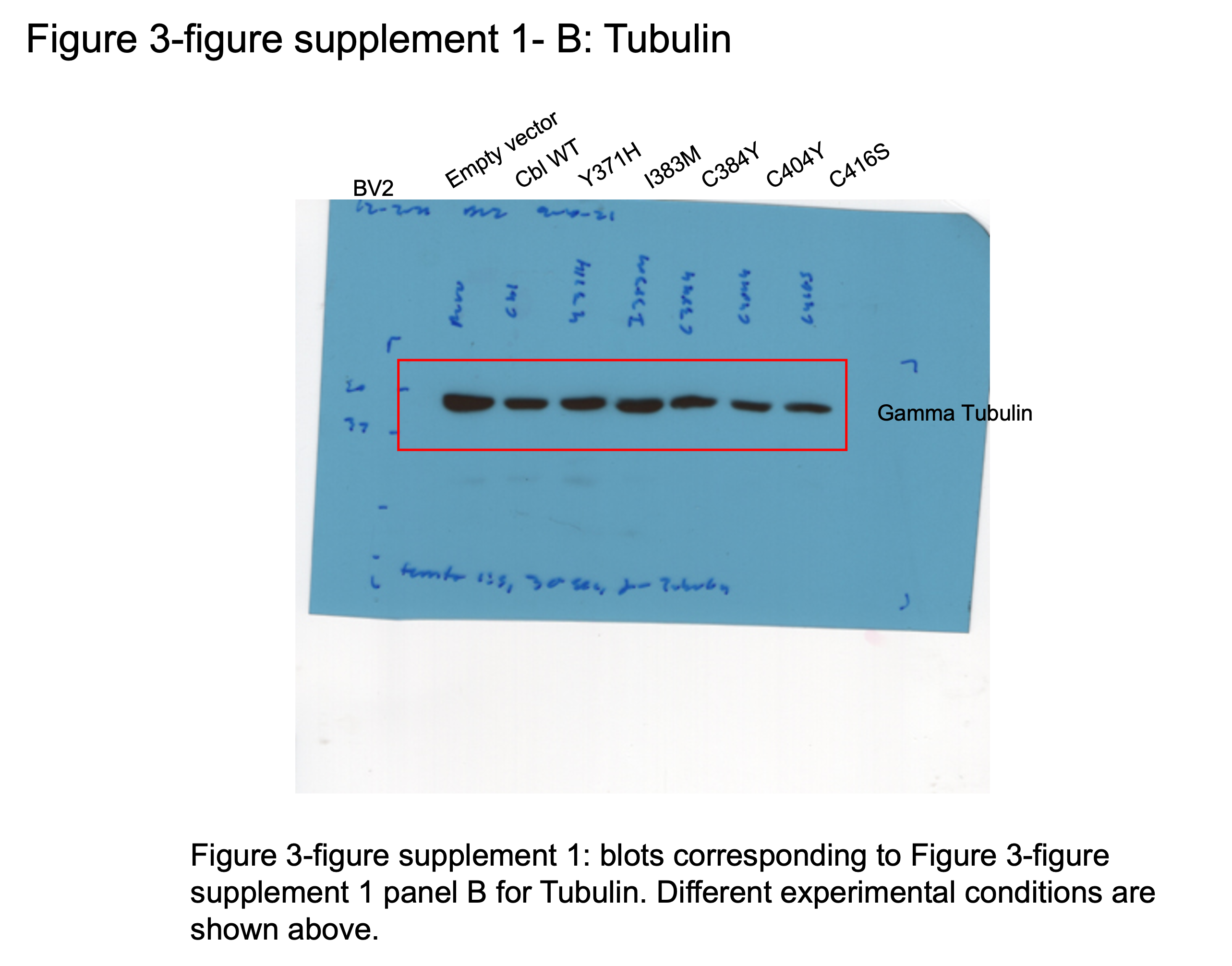

Supplement: Figure 3—figure supplement 1—source data 2. [file elife-96519-fig3-figsupp1-data2.zip › tubulin Figure 3-figure supplement 1- B.png]

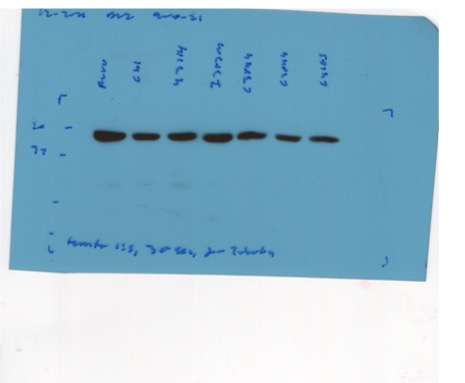

Supplement: Figure 3—figure supplement 1—source data 3. [file elife-96519-fig3-figsupp1-data3.zip › Tubulin Figure 3-figure supplement 1 B.jpg]

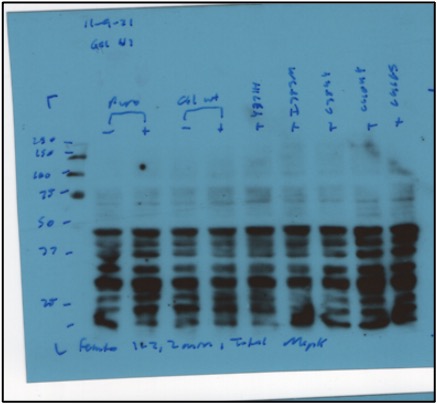

Supplement: Figure 3—figure supplement 1—source data 3. [file elife-96519-fig3-figsupp1-data3.zip › Total MAPK Figure 3-figure supplement 1 B.jpg]

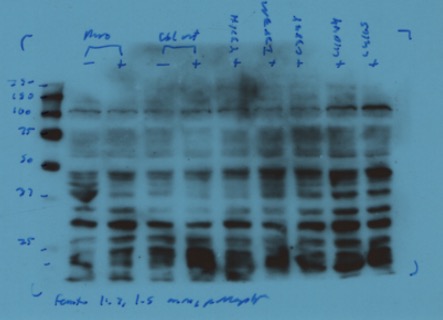

Supplement: Figure 3—figure supplement 1—source data 3. [file elife-96519-fig3-figsupp1-data3.zip › pMAPK Figure 3-figure supplement 1 B.jpg]

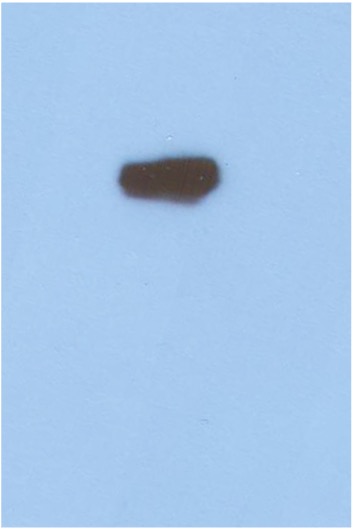

Supplement: Figure 3—figure supplement 1—source data 3. [file elife-96519-fig3-figsupp1-data3.zip › P-CHEK2 Figure 3-figure supplement 1 A .jpg]

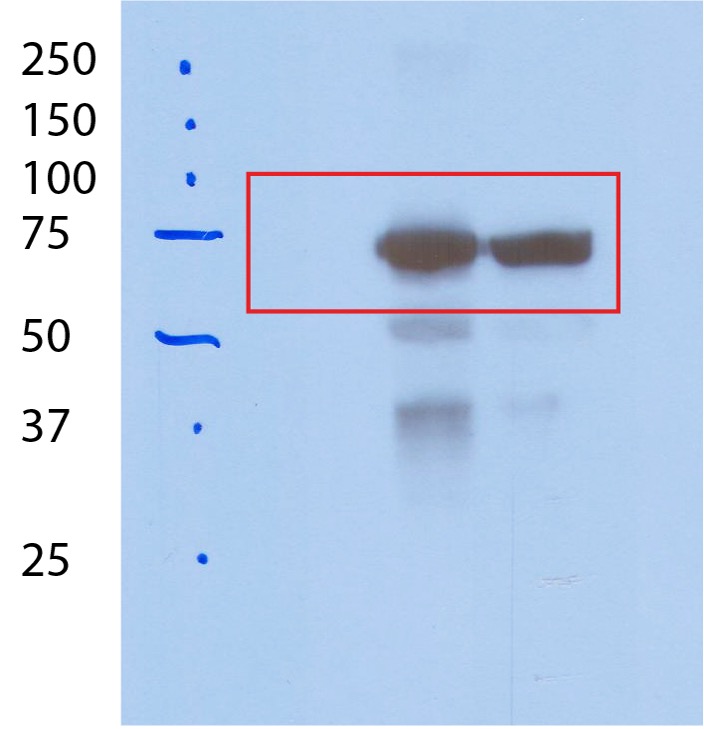

Supplement: Figure 3—figure supplement 1—source data 3. [file elife-96519-fig3-figsupp1-data3.zip › Flag Figure 3-figure supplement 1 A.jpg]

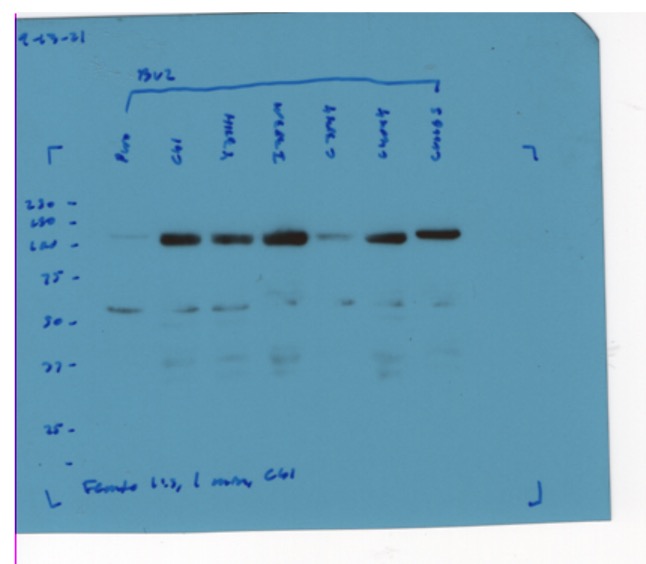

Supplement: Figure 3—figure supplement 1—source data 3. [file elife-96519-fig3-figsupp1-data3.zip › CBL Figure 3-figure supplement 1 B.jpg]

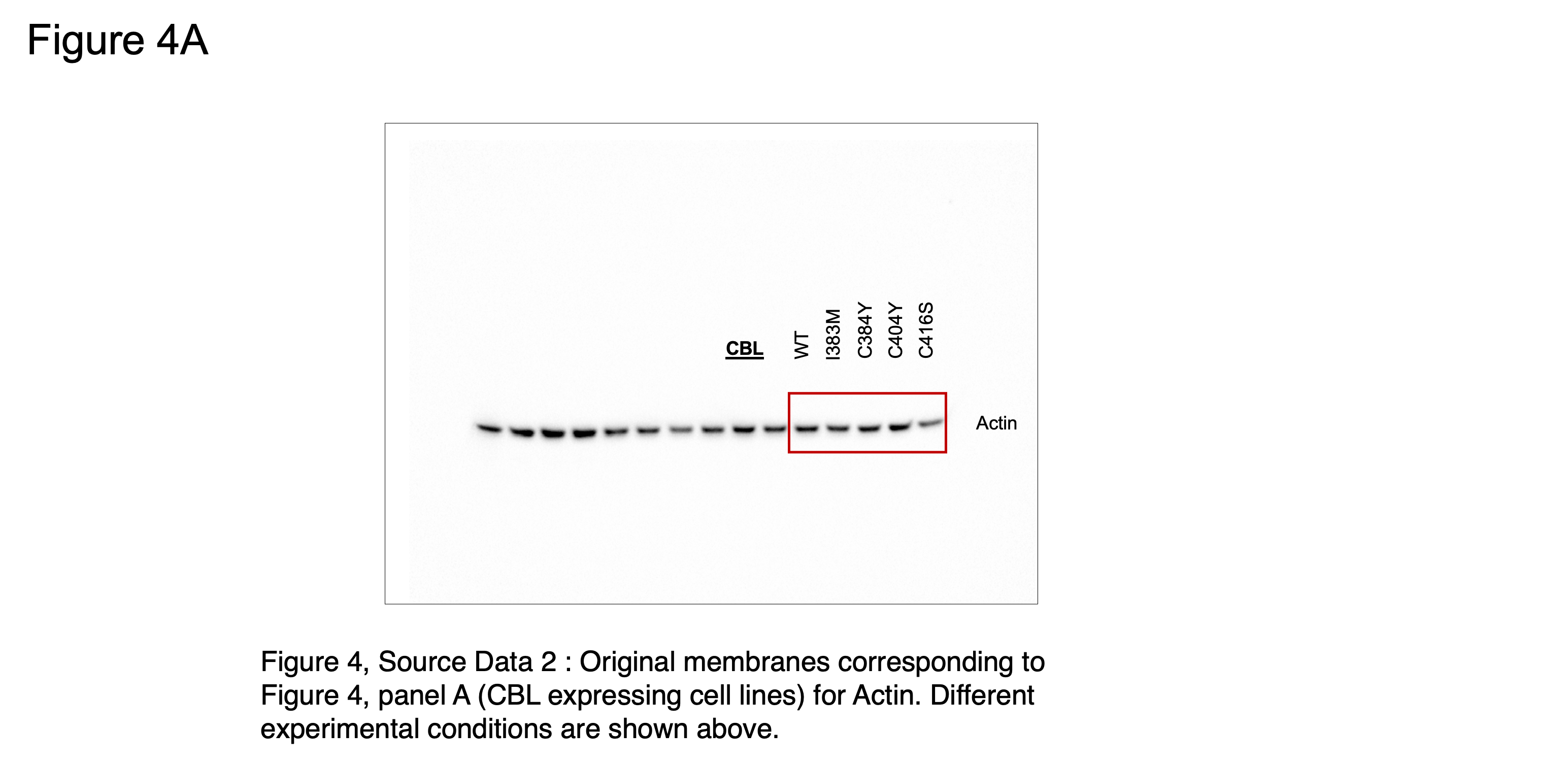

Supplement: Figure 4—source data 2. [file elife-96519-fig4-data2.zip › Actin- cbl 4A.png]

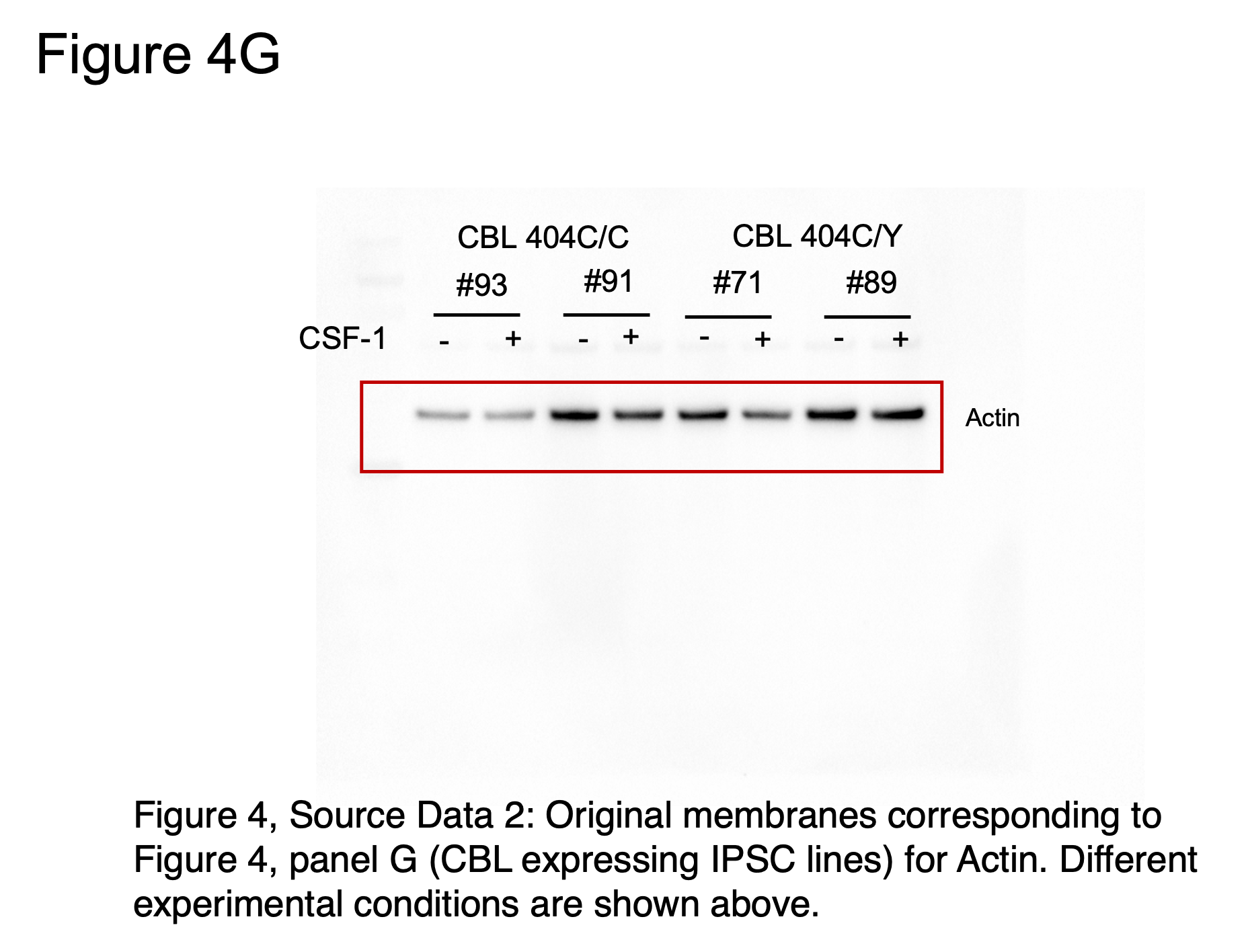

Supplement: Figure 4—source data 2. [file elife-96519-fig4-data2.zip › Actin-CBL IPSC-4G.png]

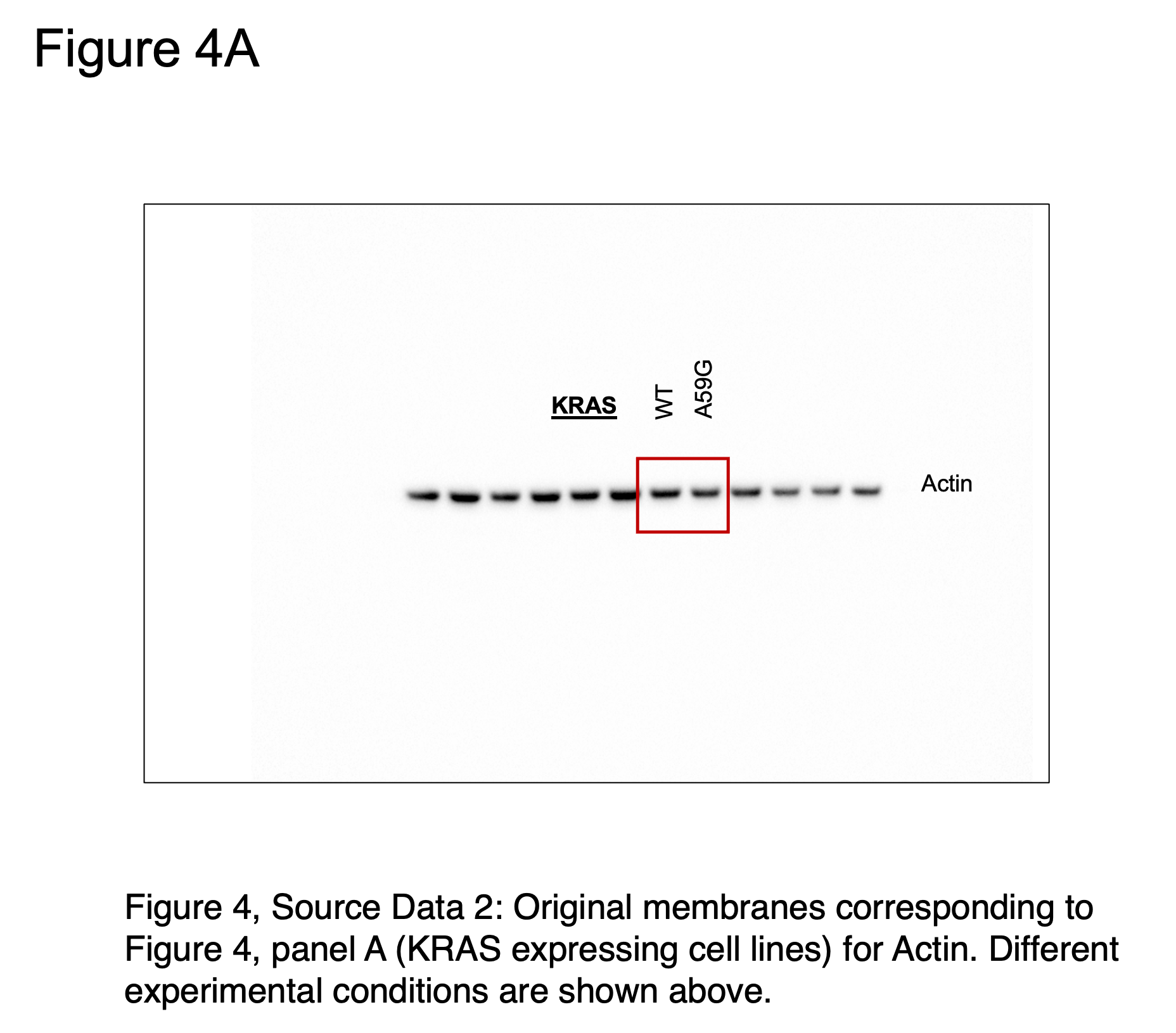

Supplement: Figure 4—source data 2. [file elife-96519-fig4-data2.zip › Actin-KRAS-4A.png]

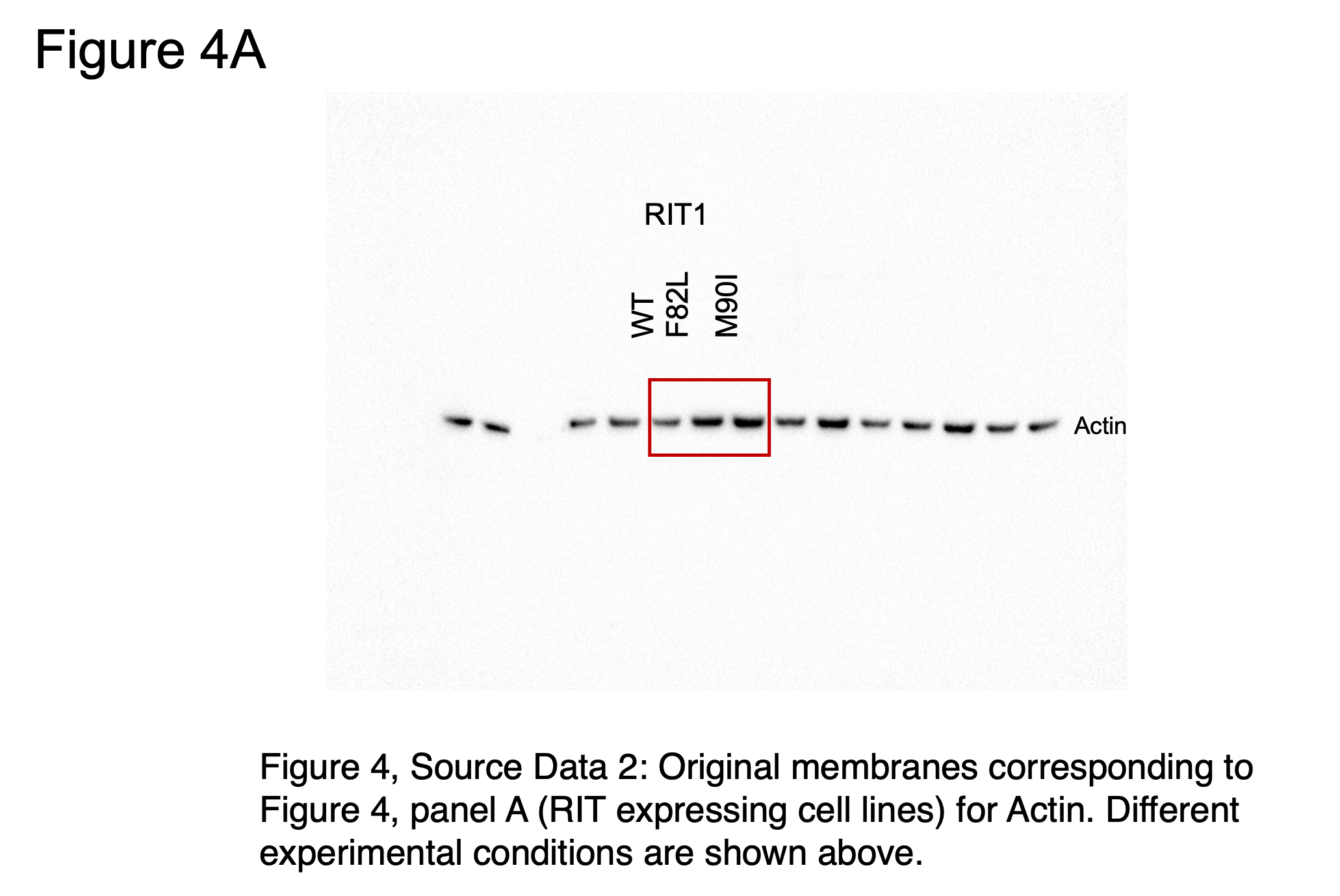

Supplement: Figure 4—source data 2. [file elife-96519-fig4-data2.zip › Actin-rit1-4A.png]

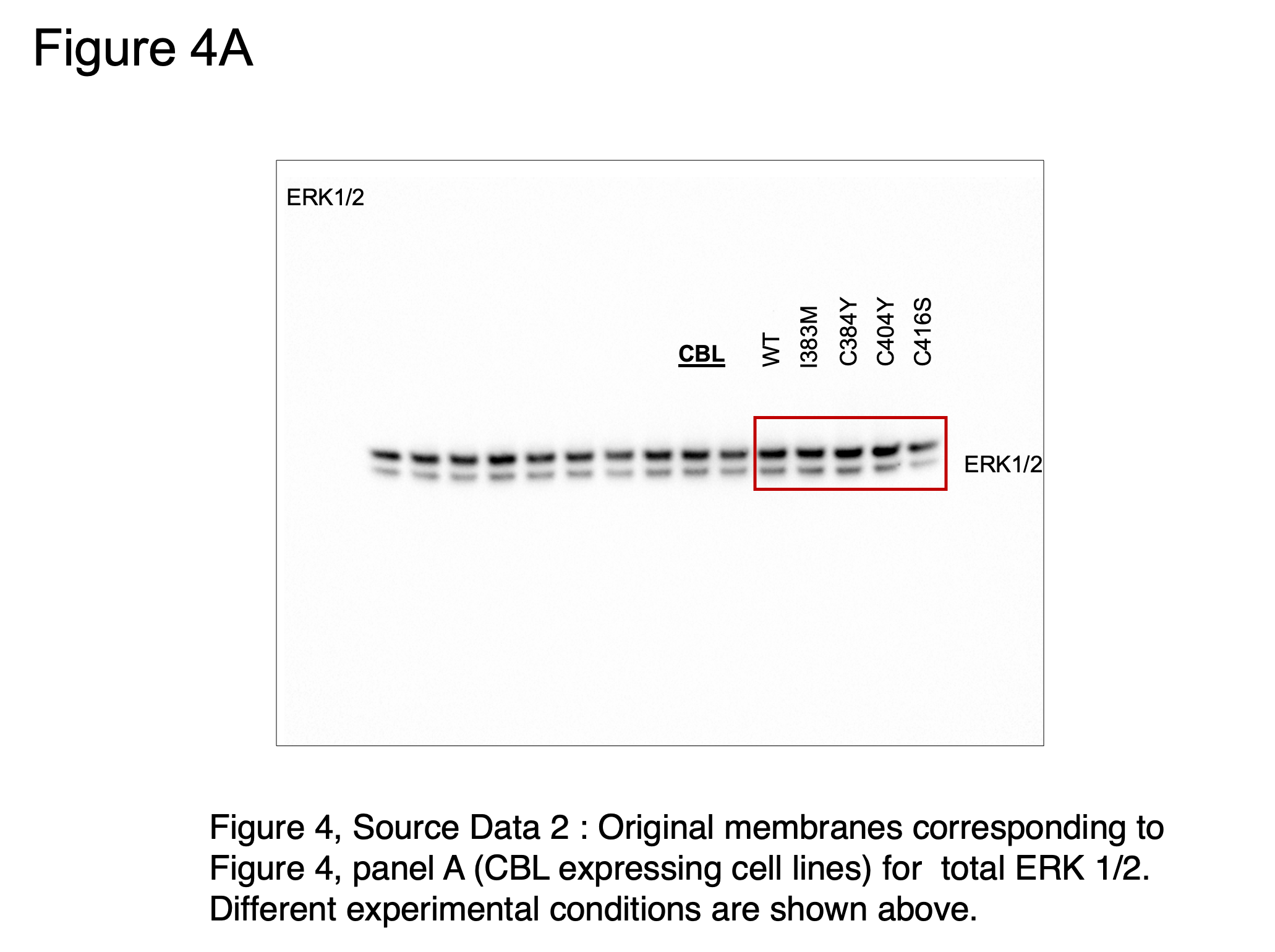

Supplement: Figure 4—source data 2. [file elife-96519-fig4-data2.zip › ERK-cbl 4A.png]

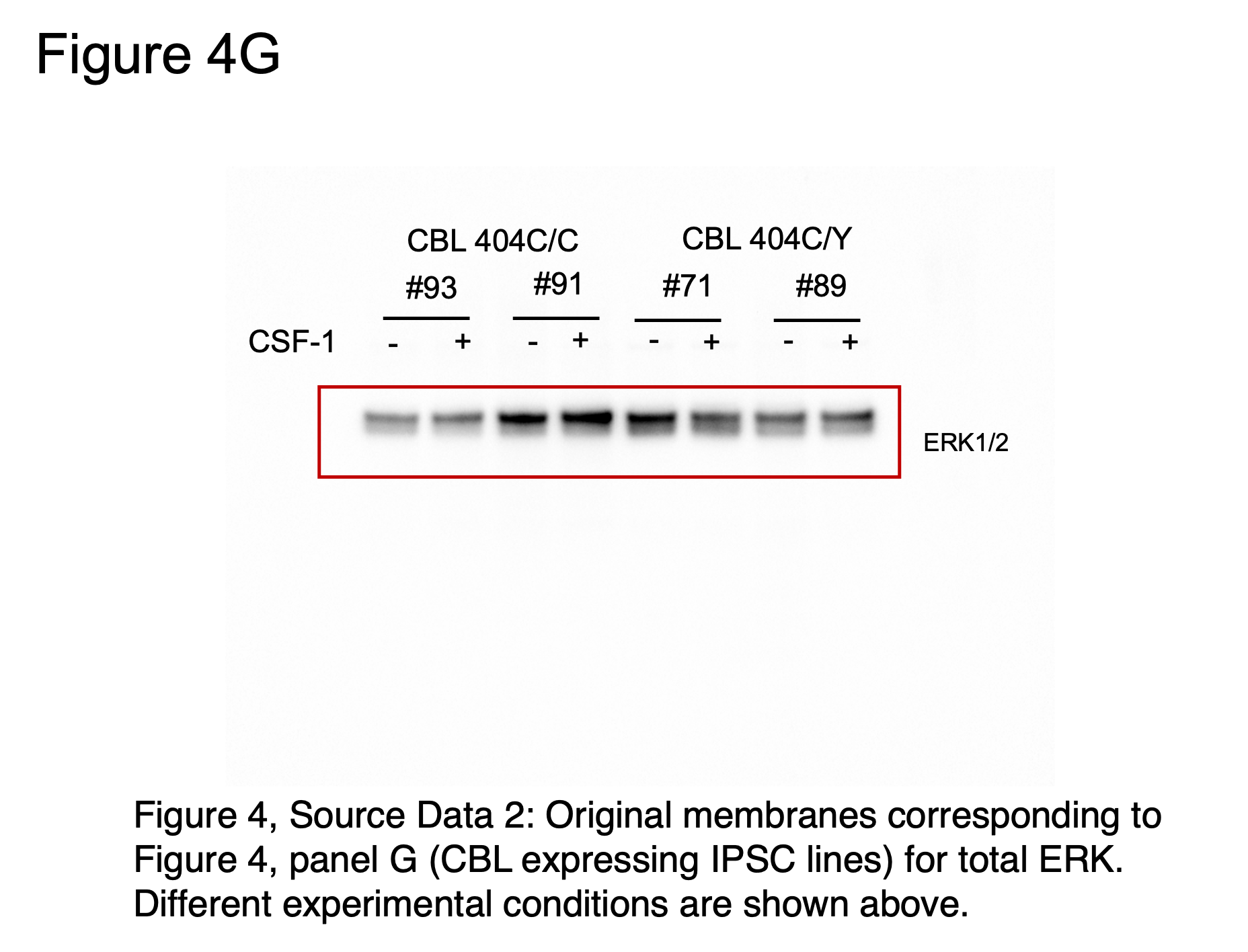

Supplement: Figure 4—source data 2. [file elife-96519-fig4-data2.zip › ERK-CBL IPSC-4G.png]

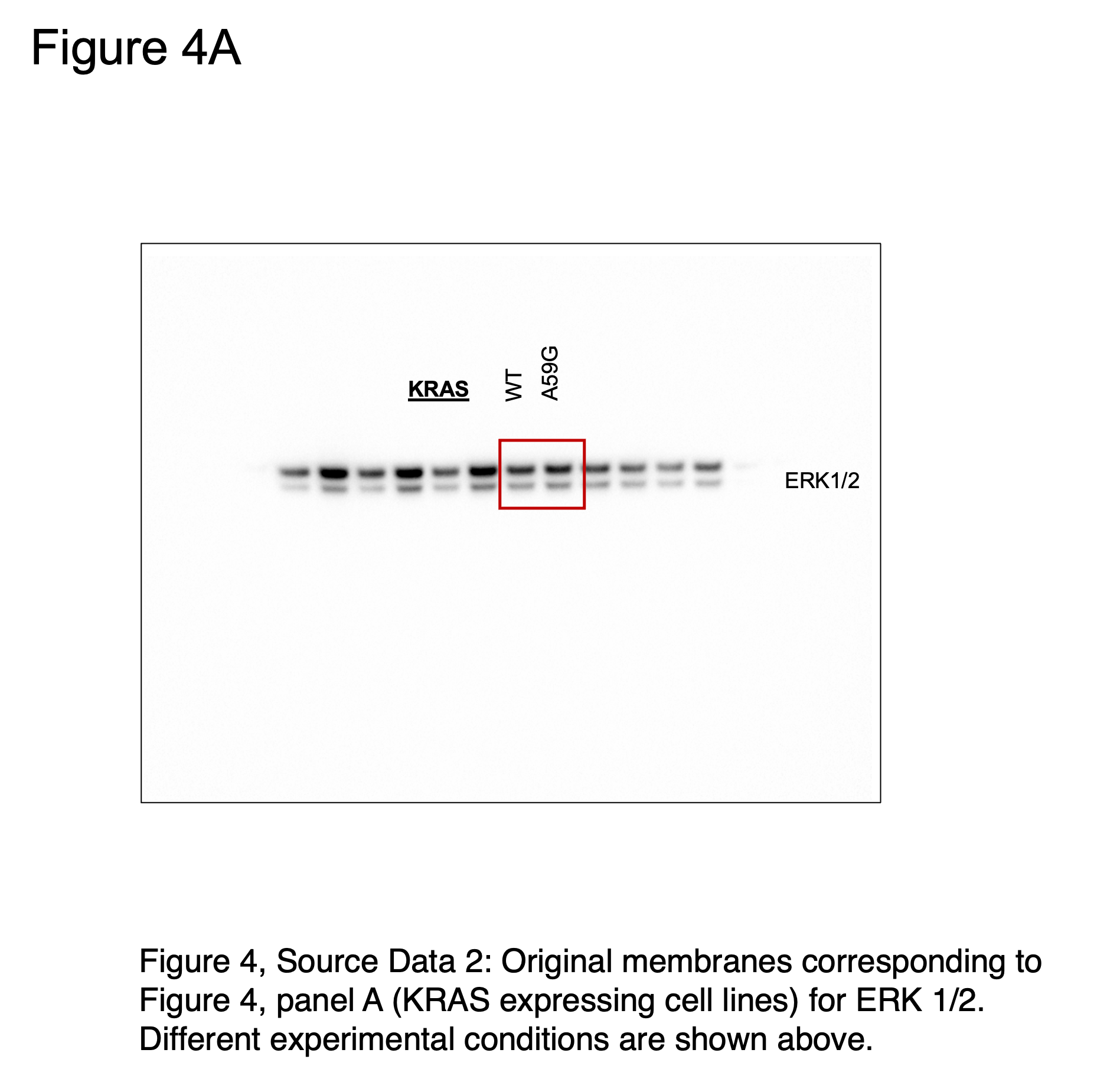

Supplement: Figure 4—source data 2. [file elife-96519-fig4-data2.zip › ERK-KRAS-4A.png]

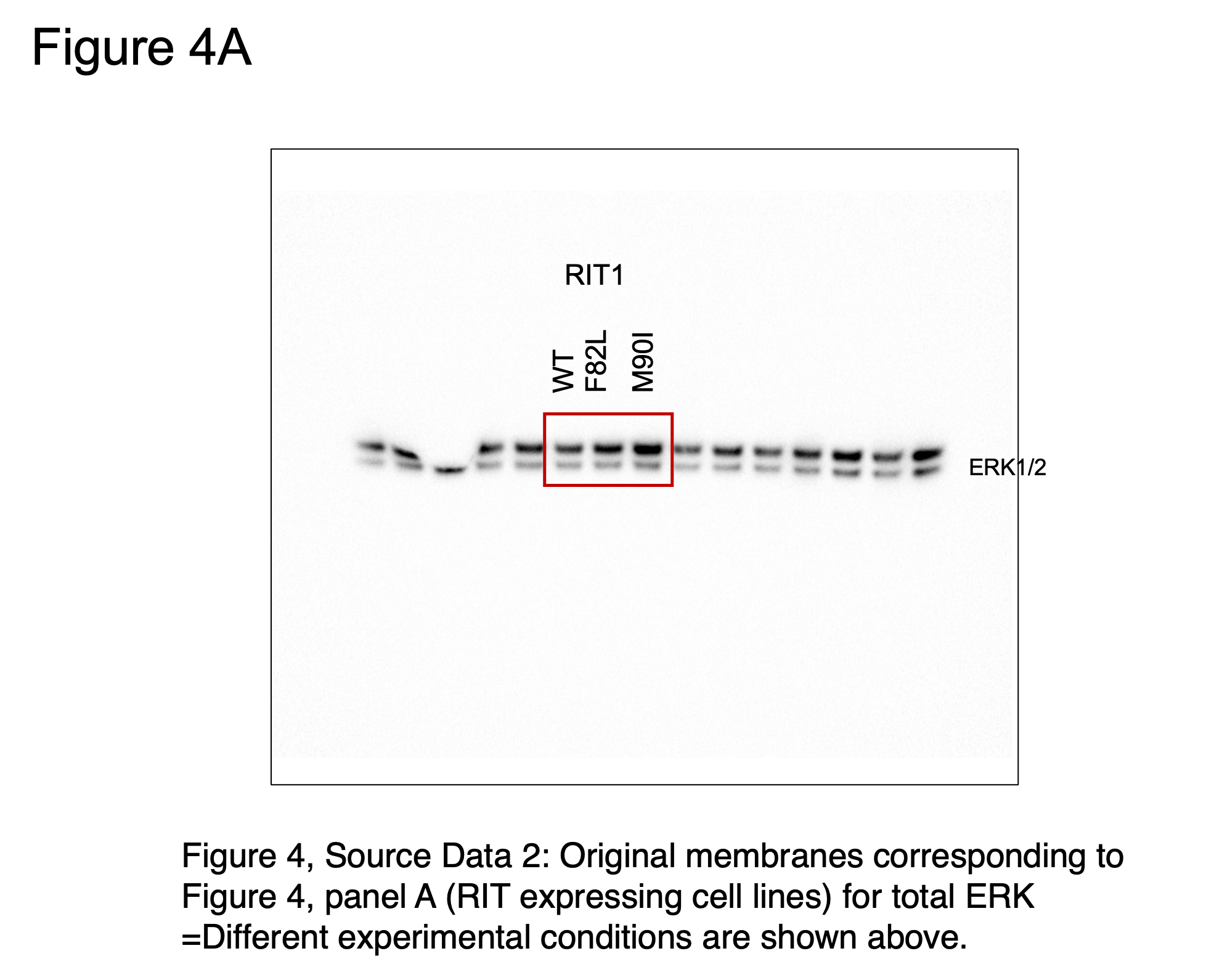

Supplement: Figure 4—source data 2. [file elife-96519-fig4-data2.zip › ERK-Rit1-4A.png]

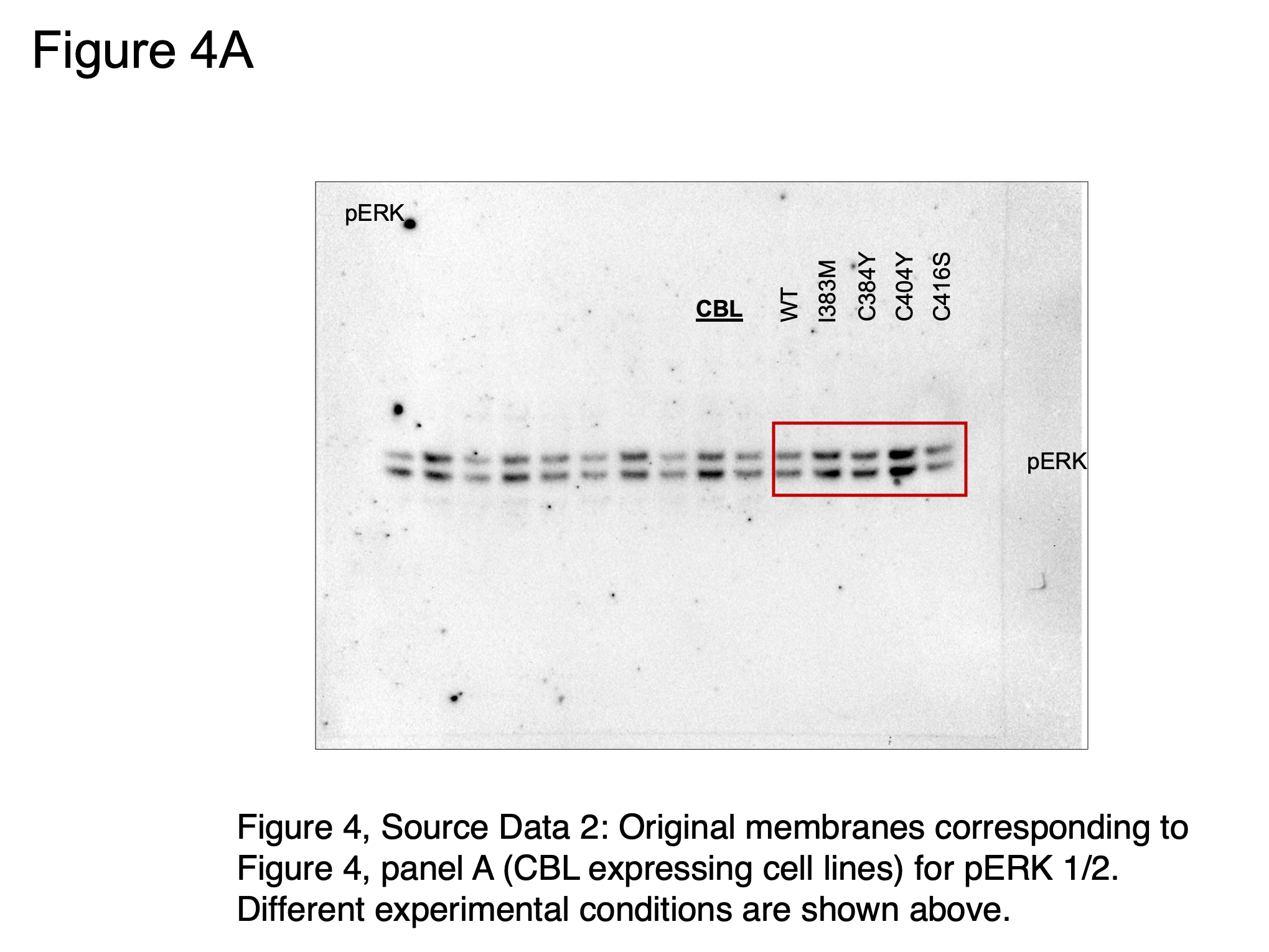

Supplement: Figure 4—source data 2. [file elife-96519-fig4-data2.zip › pERK-cbl 4A.png]

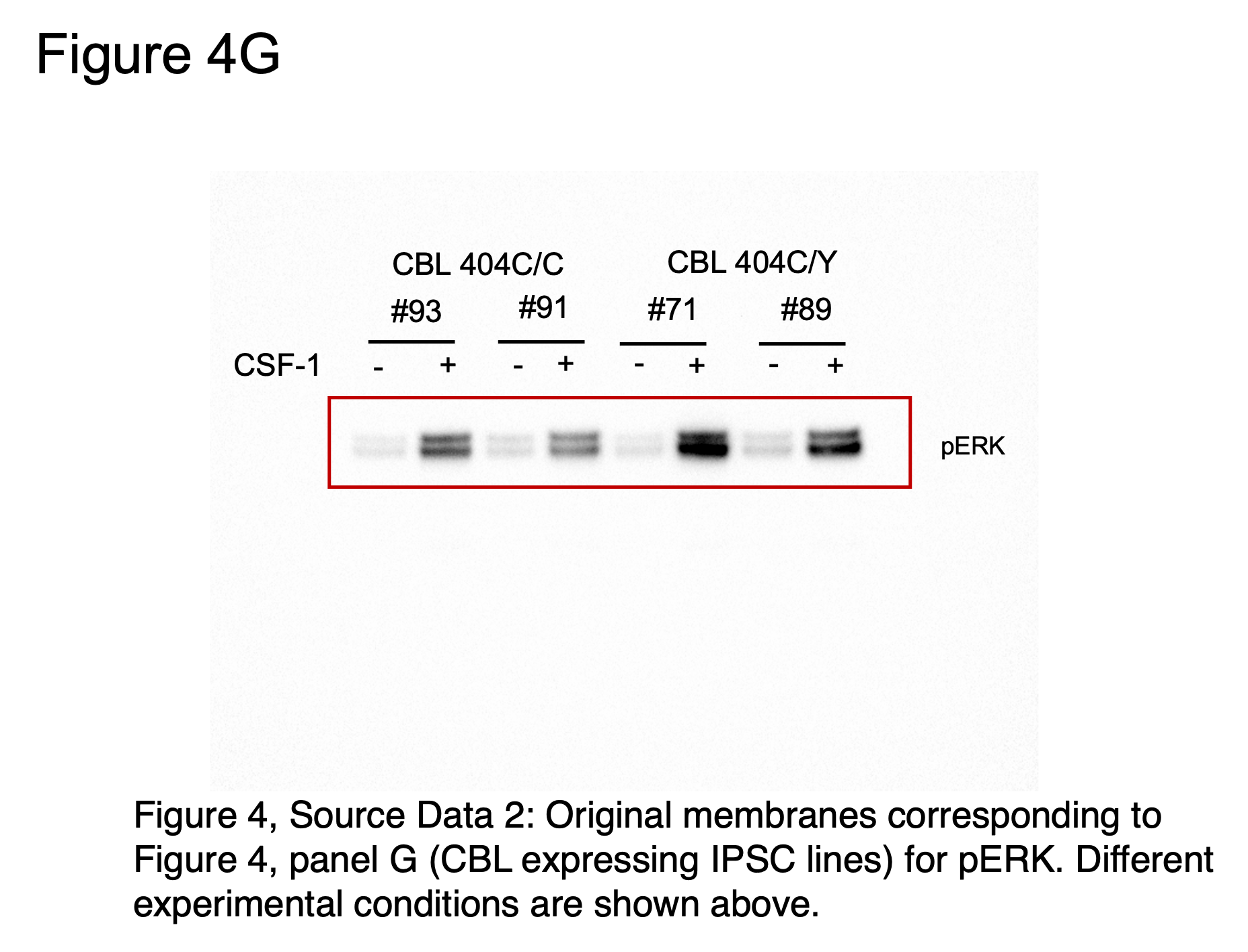

Supplement: Figure 4—source data 2. [file elife-96519-fig4-data2.zip › pERK-CBL IPSC-4G.png]

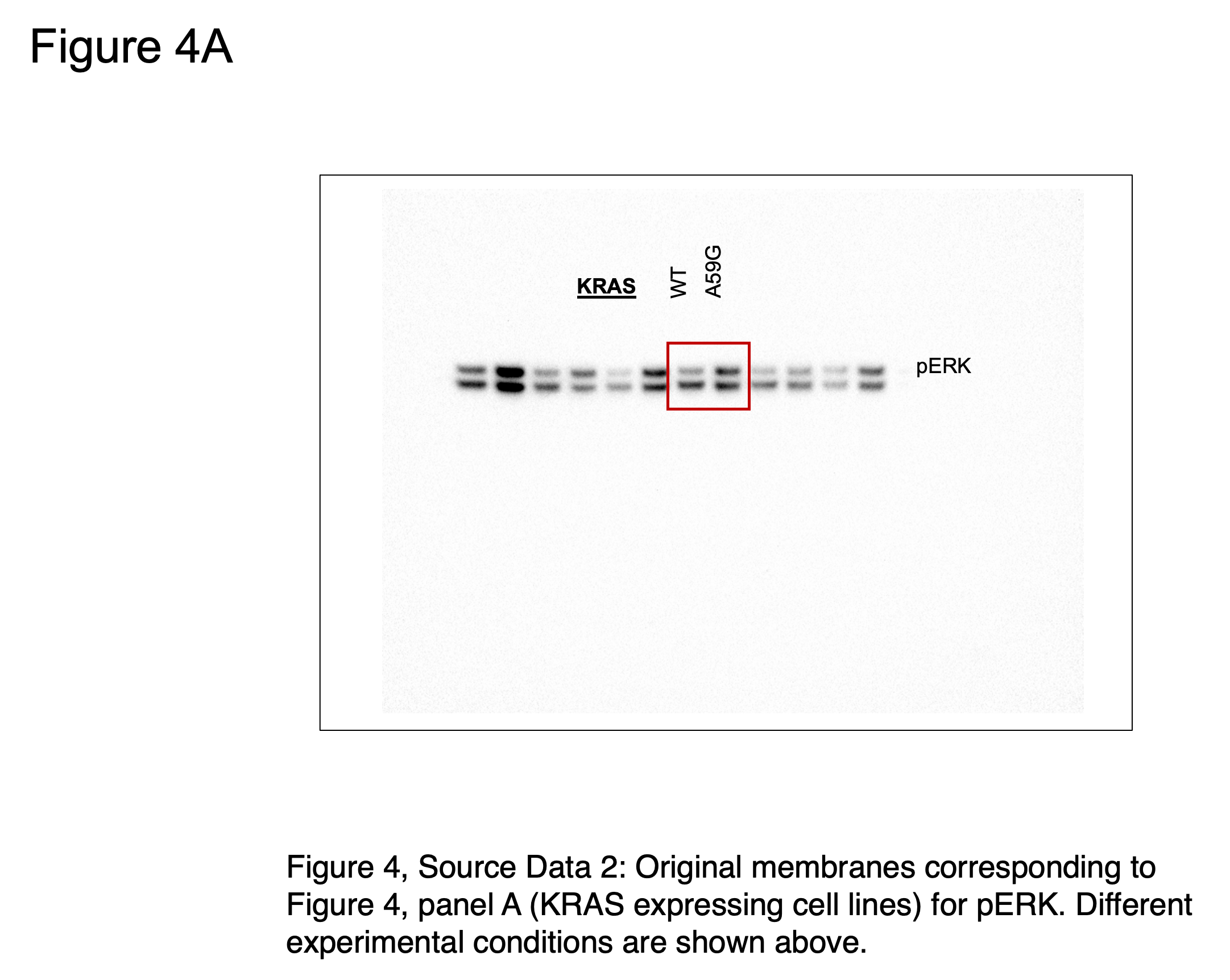

Supplement: Figure 4—source data 2. [file elife-96519-fig4-data2.zip › pERK-KRAS-4A.png]

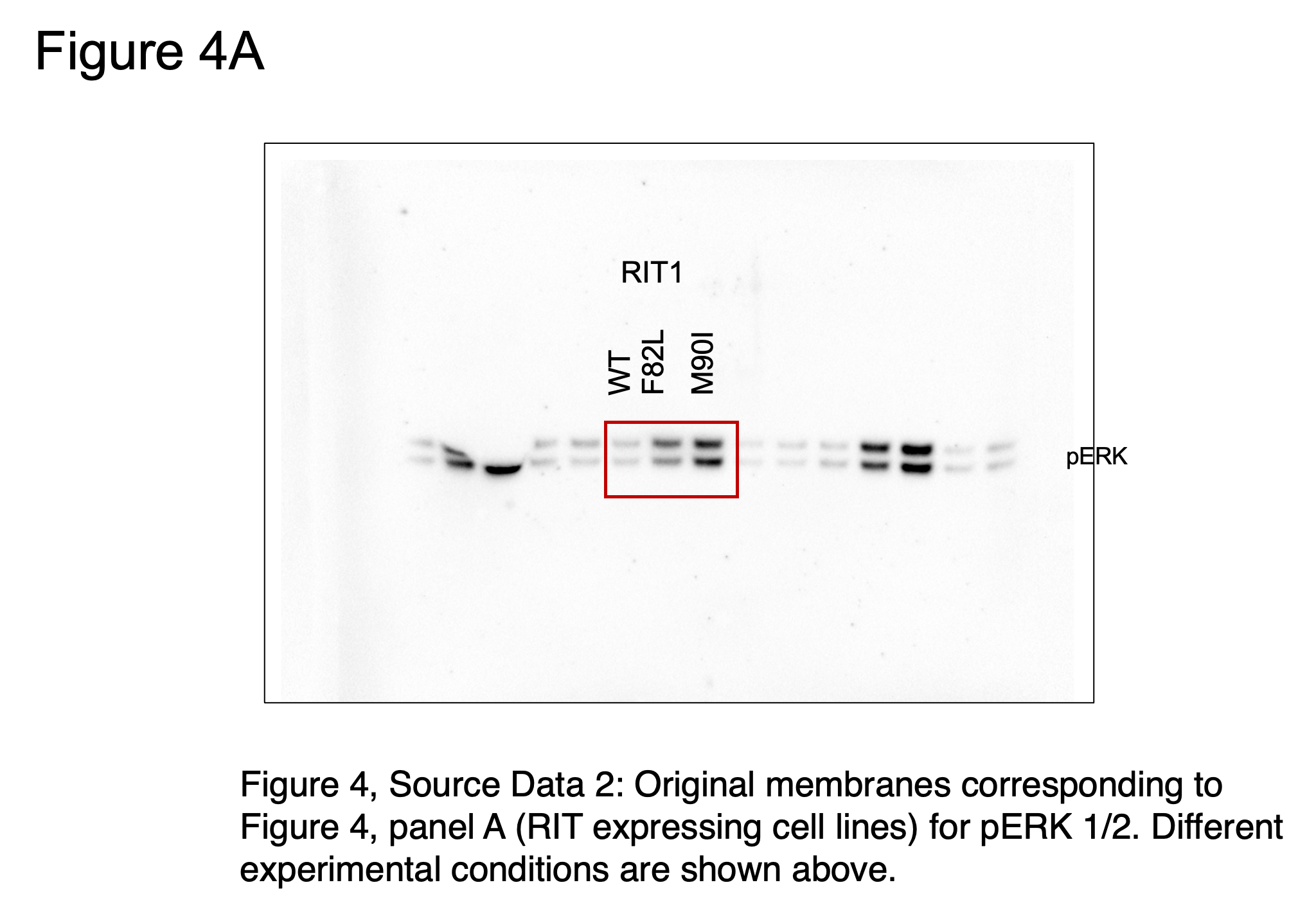

Supplement: Figure 4—source data 2. [file elife-96519-fig4-data2.zip › pERK-rit1-4A.png]

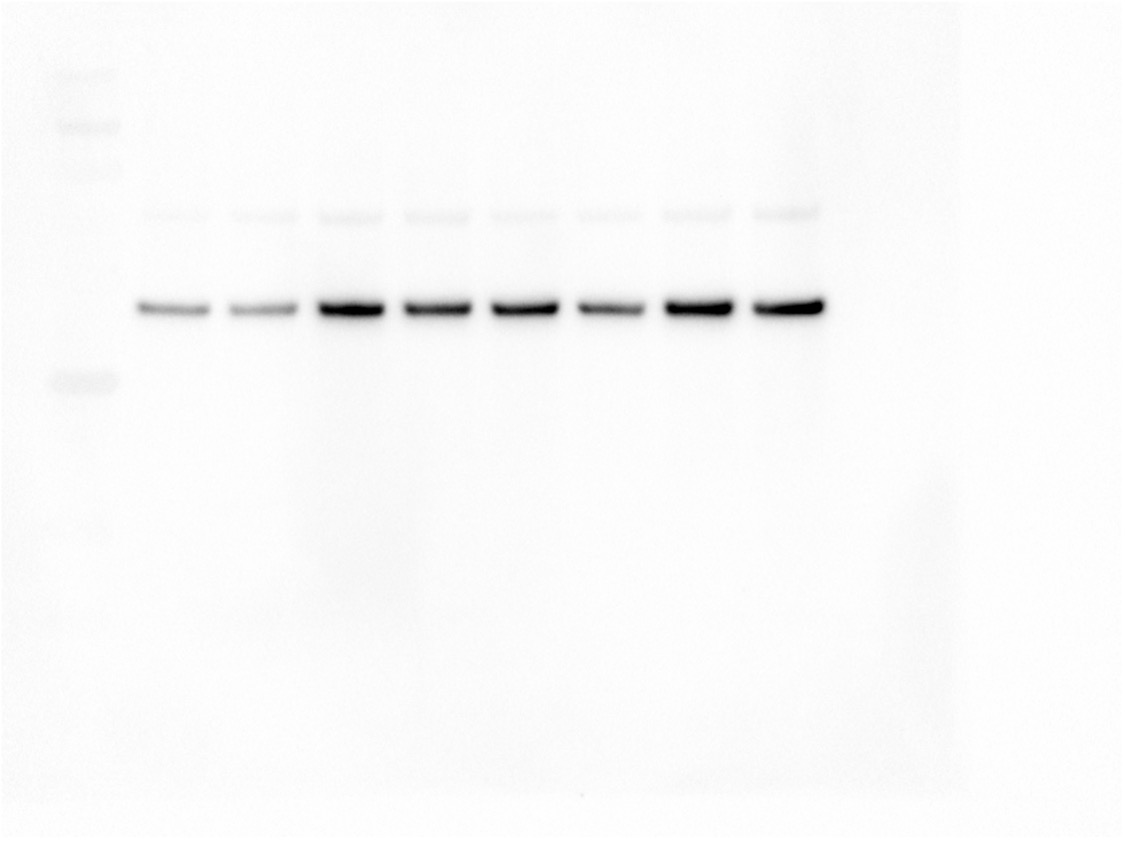

Supplement: Figure 4—source data 3. [file elife-96519-fig4-data3.zip › Actin 4G .jpg]

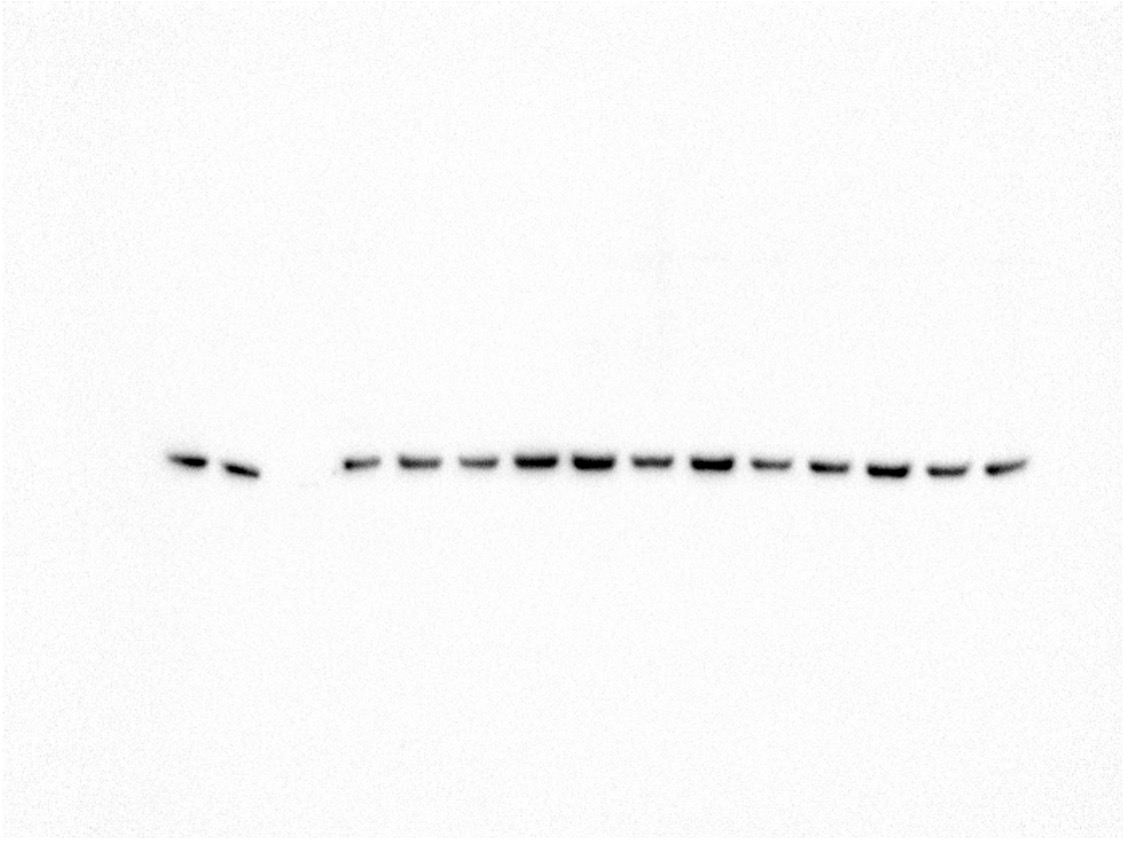

Supplement: Figure 4—source data 3. [file elife-96519-fig4-data3.zip › Actin RIT1 line 4A .jpg]

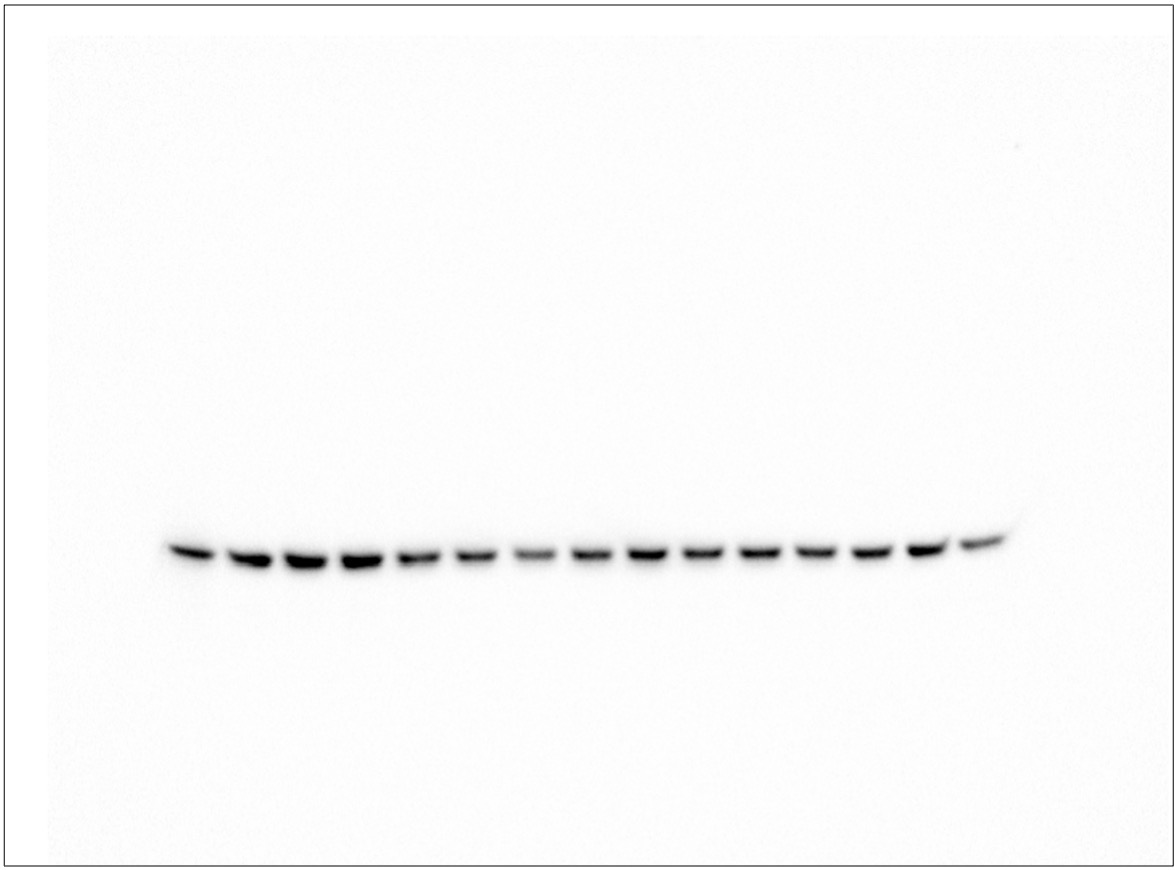

Supplement: Figure 4—source data 3. [file elife-96519-fig4-data3.zip › Actine-CBL line 4A .jpg]

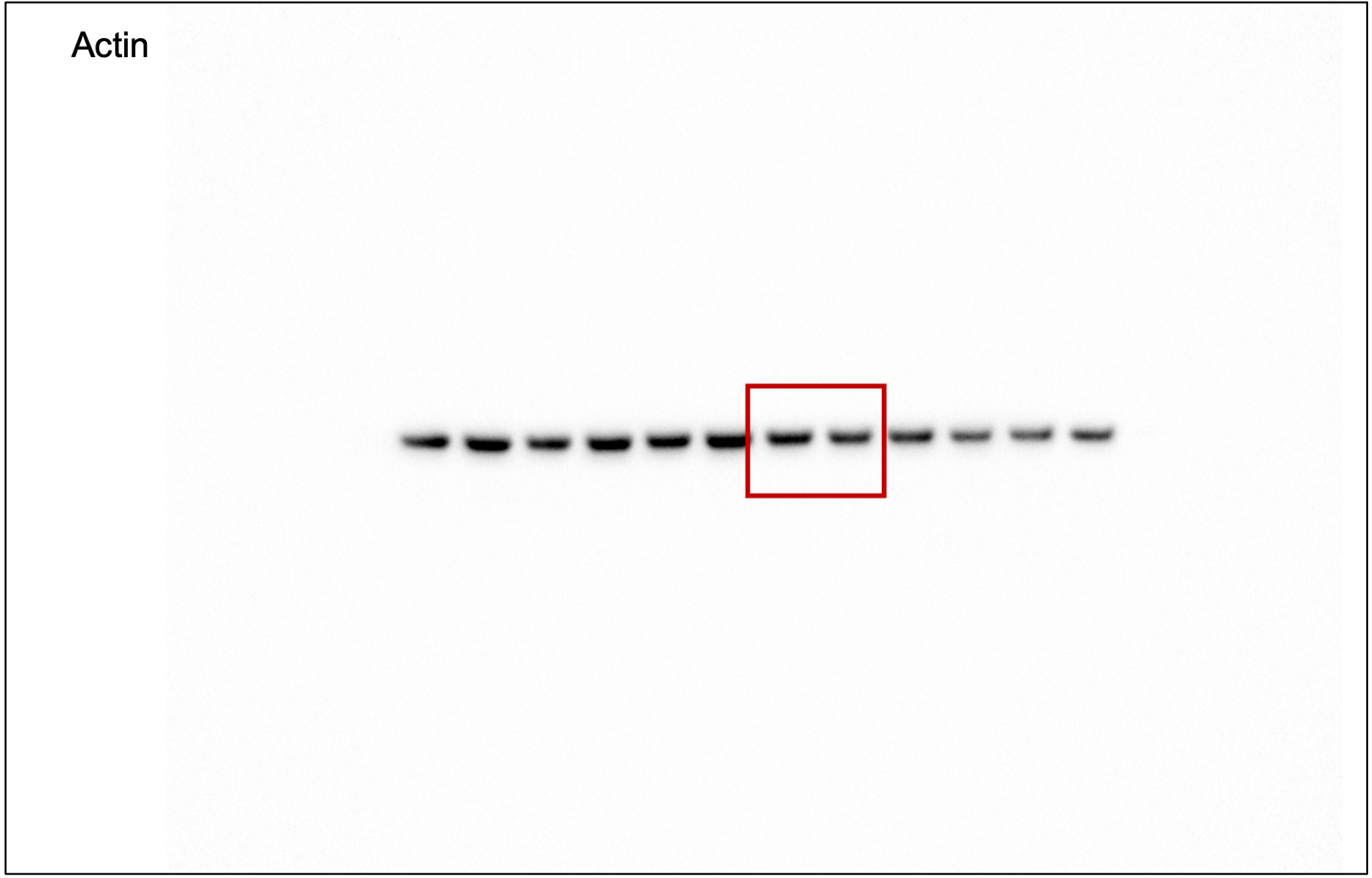

Supplement: Figure 4—source data 3. [file elife-96519-fig4-data3.zip › Actine-KRAS line 4A .jpg]

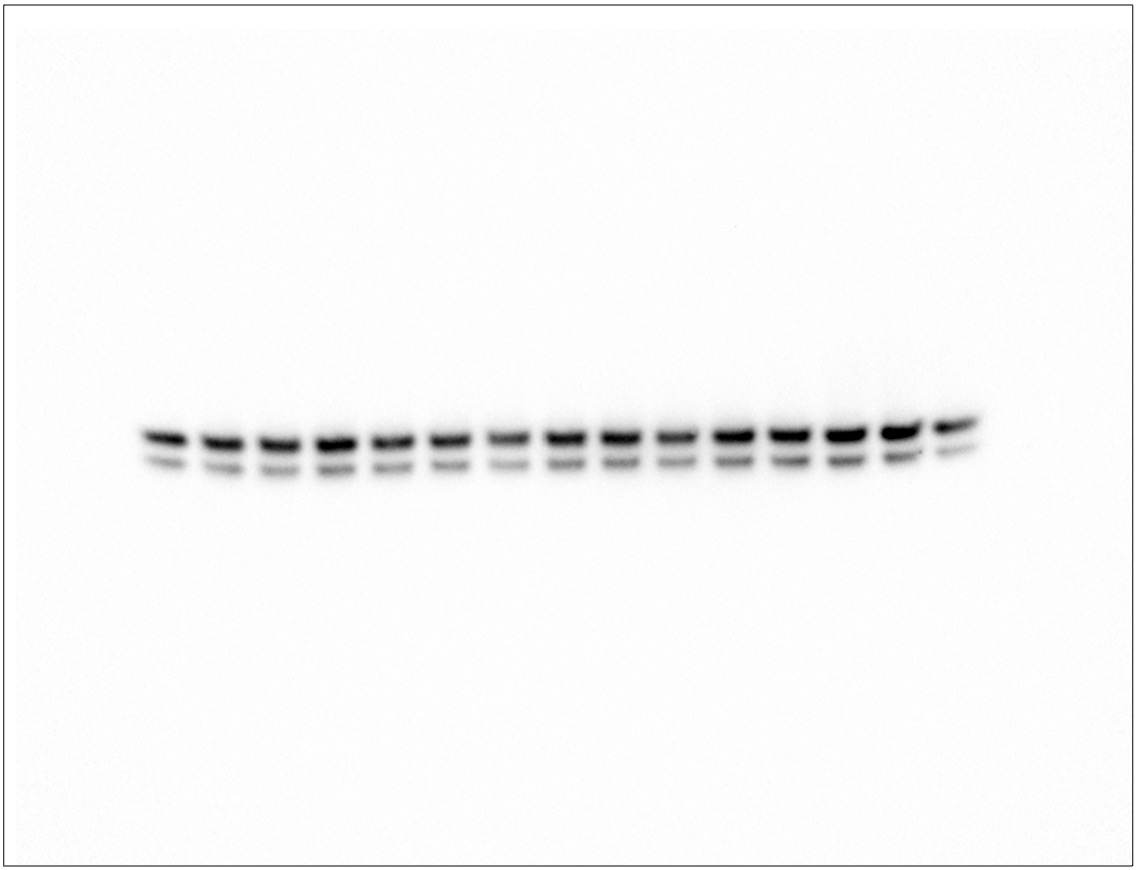

Supplement: Figure 4—source data 3. [file elife-96519-fig4-data3.zip › ERK-CBL line 4A .jpg]

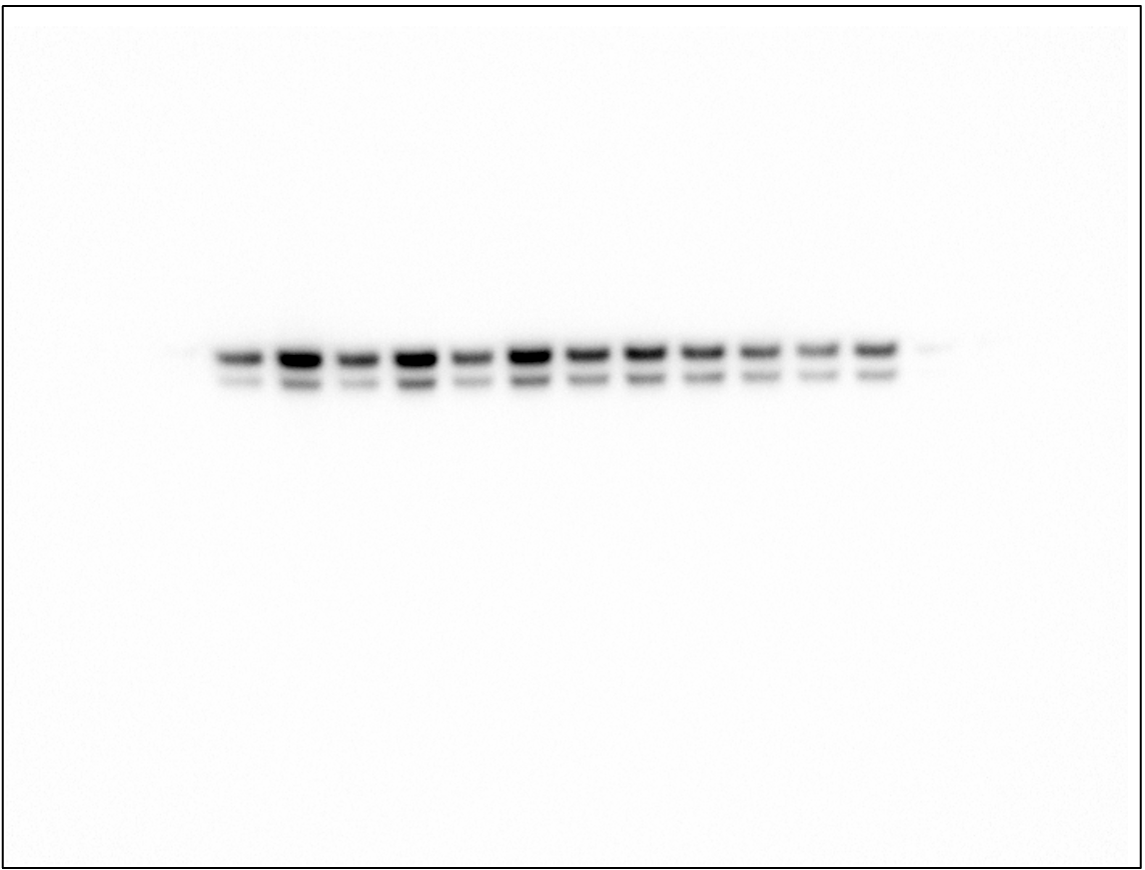

Supplement: Figure 4—source data 3. [file elife-96519-fig4-data3.zip › ERK-KRAS line 4A .jpg]

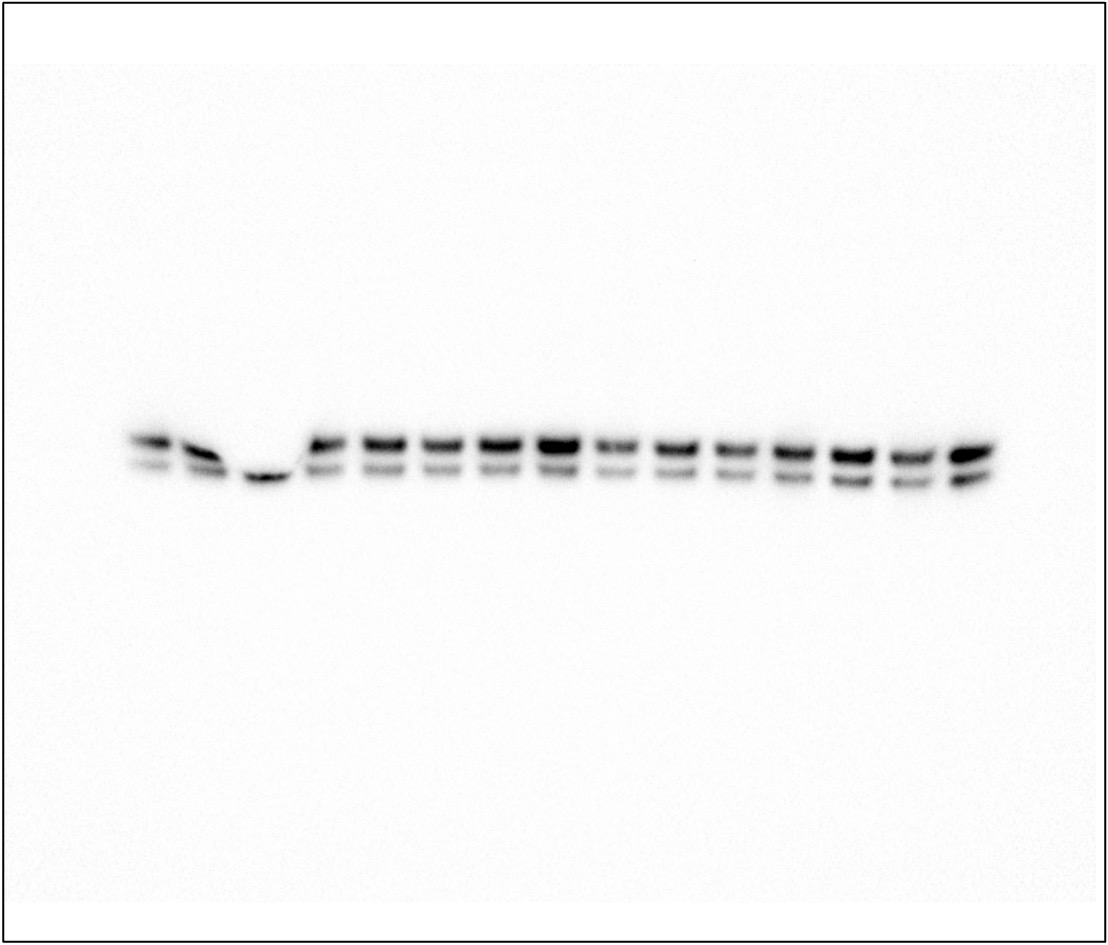

Supplement: Figure 4—source data 3. [file elife-96519-fig4-data3.zip › ERK-RIT1 line 4A .jpg]

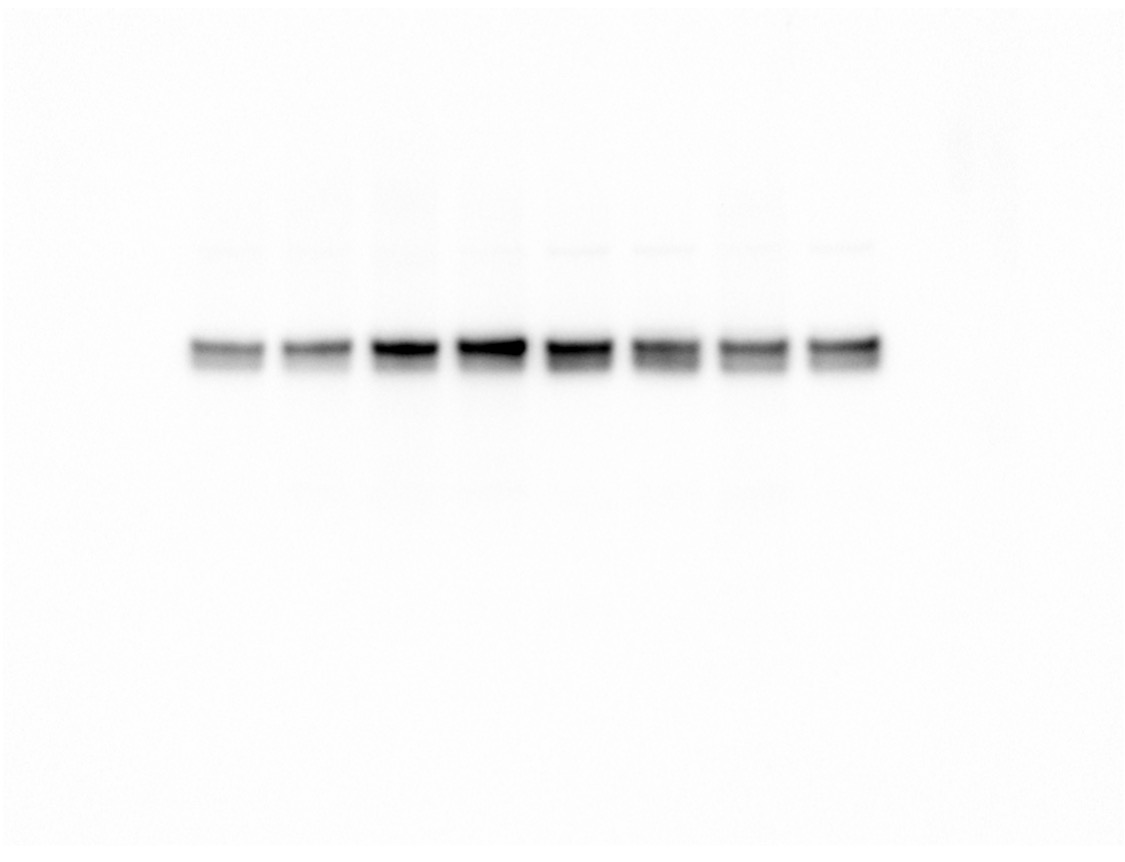

Supplement: Figure 4—source data 3. [file elife-96519-fig4-data3.zip › ERK1:2 4G.jpg]

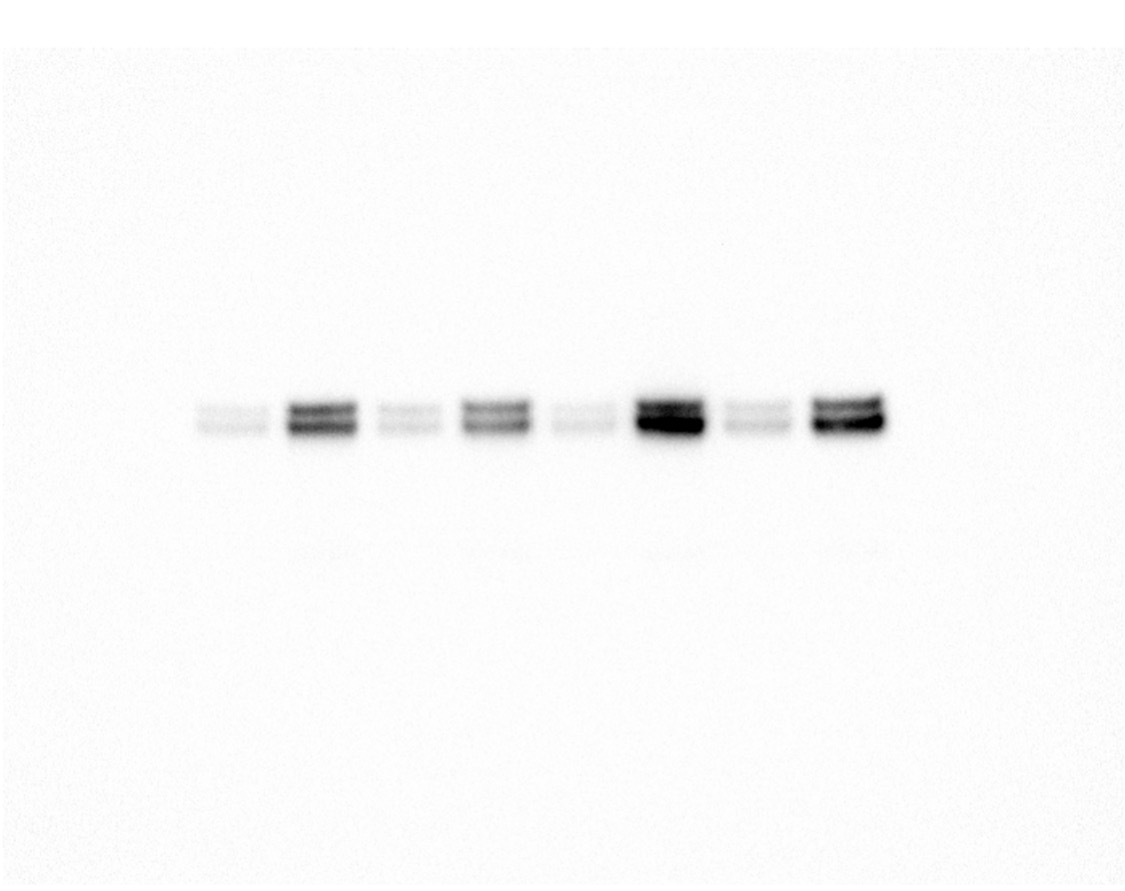

Supplement: Figure 4—source data 3. [file elife-96519-fig4-data3.zip › pERK 4G .jpg]

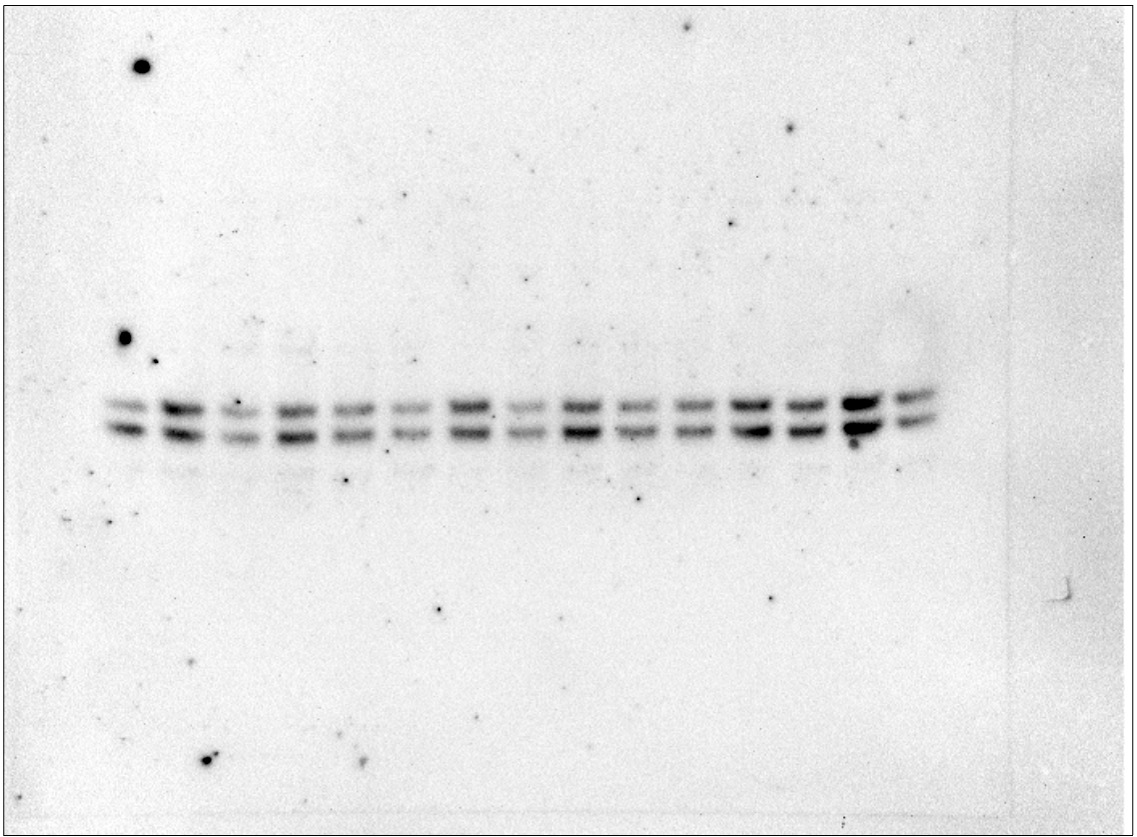

Supplement: Figure 4—source data 3. [file elife-96519-fig4-data3.zip › pERK-CBL line 4A .jpg]

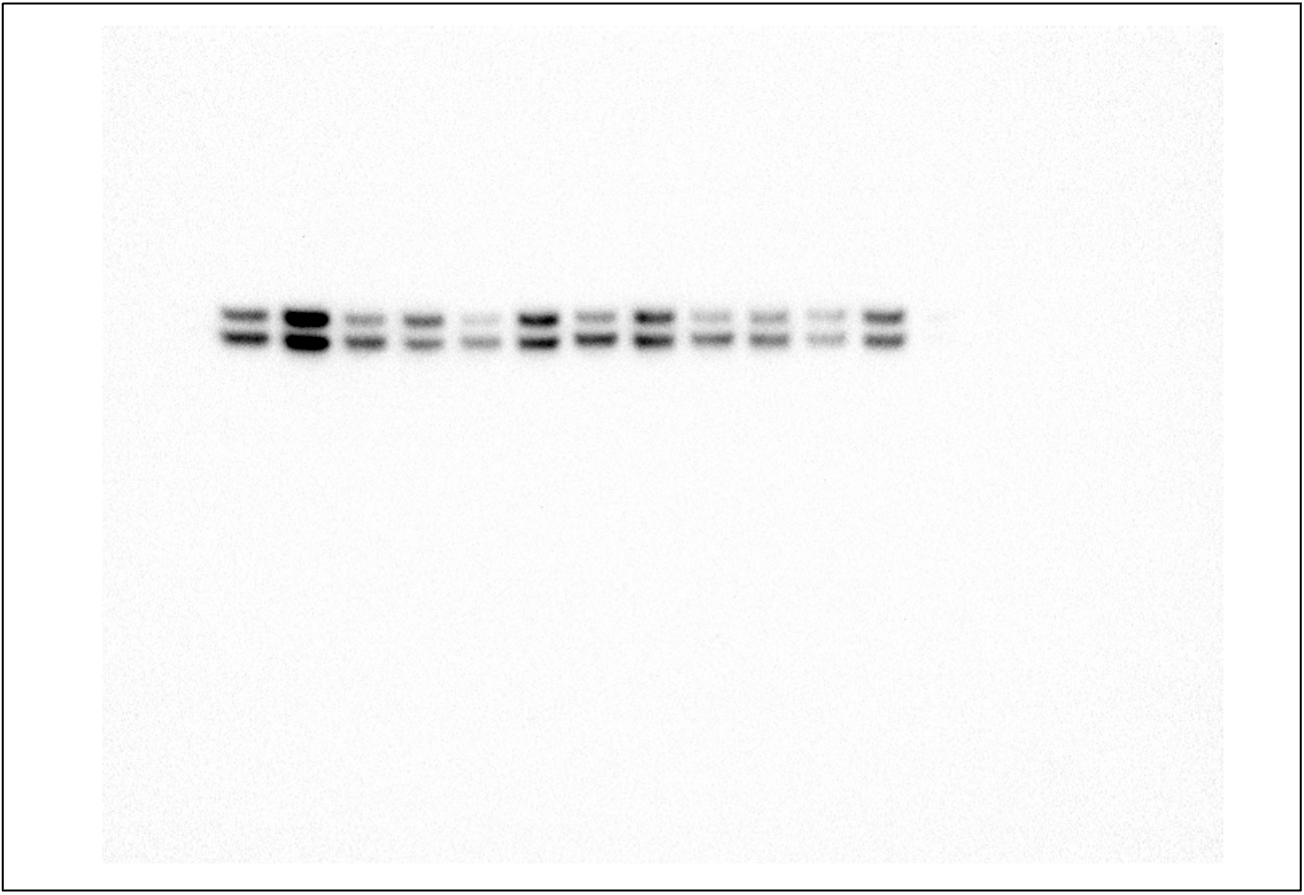

Supplement: Figure 4—source data 3. [file elife-96519-fig4-data3.zip › pERK-KRAS line 4A .jpg]

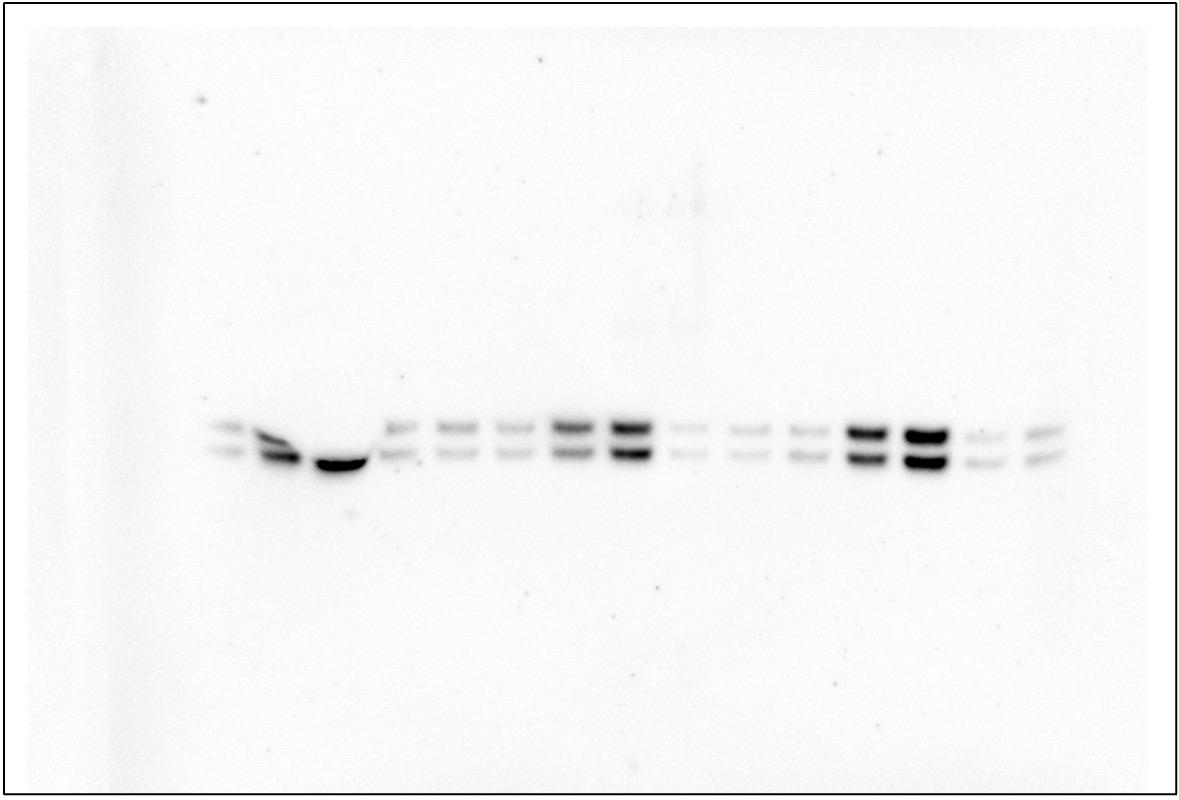

Supplement: Figure 4—source data 3. [file elife-96519-fig4-data3.zip › pERK-RIT1 line 4A .jpg]

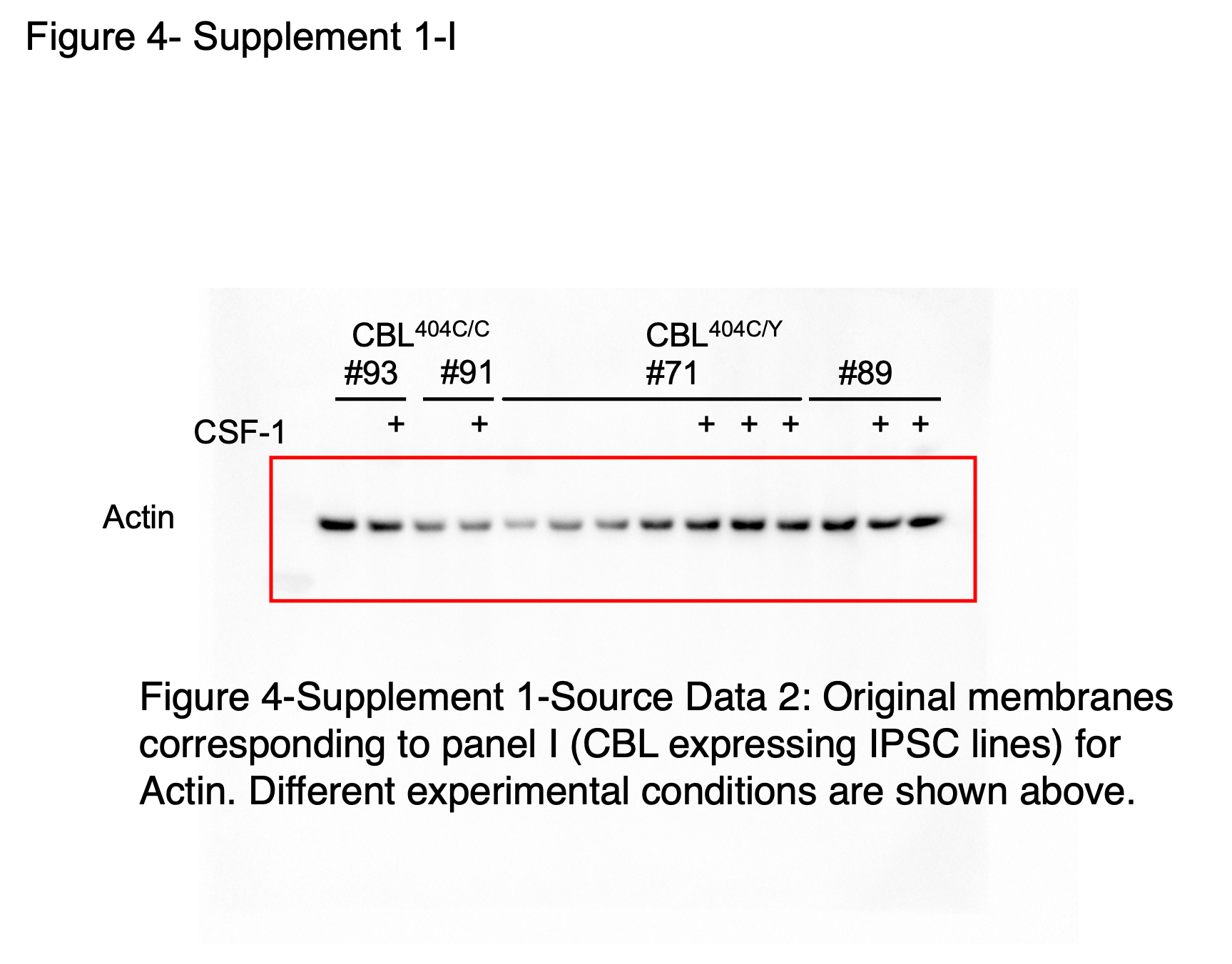

Supplement: Figure 4—figure supplement 1—source data 2. [file elife-96519-fig4-figsupp1-data2.zip › Actin-Figure 4- Supplement 1-I-cbl ipsc line .png]

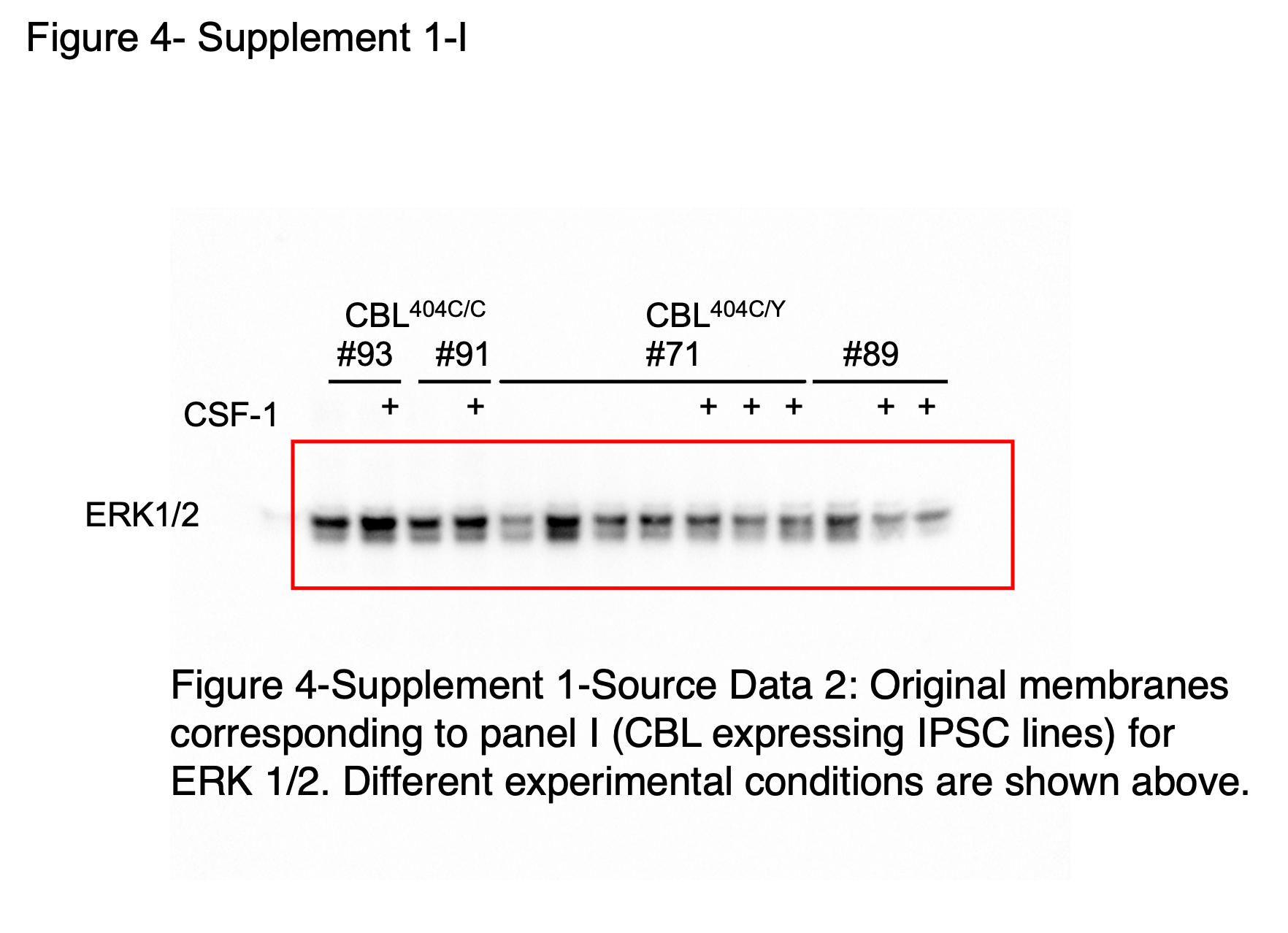

Supplement: Figure 4—figure supplement 1—source data 2. [file elife-96519-fig4-figsupp1-data2.zip › total ERK-Figure 4- Supplement 1-I-cbl ipsc line .png]

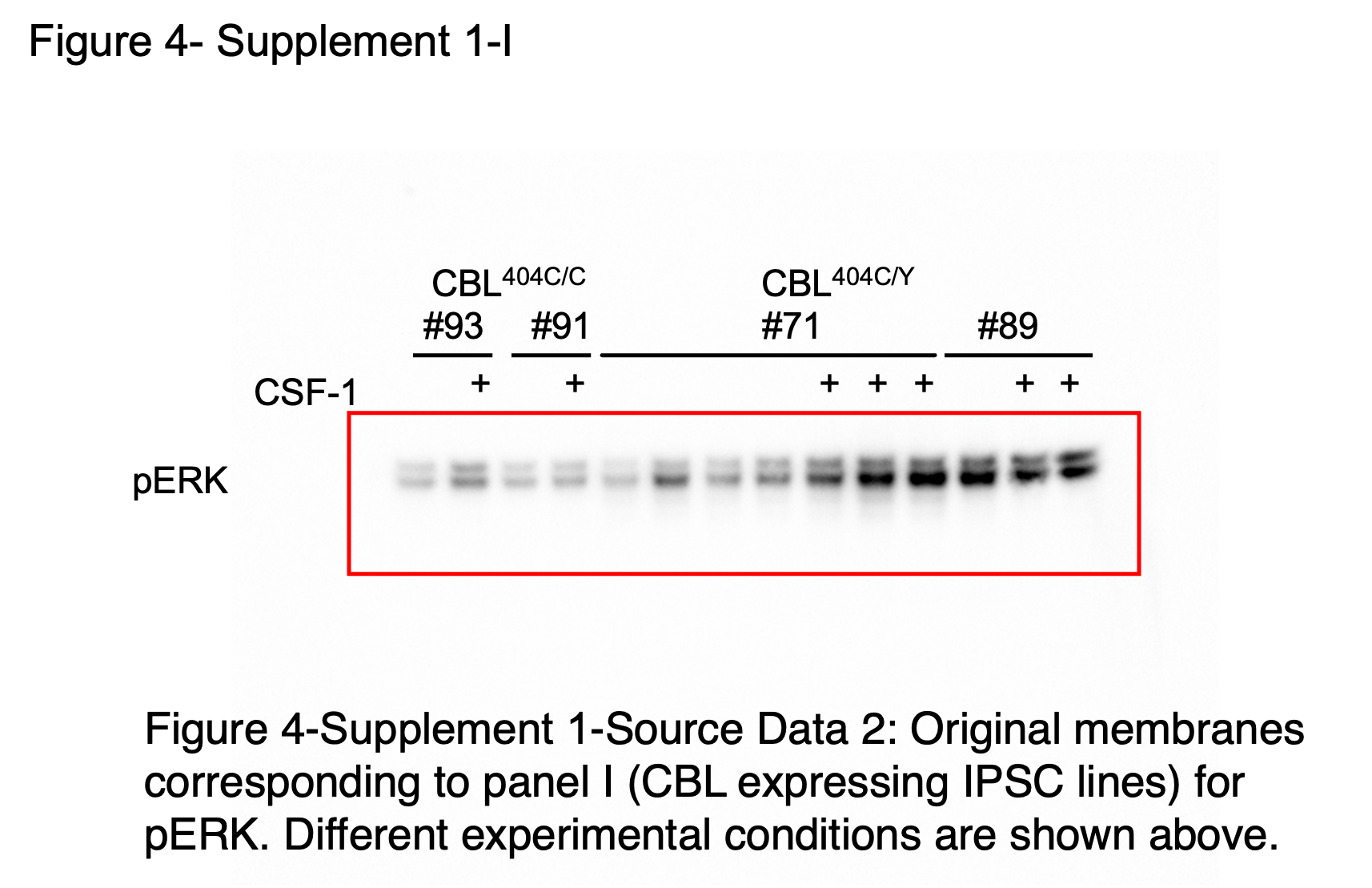

Supplement: Figure 4—figure supplement 1—source data 2. [file elife-96519-fig4-figsupp1-data2.zip › pERK-Figure 4- Supplement 1-I-cbl ipsc line .png]

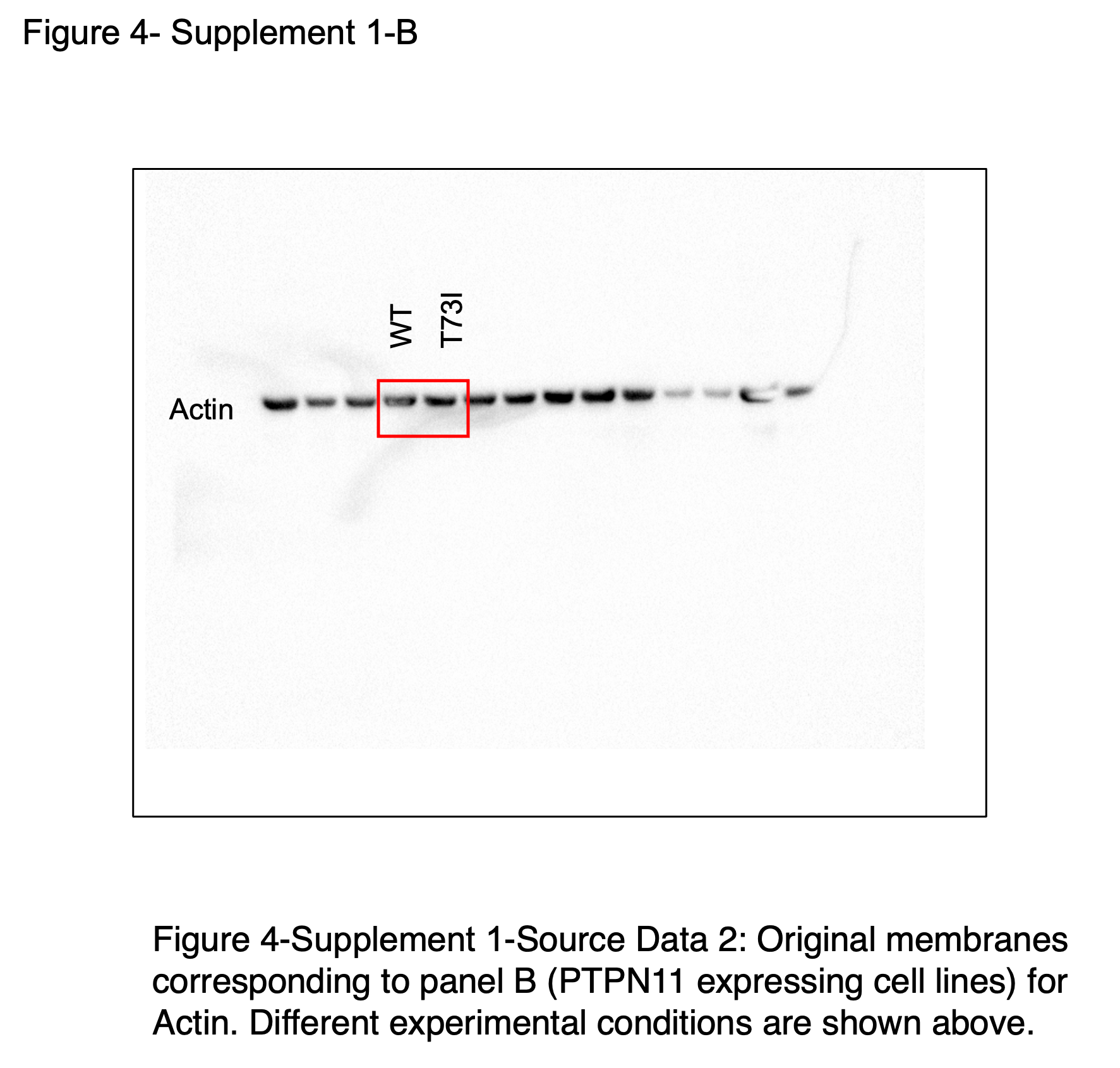

Supplement: Figure 4—figure supplement 1—source data 2. [file elife-96519-fig4-figsupp1-data2.zip › Actin-Figure 4- Supplement 1-B-PTPN11 line 2 .png]

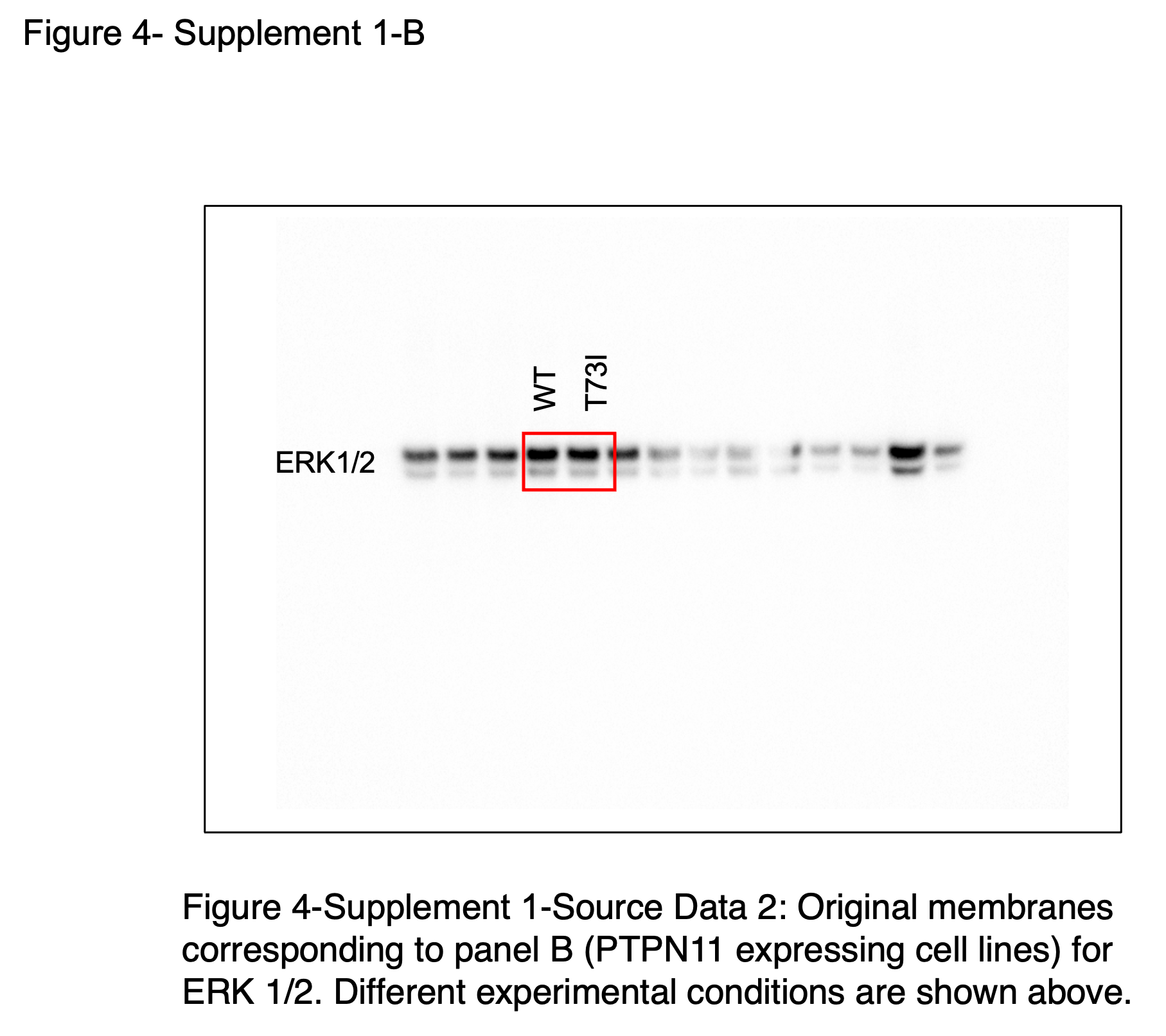

Supplement: Figure 4—figure supplement 1—source data 2. [file elife-96519-fig4-figsupp1-data2.zip › ERK1:2-Figure 4- Supplement 1-B-PTPN11 line .png]

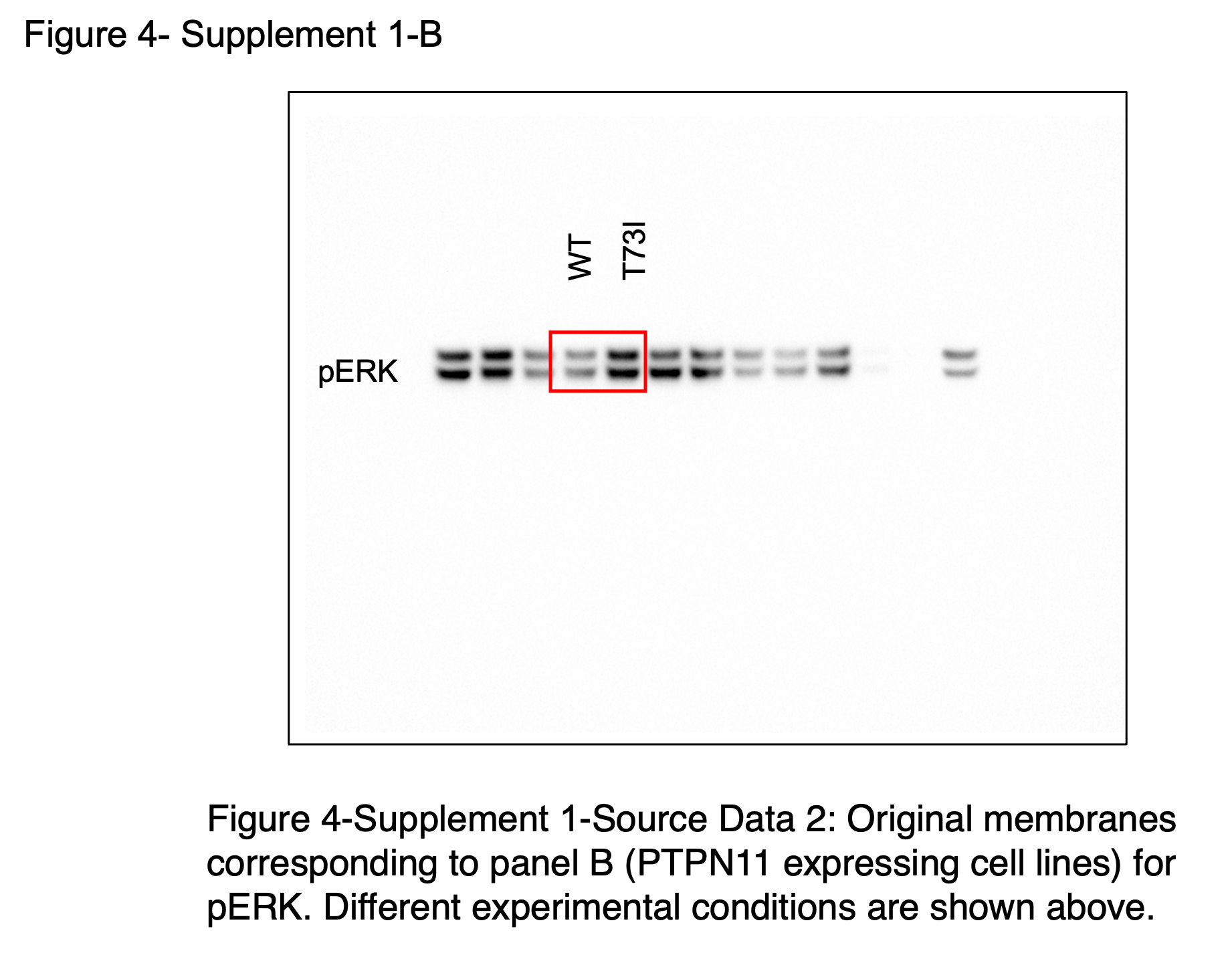

Supplement: Figure 4—figure supplement 1—source data 2. [file elife-96519-fig4-figsupp1-data2.zip › pERK-Figure 4- Supplement 1-B-PTPN11 line .png]

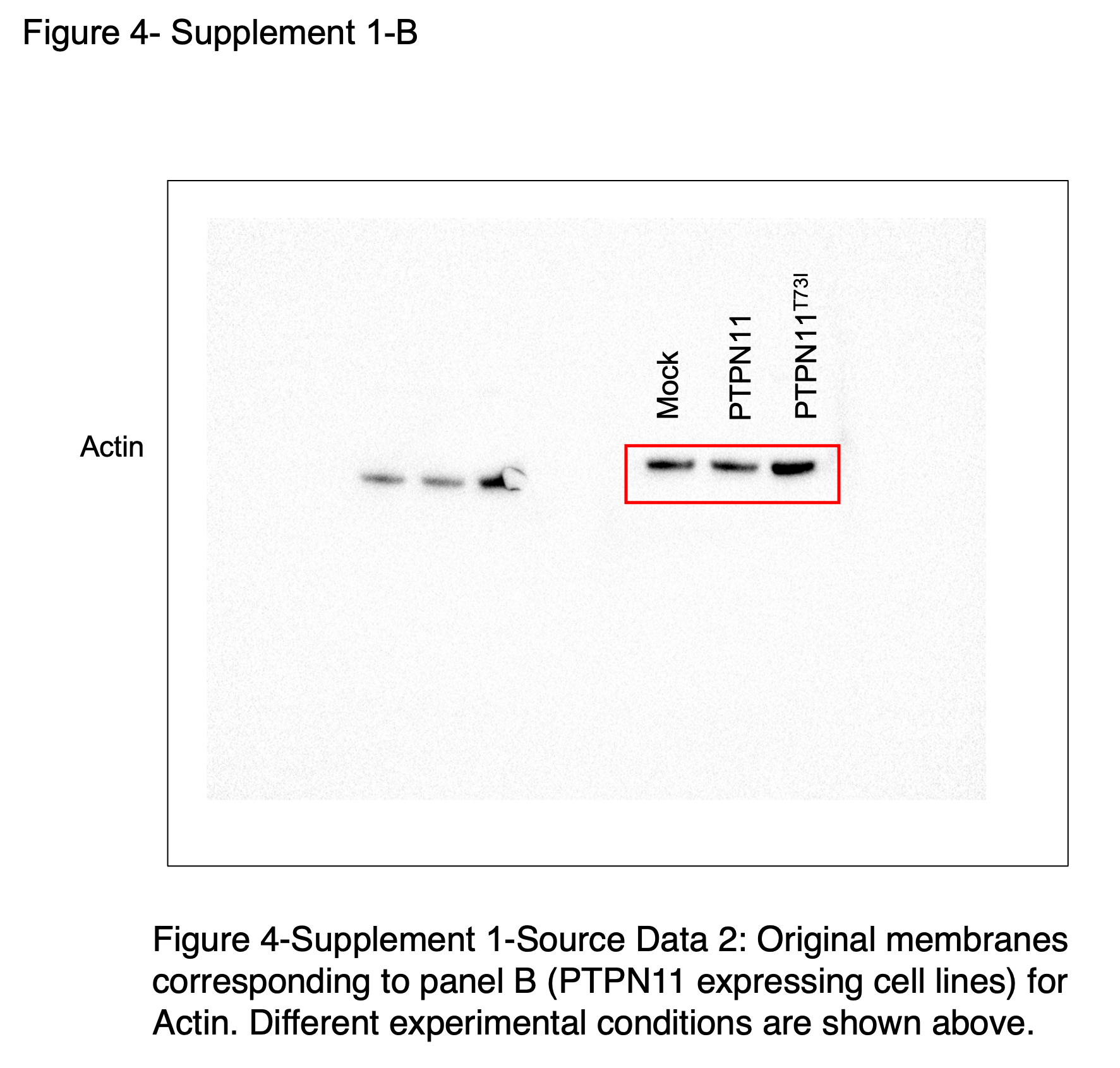

Supplement: Figure 4—figure supplement 1—source data 2. [file elife-96519-fig4-figsupp1-data2.zip › Actin-Figure 4- Supplement 1-B-PTPN11 line .png]

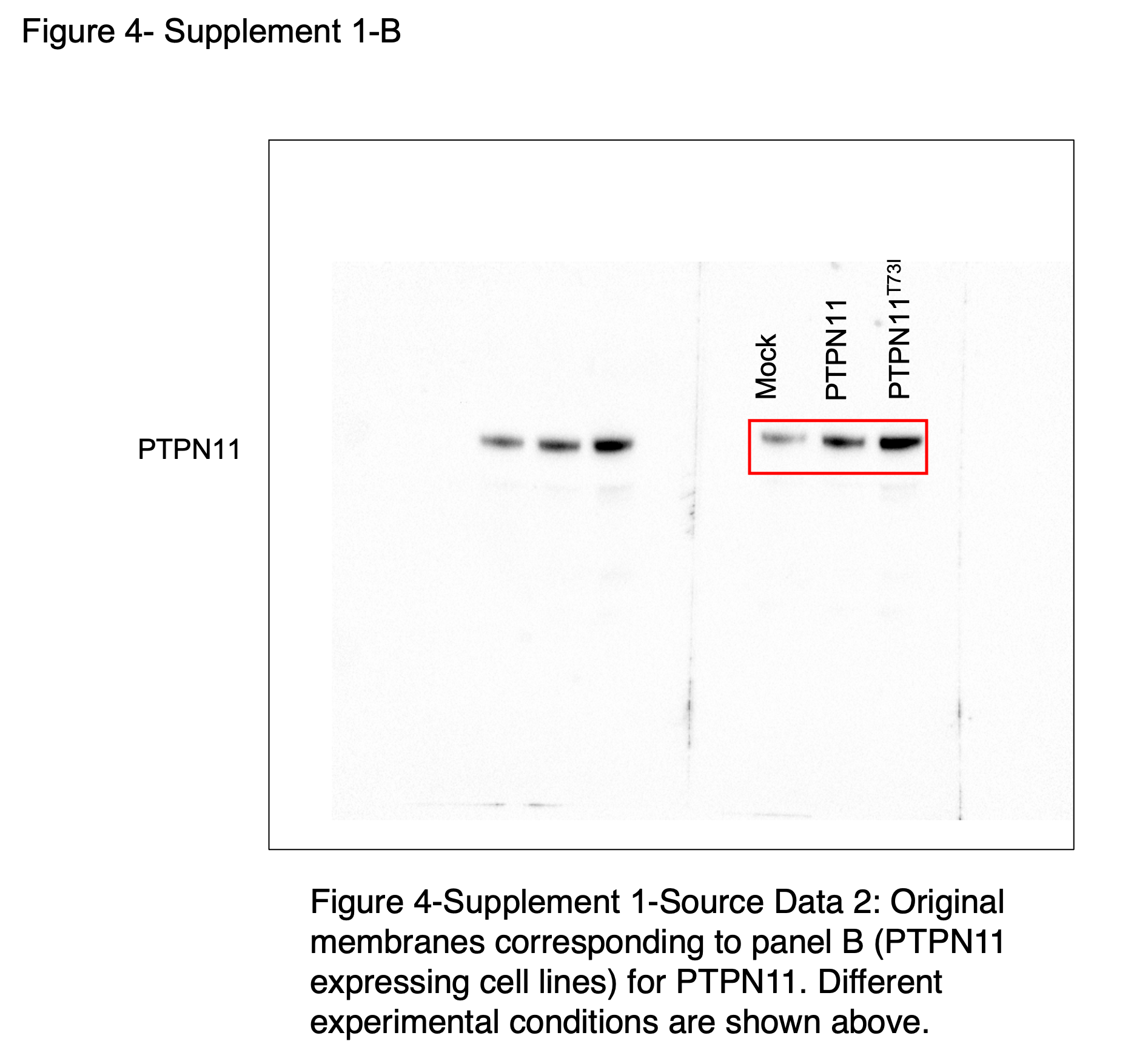

Supplement: Figure 4—figure supplement 1—source data 2. [file elife-96519-fig4-figsupp1-data2.zip › PTPN11-Figure 4- Supplement 1-B-PTPN11 line .png]

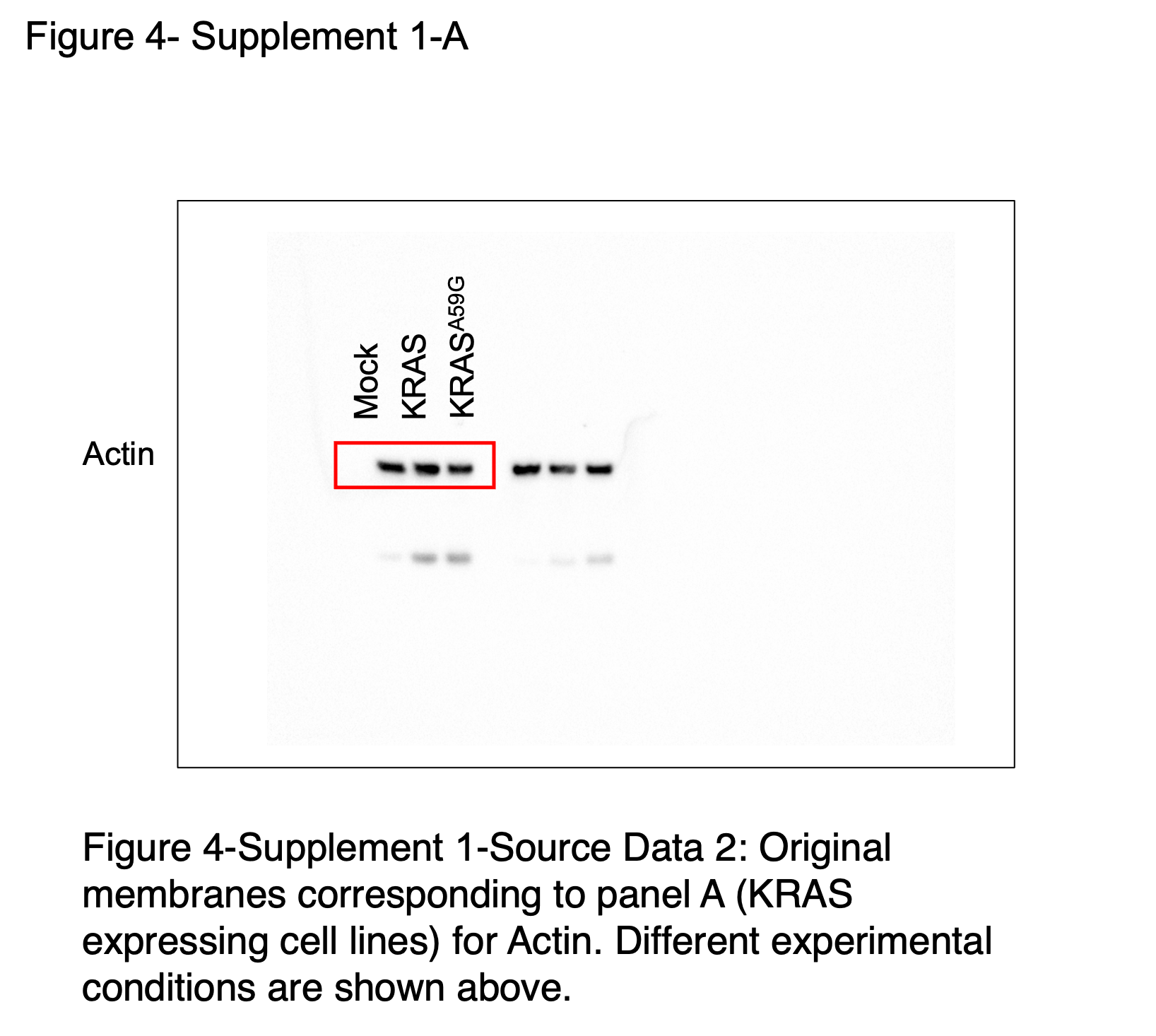

Supplement: Figure 4—figure supplement 1—source data 2. [file elife-96519-fig4-figsupp1-data2.zip › Actin-Figure 4- Supplement 1-A-KRAS line .png]

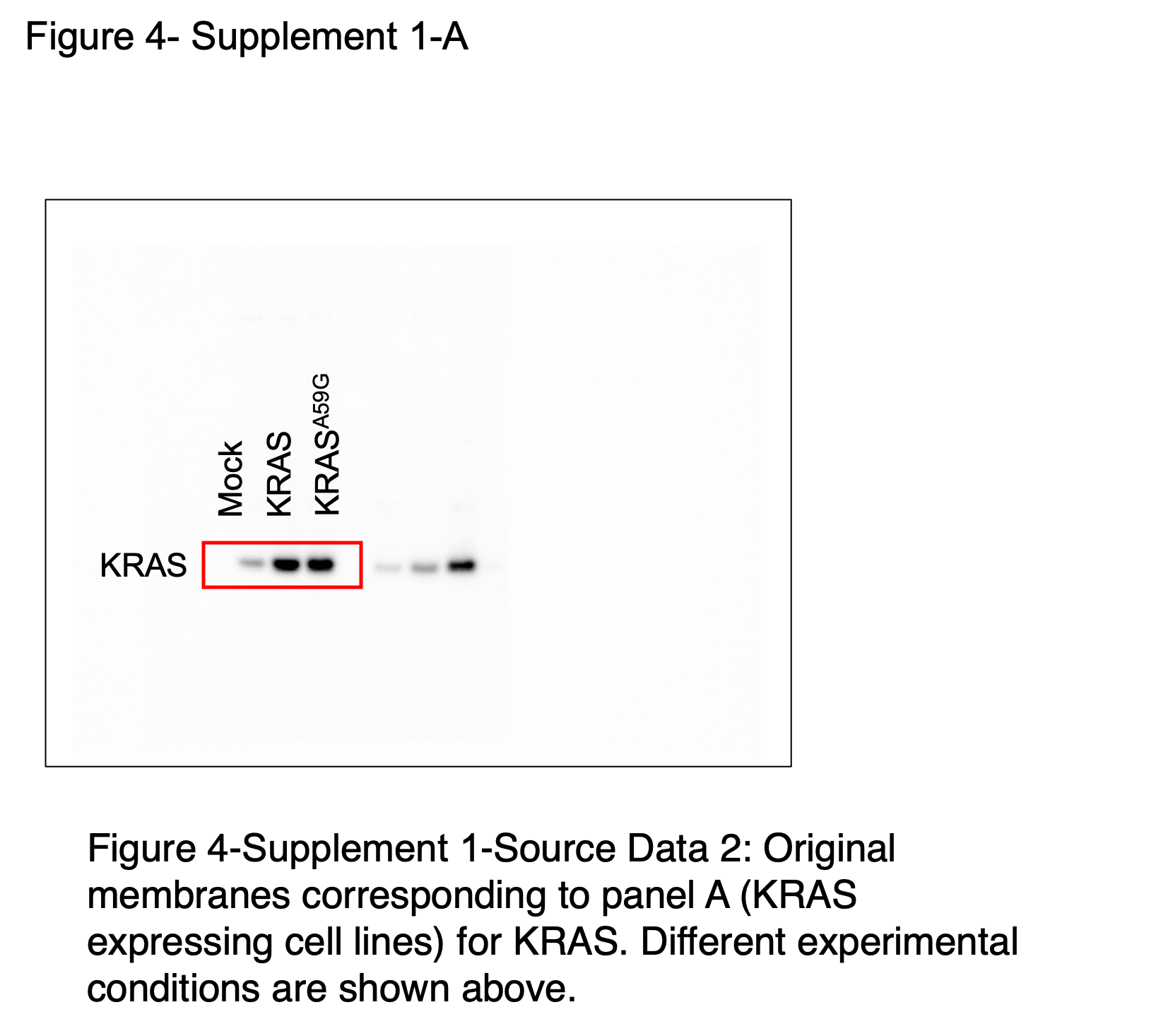

Supplement: Figure 4—figure supplement 1—source data 2. [file elife-96519-fig4-figsupp1-data2.zip › KRAS-Figure 4- Supplement 1-A-KRAS line .png]

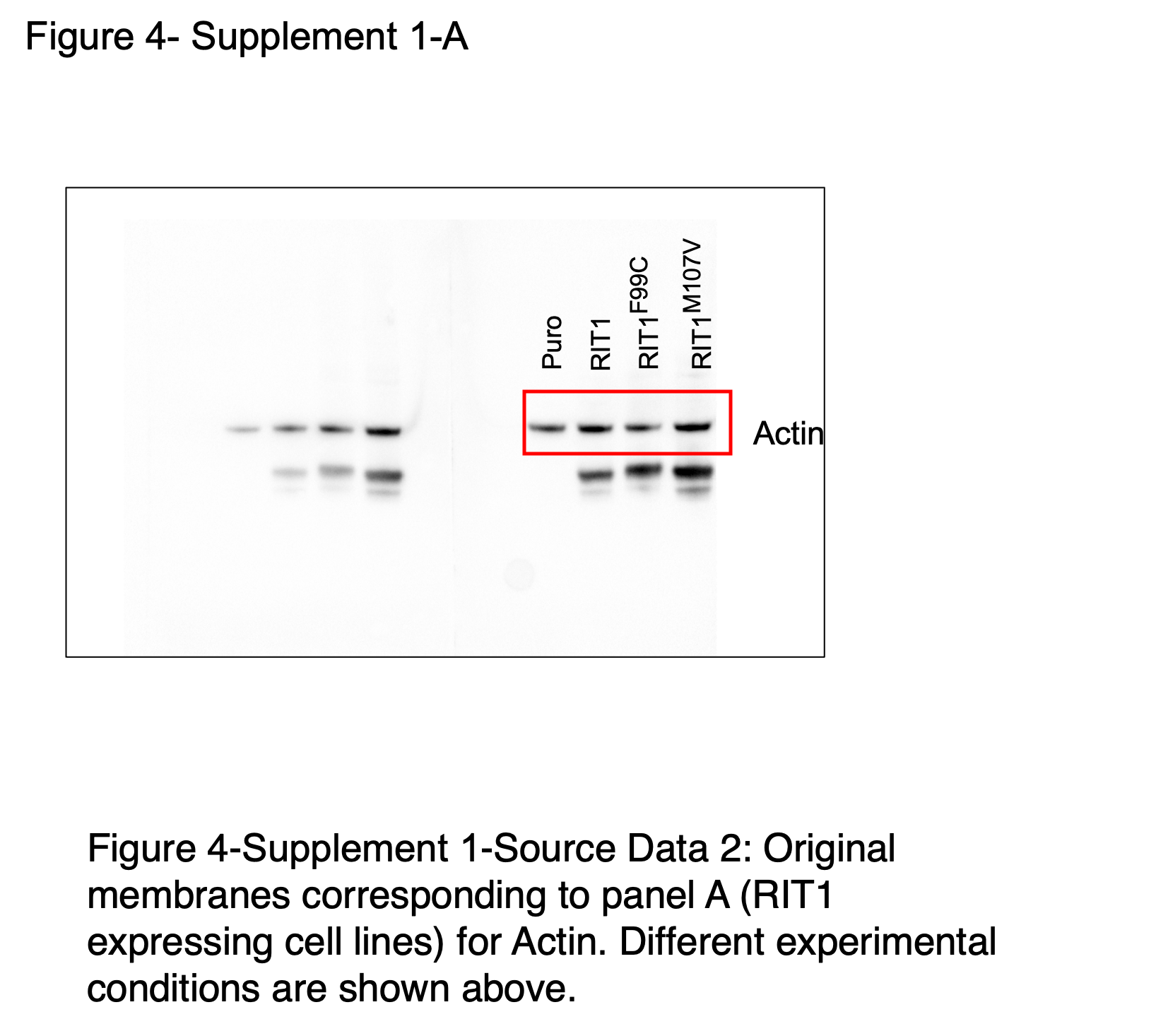

Supplement: Figure 4—figure supplement 1—source data 2. [file elife-96519-fig4-figsupp1-data2.zip › Actin-Figure 4- Supplement 1-A-RIT1 line .png]

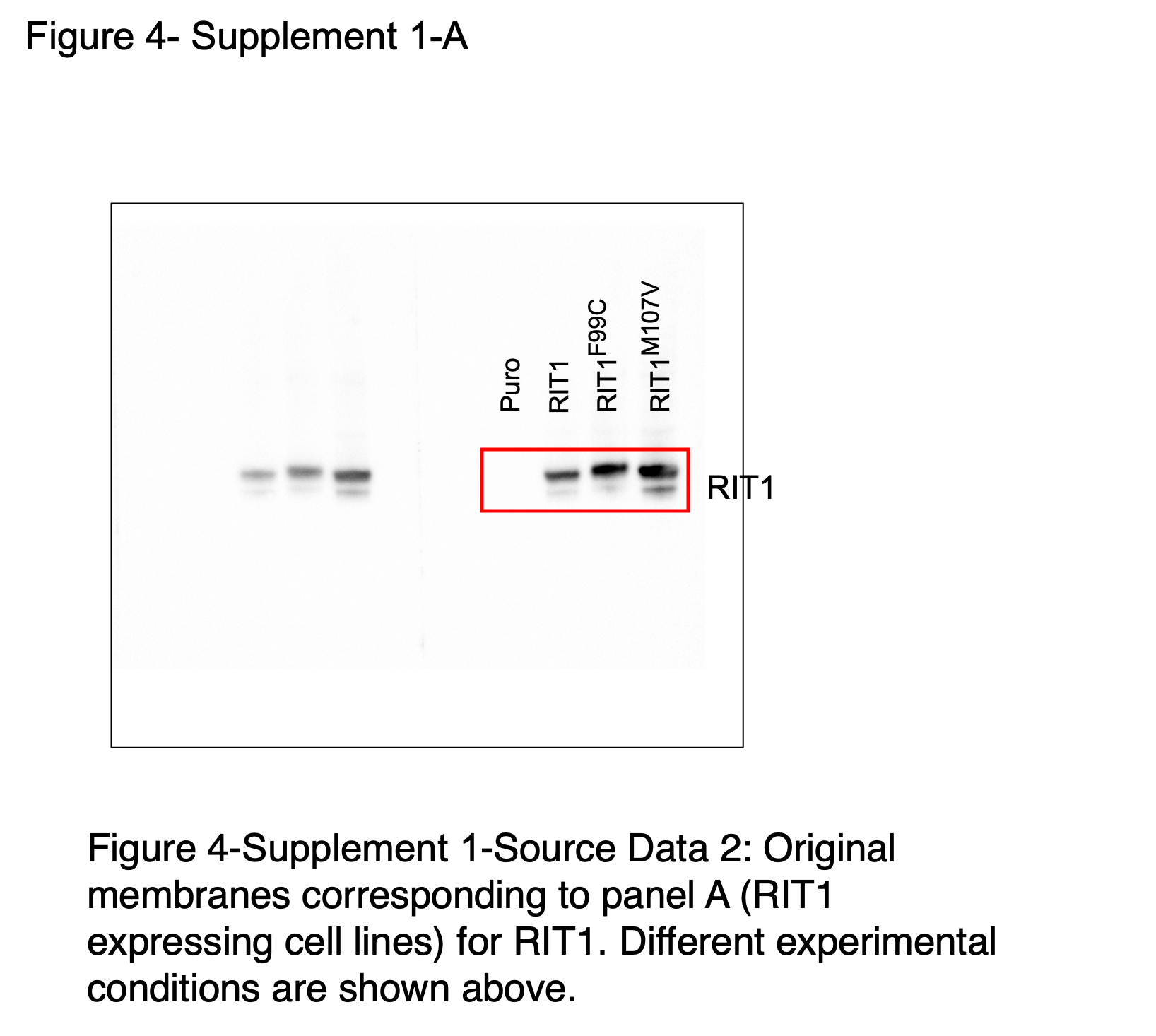

Supplement: Figure 4—figure supplement 1—source data 2. [file elife-96519-fig4-figsupp1-data2.zip › RIT1-Figure 4- Supplement 1-A-RIT1 line .png]

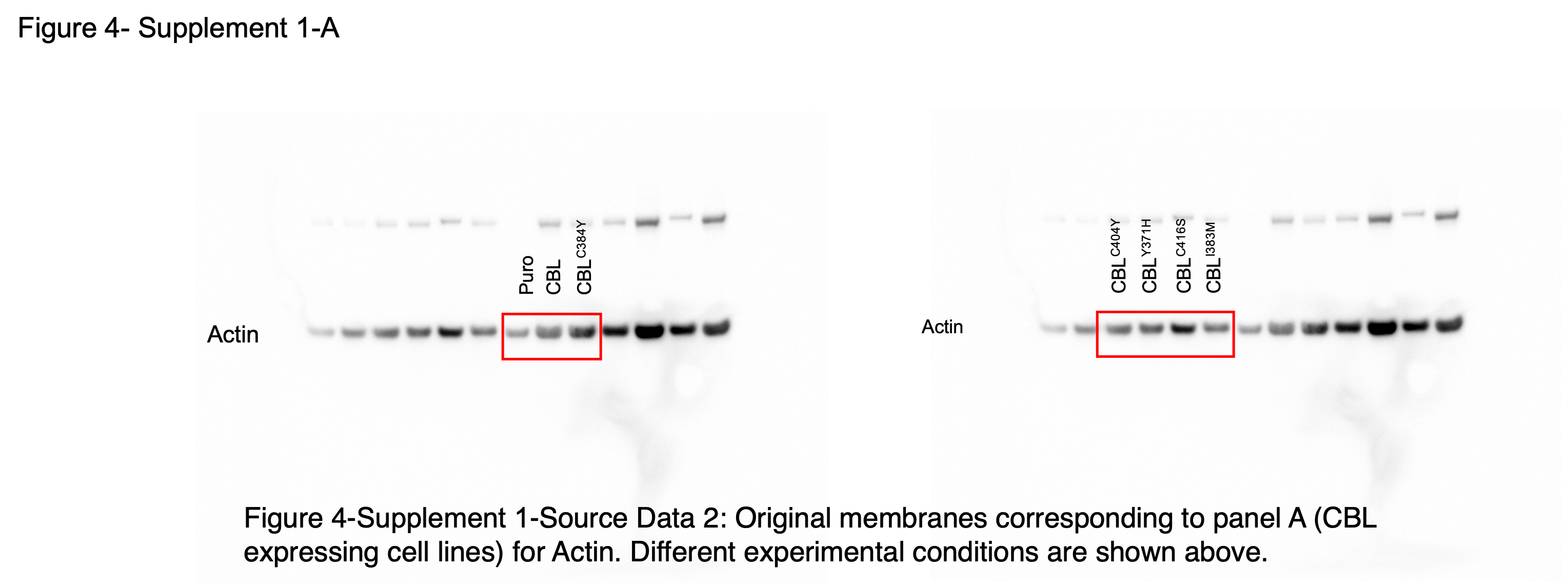

Supplement: Figure 4—figure supplement 1—source data 2. [file elife-96519-fig4-figsupp1-data2.zip › Actin-Figure 4- Supplement 1-A-CBL line .png]

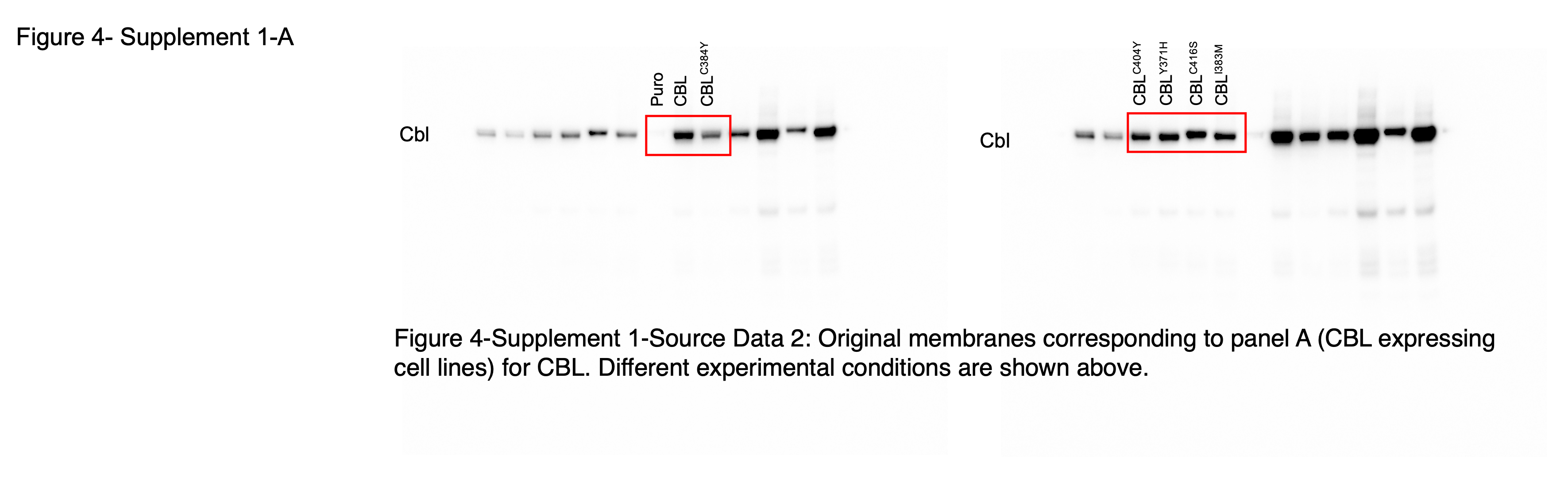

Supplement: Figure 4—figure supplement 1—source data 2. [file elife-96519-fig4-figsupp1-data2.zip › CBL-Figure 4- Supplement 1-A-CBL line .png]

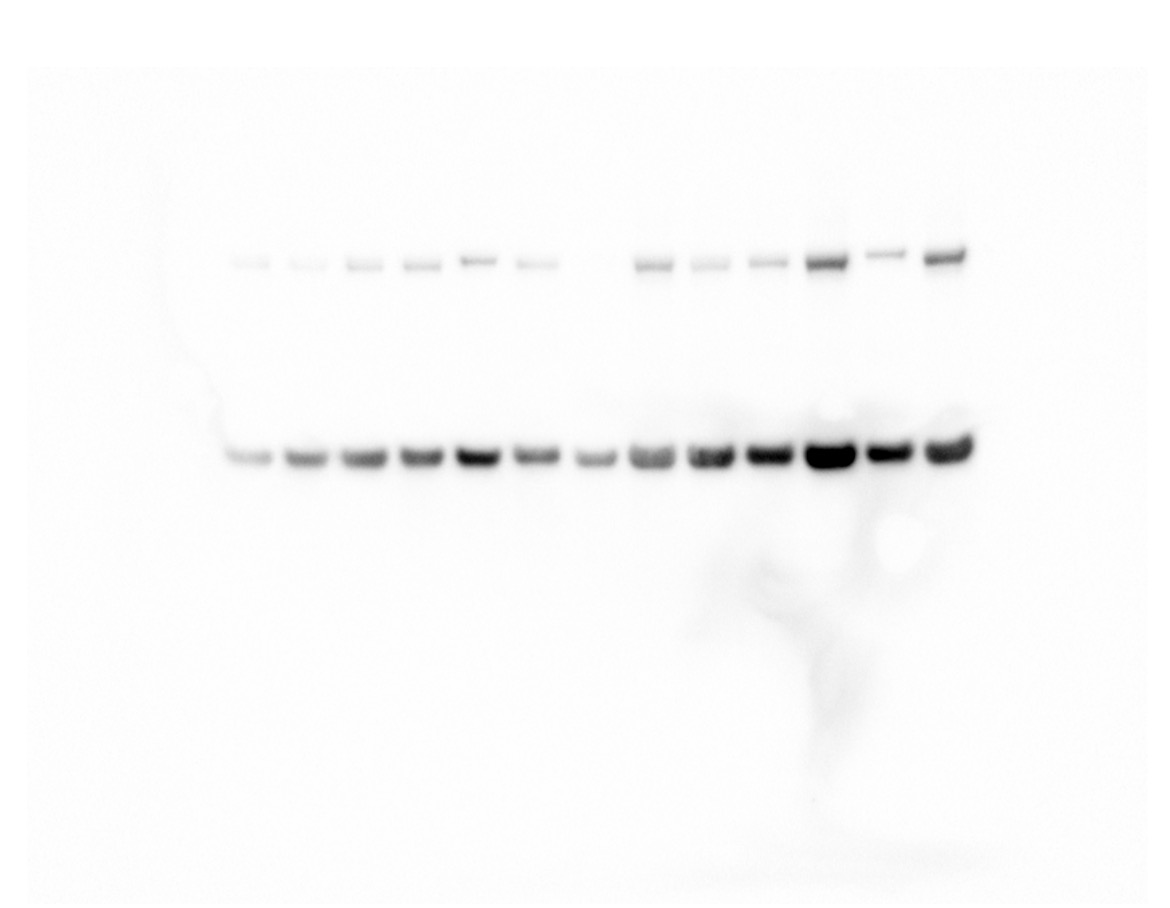

Supplement: Figure 4—figure supplement 1—source data 3. [file elife-96519-fig4-figsupp1-data3.zip › Actin cbl line A.jpg]

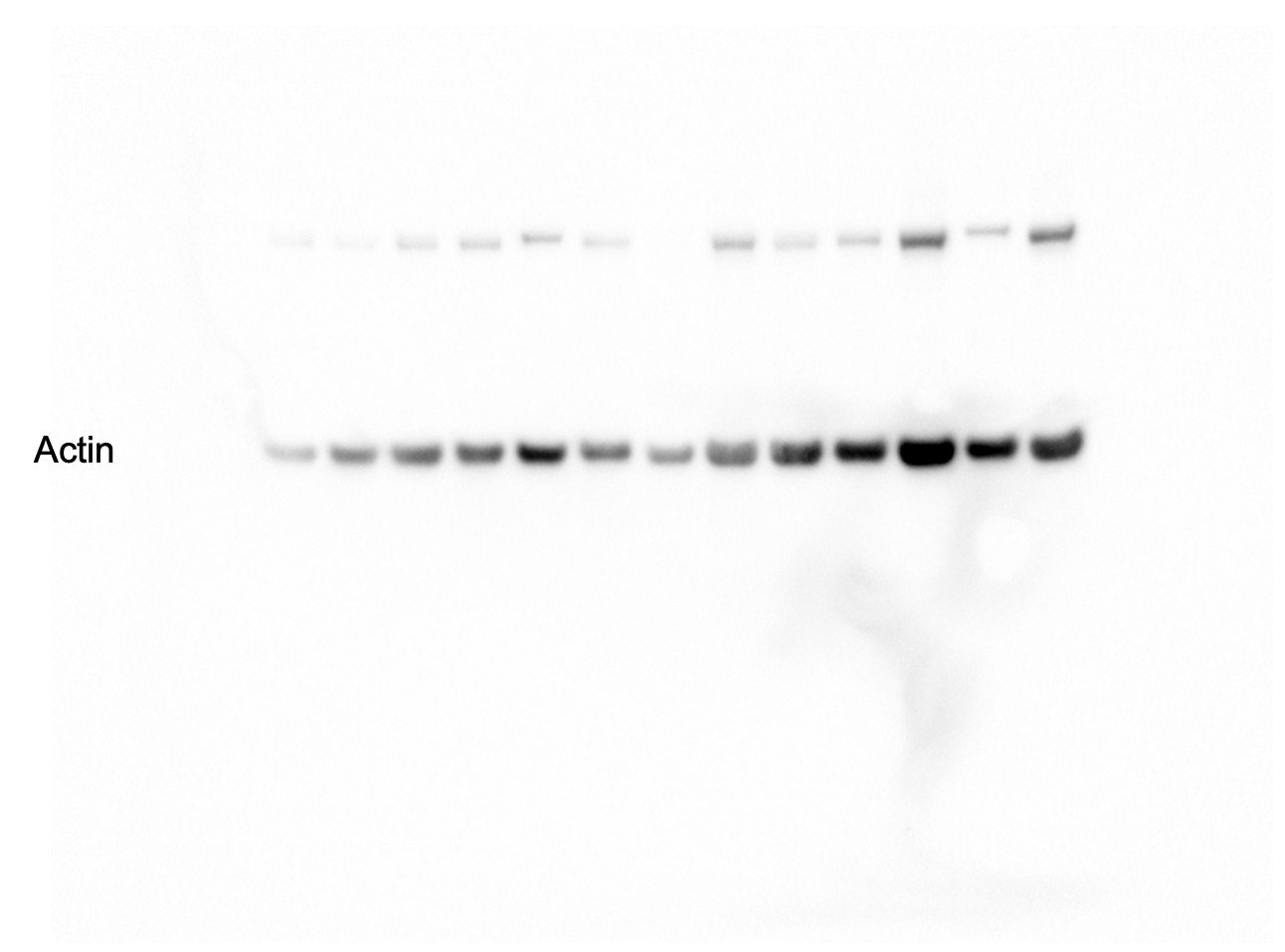

Supplement: Figure 4—figure supplement 1—source data 3. [file elife-96519-fig4-figsupp1-data3.zip › Actin cbl line2 A.jpg]

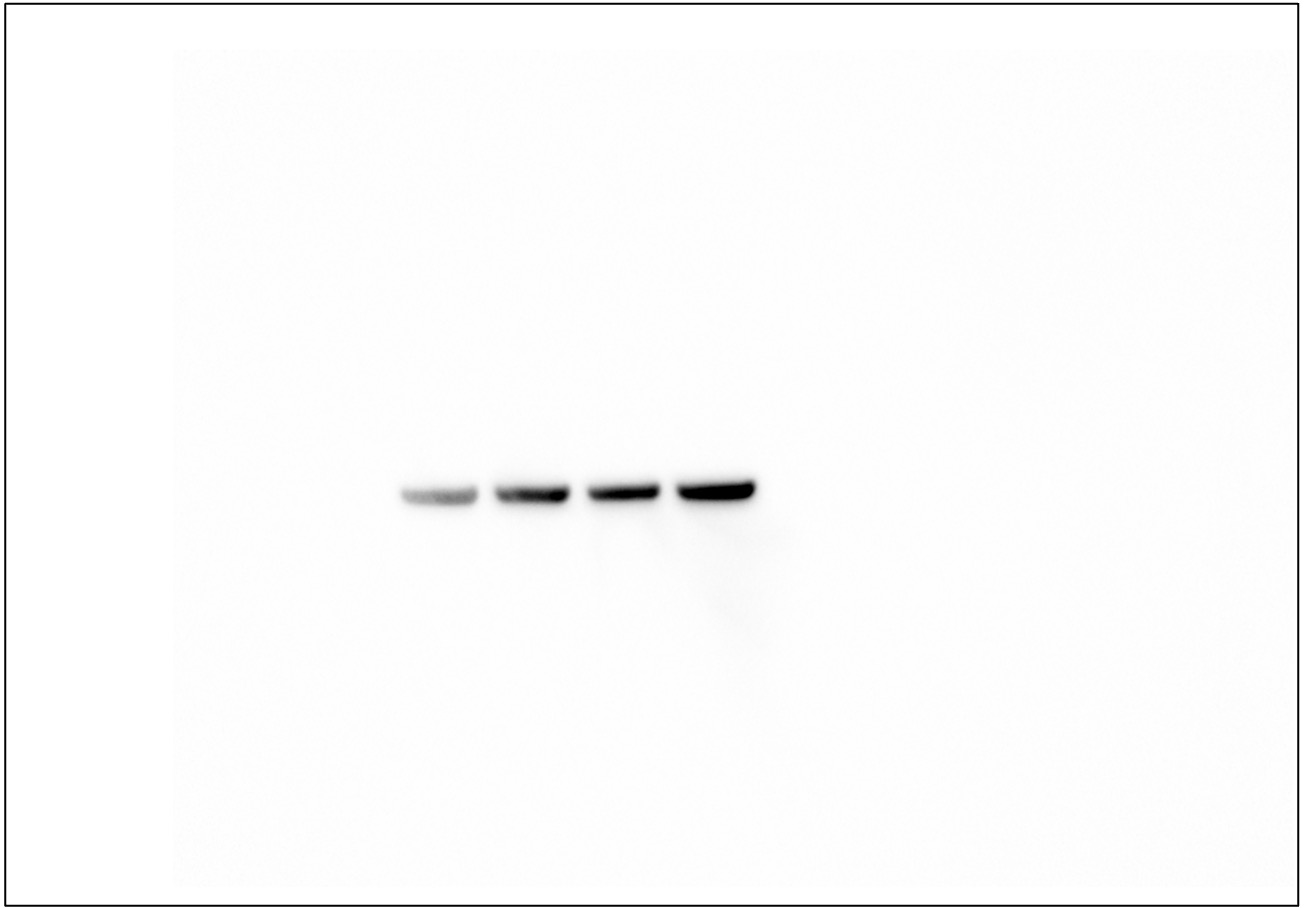

Supplement: Figure 4—figure supplement 1—source data 3. [file elife-96519-fig4-figsupp1-data3.zip › Actin F.jpg]

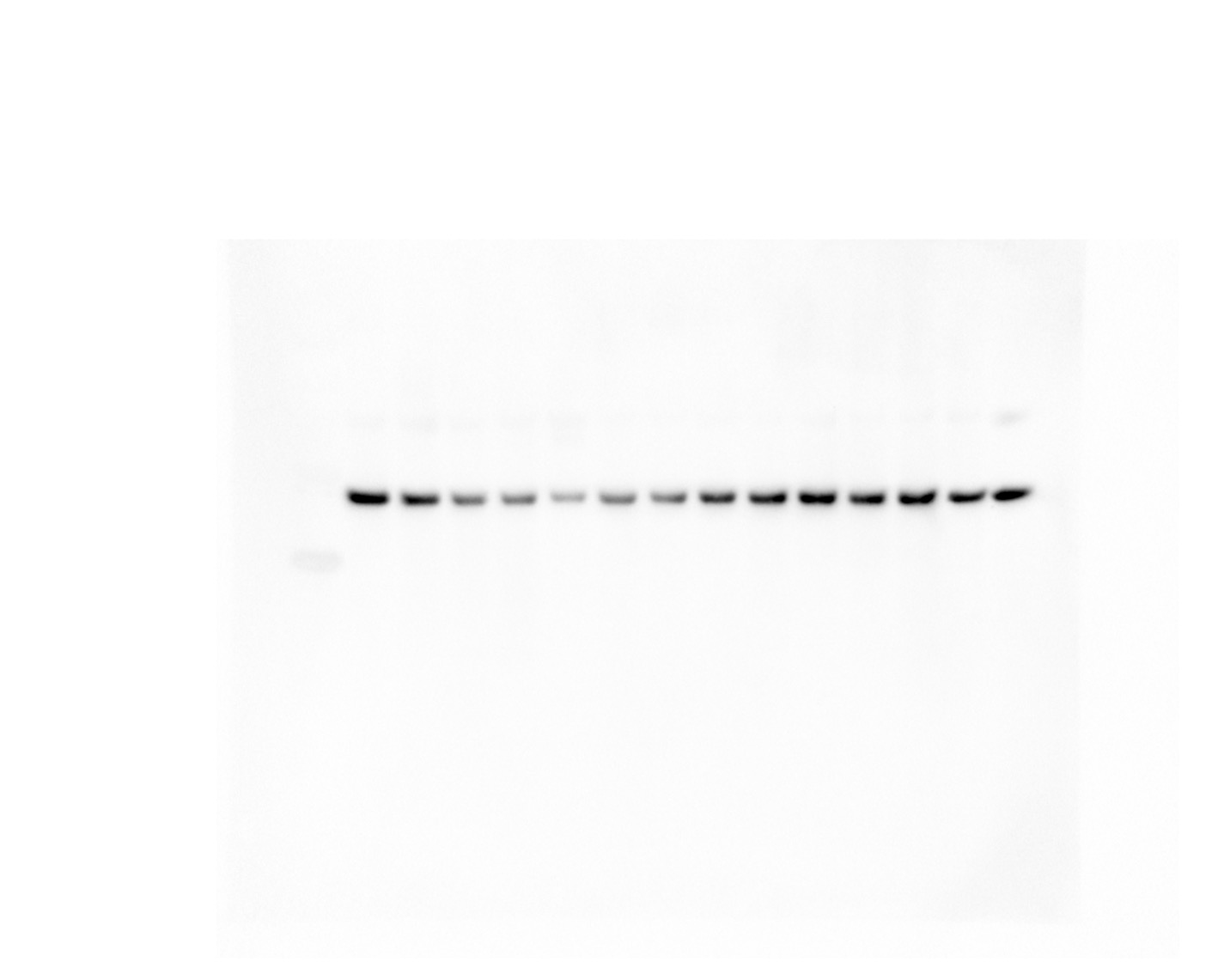

Supplement: Figure 4—figure supplement 1—source data 3. [file elife-96519-fig4-figsupp1-data3.zip › Actin I.jpg]

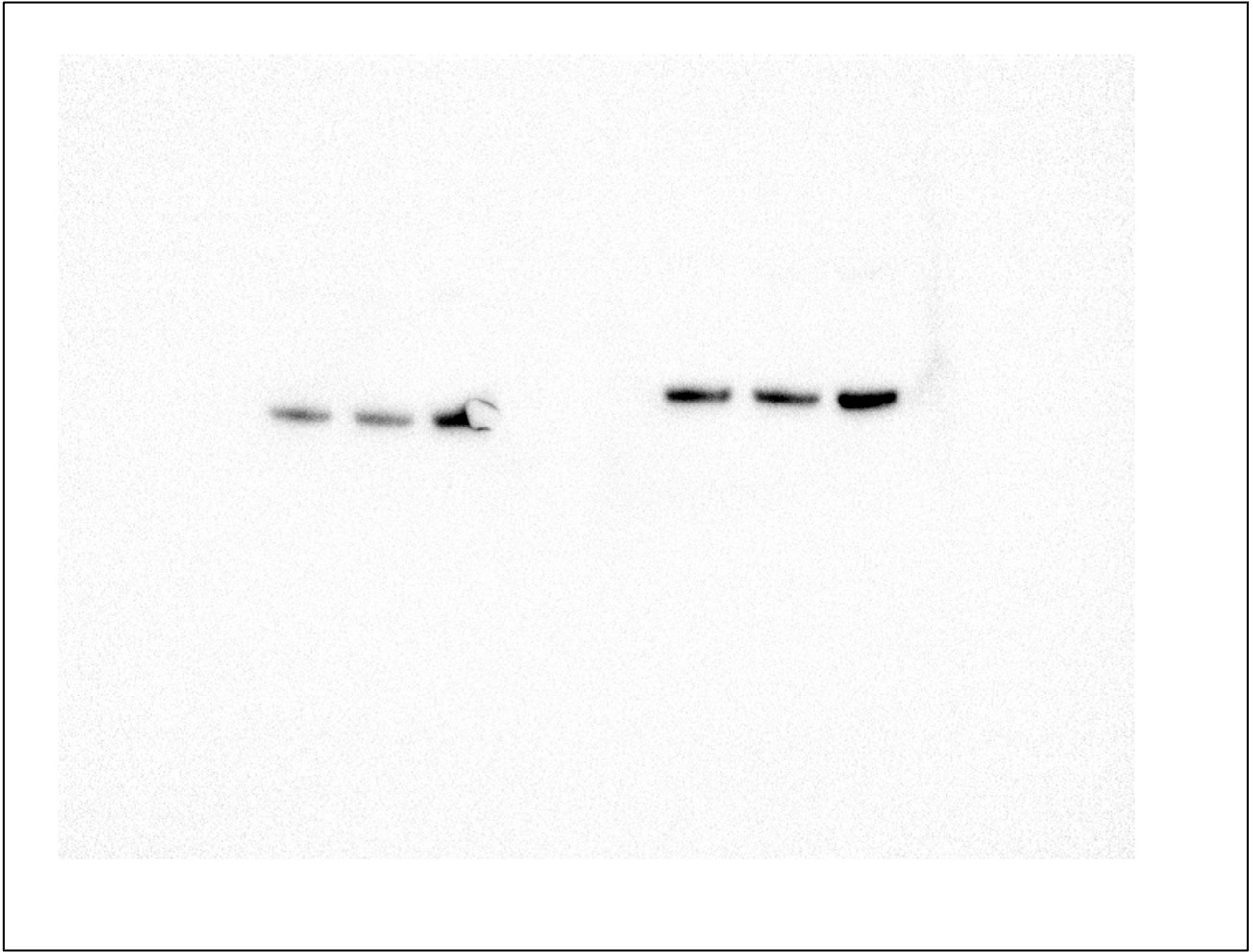

Supplement: Figure 4—figure supplement 1—source data 3. [file elife-96519-fig4-figsupp1-data3.zip › Actin PTPN11 line B.jpg]

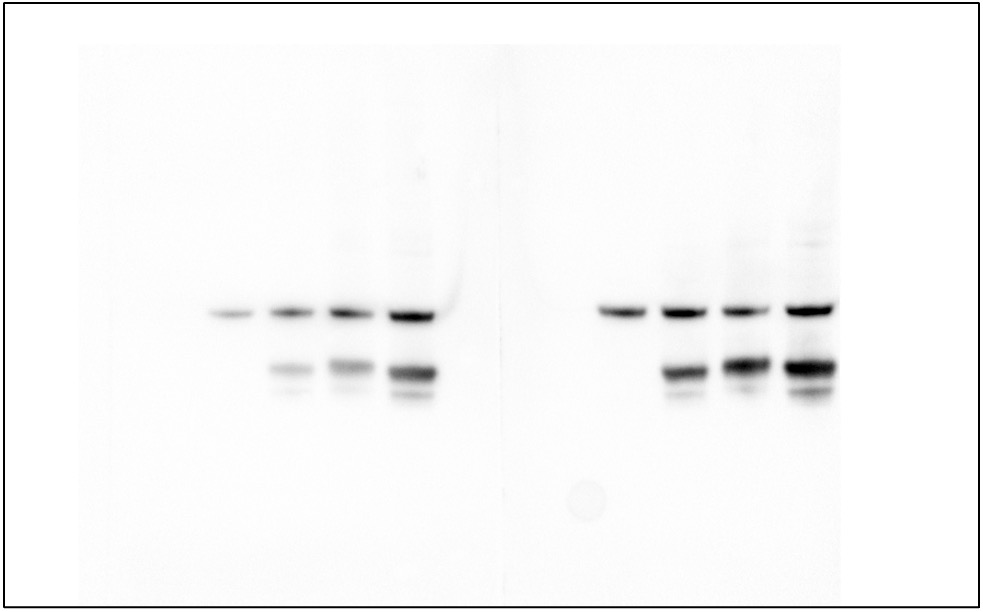

Supplement: Figure 4—figure supplement 1—source data 3. [file elife-96519-fig4-figsupp1-data3.zip › Actin RIT1 line A.jpg]

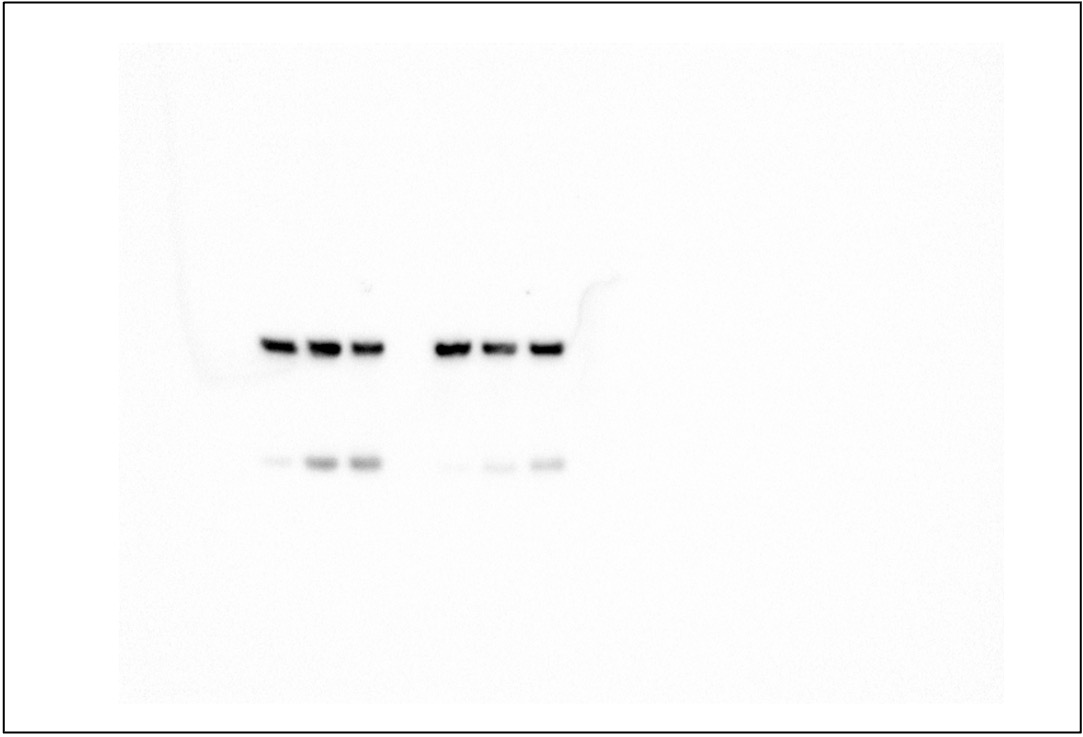

Supplement: Figure 4—figure supplement 1—source data 3. [file elife-96519-fig4-figsupp1-data3.zip › Actin-KRAS line A .jpg]

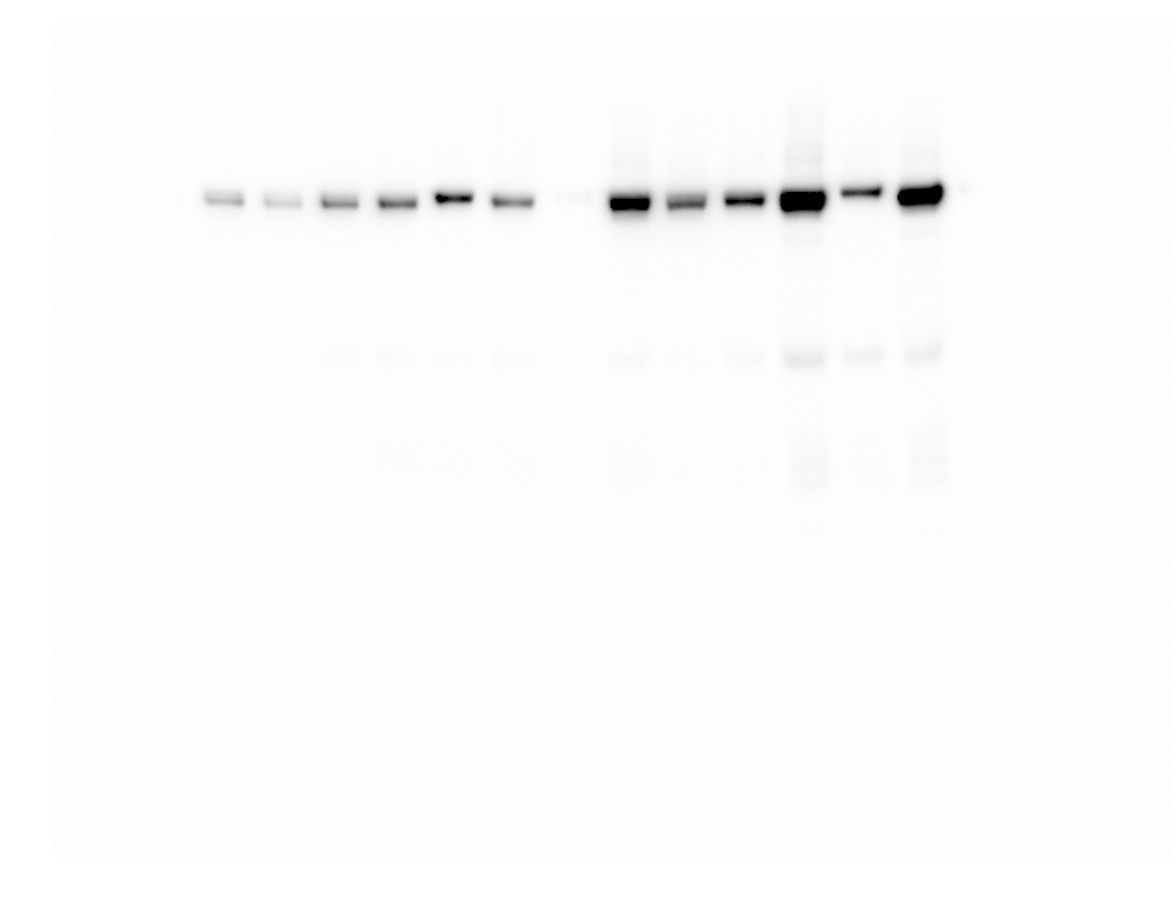

Supplement: Figure 4—figure supplement 1—source data 3. [file elife-96519-fig4-figsupp1-data3.zip › CBL cbl line A.jpg]

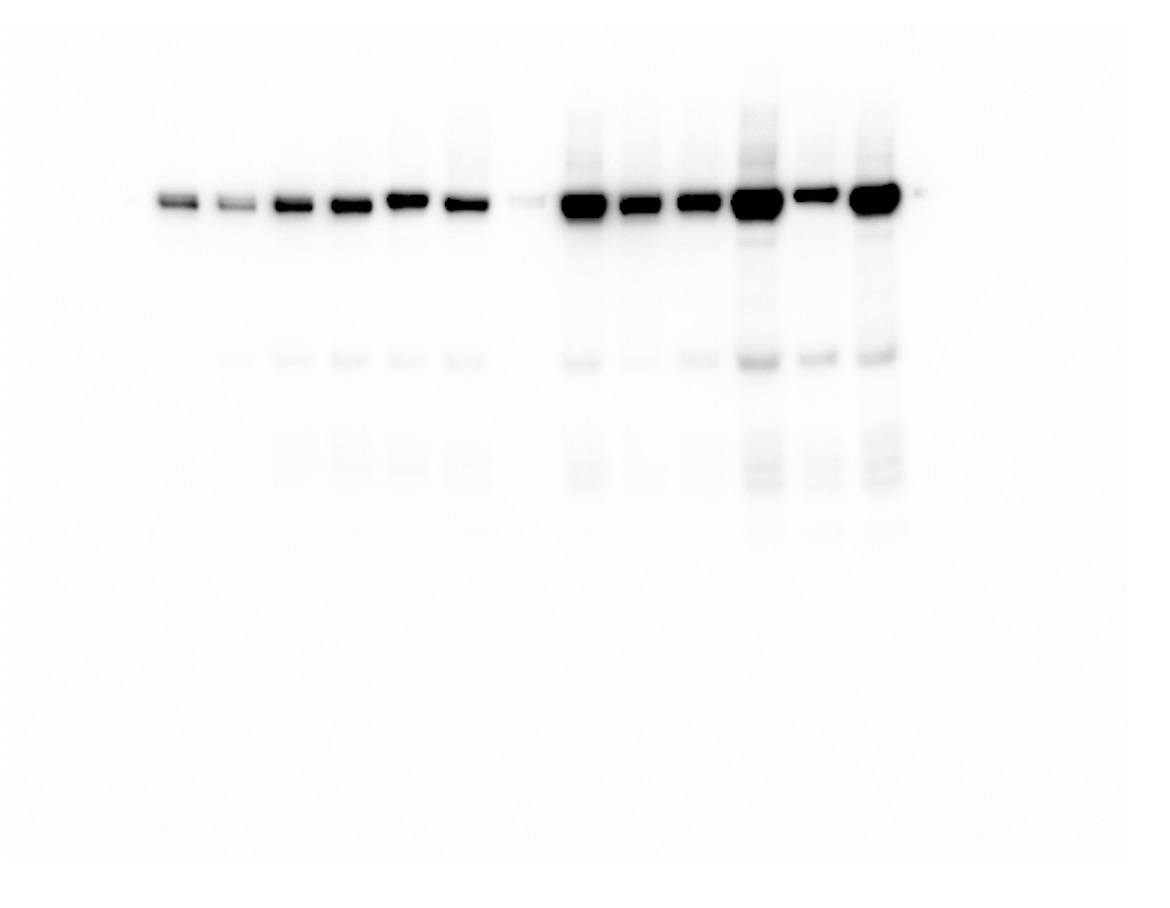

Supplement: Figure 4—figure supplement 1—source data 3. [file elife-96519-fig4-figsupp1-data3.zip › CBL cbl line2 A.jpg]

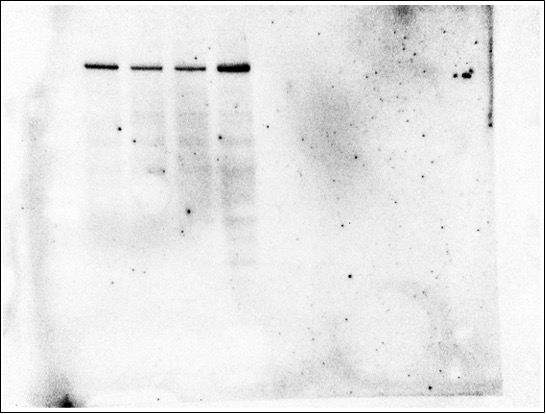

Supplement: Figure 4—figure supplement 1—source data 3. [file elife-96519-fig4-figsupp1-data3.zip › CBL F.jpg]

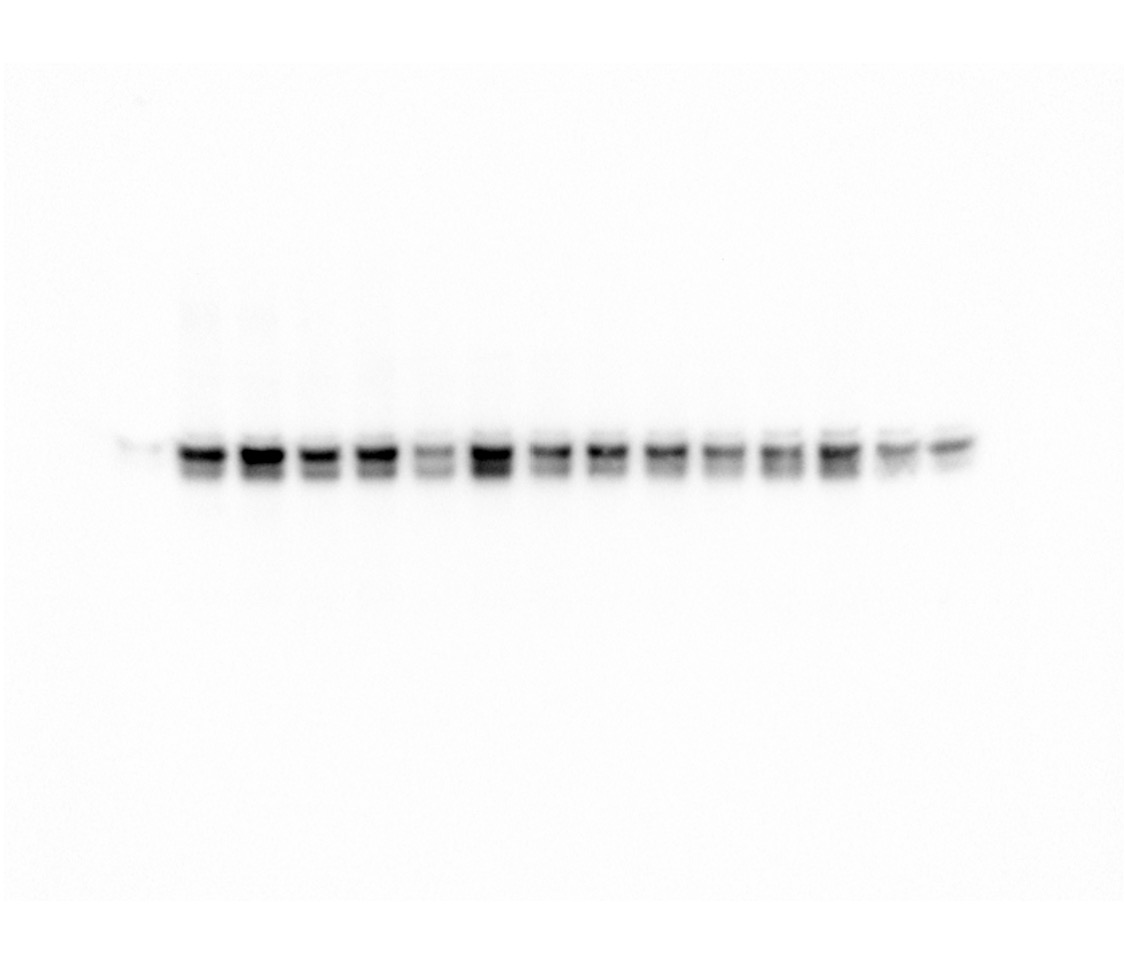

Supplement: Figure 4—figure supplement 1—source data 3. [file elife-96519-fig4-figsupp1-data3.zip › ERK I.jpg]

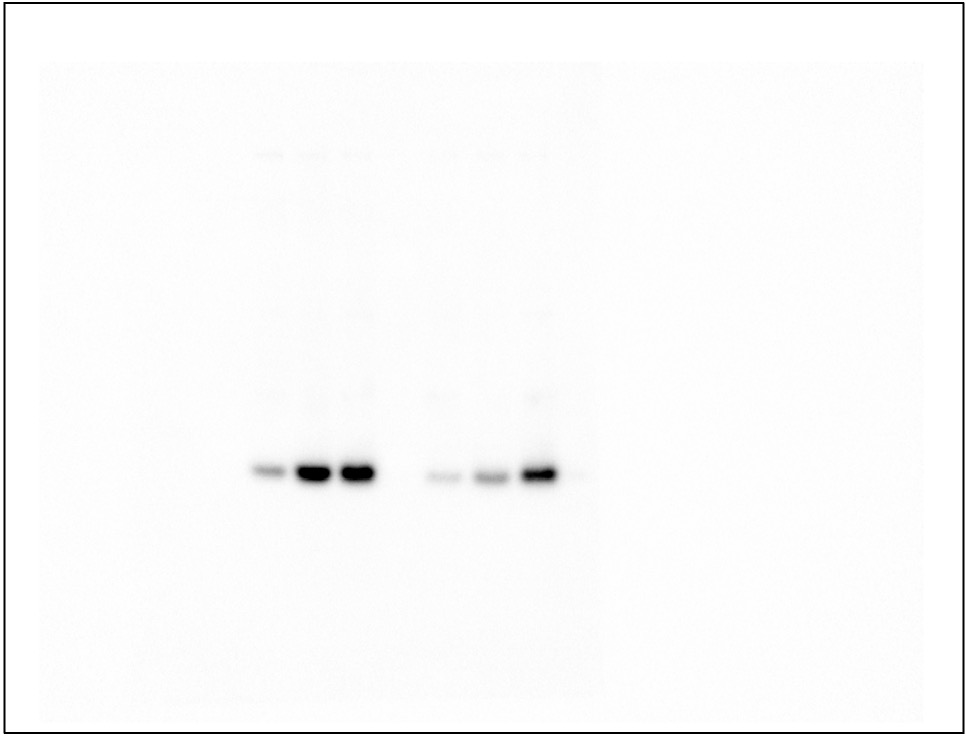

Supplement: Figure 4—figure supplement 1—source data 3. [file elife-96519-fig4-figsupp1-data3.zip › KRAS KRAS line A.jpg]

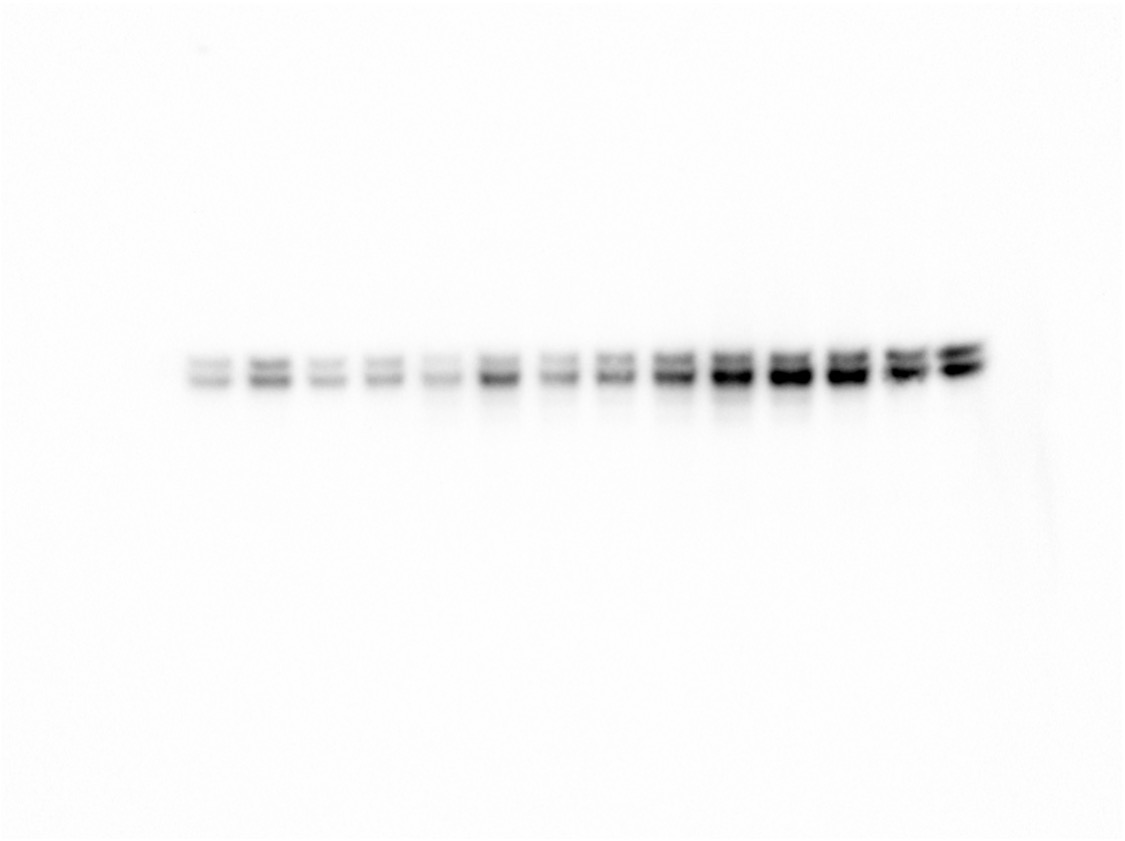

Supplement: Figure 4—figure supplement 1—source data 3. [file elife-96519-fig4-figsupp1-data3.zip › pERK I.jpg]

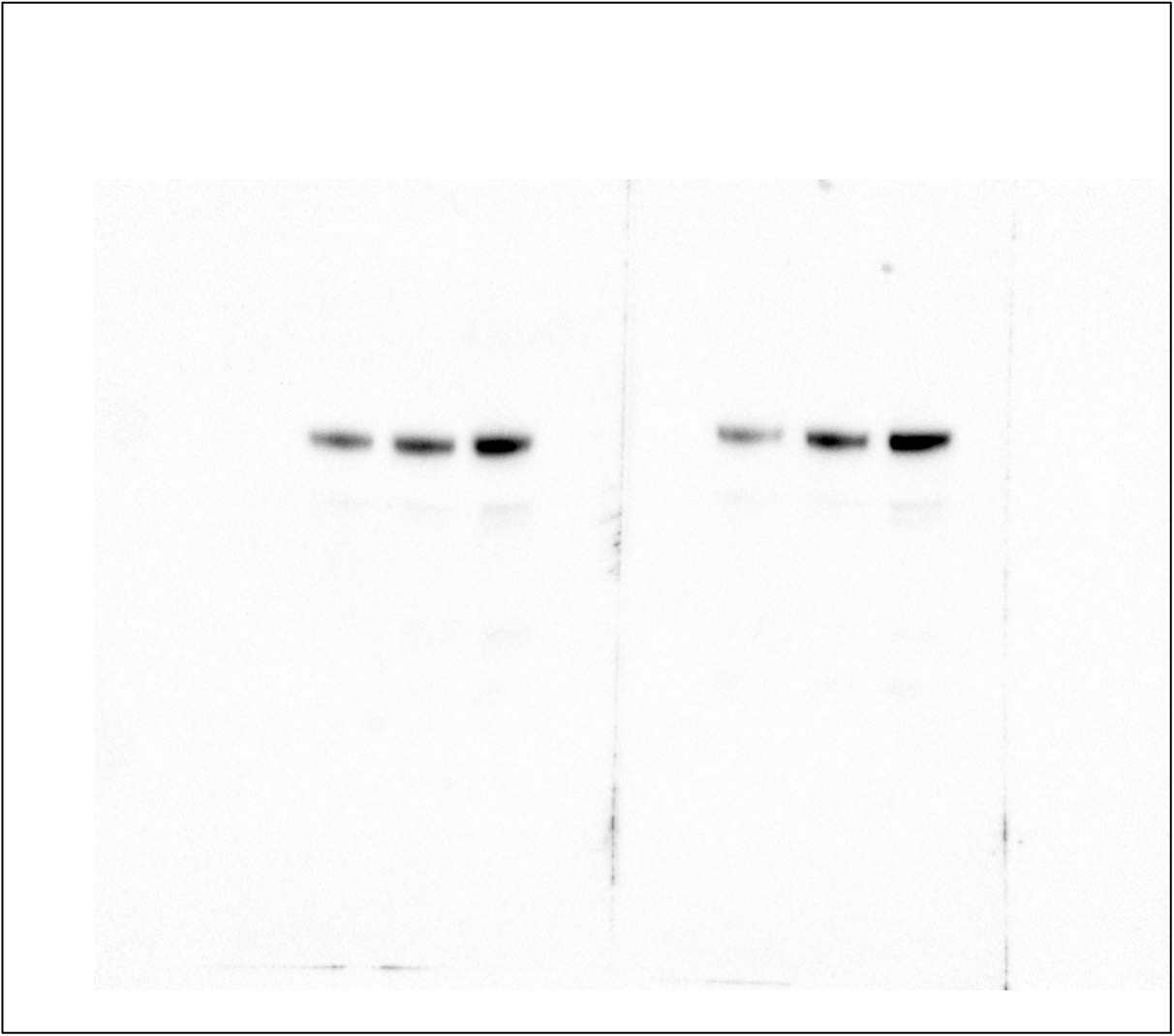

Supplement: Figure 4—figure supplement 1—source data 3. [file elife-96519-fig4-figsupp1-data3.zip › PTPN11 PTPN11 line B.jpg]

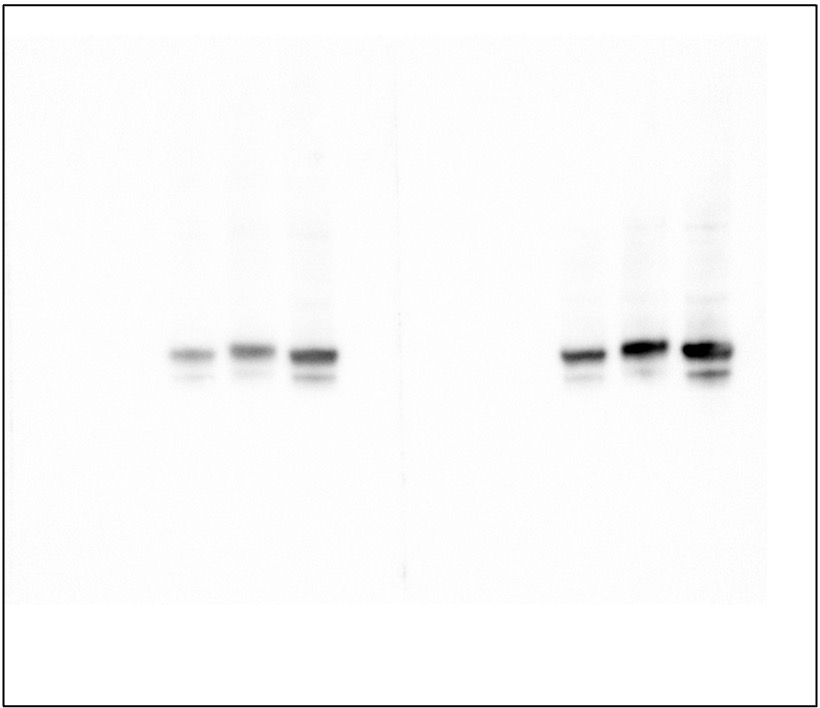

Supplement: Figure 4—figure supplement 1—source data 3. [file elife-96519-fig4-figsupp1-data3.zip › RIT1 RIT1 line A.jpg]
